# Precise determination of input-output mapping for multimodal gene circuits using data from transient transfection

**DOI:** 10.1371/journal.pcbi.1008389

**Published:** 2020-11-30

**Authors:** Christoph Stelzer, Yaakov Benenson

**Affiliations:** Department of Biosystems Science and Engineering, ETH Zurich, Mattenstrasse 26, Basel 4058, Switzerland; Duke University, UNITED STATES

## Abstract

The mapping of molecular inputs to their molecular outputs (input/output, I/O mapping) is an important characteristic of gene circuits, both natural and synthetic. Experimental determination of such mappings for synthetic circuits is best performed using stably integrated genetic constructs. In mammalian cells, stable integration of complex circuits is a time-consuming process that hampers rapid characterization of multiple circuit variants. On the other hand, transient transfection is quick. However, it is an extremely noisy process and it is unclear whether the obtained data have any relevance to the input/output mapping of a circuit obtained in the case of a stable integration. Here we describe a data processing workflow, Peakfinder algorithm for flow cytometry data (PFAFF), that allows extracting precise input/output mapping from single-cell protein expression data gathered by flow cytometry after a transient transfection. The workflow builds on the numerically-proven observation that the multivariate modes of input and output expression of multi-channel flow cytometry datasets, pre-binned by the expression level of an independent transfection reporter gene, harbor cells with circuit gene copy numbers distributions that depend deterministically on the properties of a bin. We validate our method by simulating flow cytometry data for seven multi-node circuit architectures, including a complex bi-modal circuit, under stable integration and transient transfection scenarios. The workflow applied to the simulated transient transfection data results in similar conclusions to those reached with simulated stable integration data. This indicates that the input/output mapping derived from transient transfection data using our method is an excellent approximation of the ground truth. Thus, the method allows to determine input/output mapping of complex gene network using noisy transient transfection data.

## Author summary

One of the key features of a gene circuit is its input/output behavior. A few earlier publications attempted to develop methods to extract this behavior using transient transfection of circuit components in mammalian cells. However, the hitherto developed methods are only suitable for circuit with monomodal output distribution. Moreover, the relationship between the extracted I/O mapping and the "ground truth" that would have obtained with stably-integrated circuits, has not been addressed. Here we explore cell populations easily identifiable in flow cytometry data, namely, the peaks of fluorescent readout distribution in cells binned by the common expression value of the transfection reporter, or marker, gene. Using numerical simulations, we find that the distribution of circuit copy number in these cells deterministically depends on marker fluorescence in the noise-dependent manner. Moreover, we find that this is true also in the case of bi-modal output distribution. Using the peaks of input and output distributions, we are able to reconstruct the I/O mapping of the circuit and relate it to the I/O mapping of the stably-integrated circuit. The reconstruction is enabled by a new computational method we call *PFAFF*. The method is extensively validated with forward-simulated flow cytometry data from stable and transient transfections, with up to seven different circuits. The results show excellent correlation between the I/O behavior extracted by PFAFF from simulated transient transfection data, and the data simulated for stably integrated circuit.

## Introduction

Many synthetic gene circuits fall into the category of information-processing systems that convert molecular inputs to molecular outputs according to a specific relationship [1], often called a “program”. A typical design-build-test cycle of a synthetic gene circuit requires that an input/output (I/O) relationship be characterized in order to confirm circuit function. Direct characterization is possible when both the input(s) and the output(s) can be measured simultaneously in single cells. Using fluorescent reporters, it is possible to obtain the collection of single-cell data points of the type [*input*; *output*], including for natural regulatory pathways, either by direct observation using staining, or by creating synthetic analogs of natural circuits furnished with fluorescent reporters [2–8]. It has emerged that the output forms a distribution at a single cell level for each input [8–10], resulting in a two-dimensional probability distribution for the entire I/O relationship, rather than a curve, due to cell-to-cell variation in parameter values. Nevertheless, after averaging, these noisy data sets usually collapse to Hill functions or to multimodal, two-value functions [11].

Characterization of a circuit that is stably integrated in a cell genome or on replicating fixed-copy episomal vectors is usually straightforward, provided that the inputs and the outputs can be measured. Thus, till now most of characterized input/output behaviors were obtained in bacteria or yeast, where genome manipulation is relatively facile. However, obtaining such “ground truth” information in mammalian cells has lagged behind, because it is still very labor-intensive to establish stably integrated multi-gene circuits. Further, properly executed characterization requires multiple accompanying control circuits to serve as baseline, thus requiring that not one but multiple stable cell lines be developed. Even though technologies such as transposon [12,13] and viral delivery [14,15], targeted integration via Zinc finger nucleases (ZFNs) [16], transcription activator-like effector nucleases (TALENs) [17] or clustered regularly interspaced short palindromic repeats (CRISPR)/Cas9 [18,19] are available today, they are still time consuming even in simple cases and become more challenging with the increase in circuit size. Integration locus-specific effects further complicate the characterization.

Transient transfection of gene circuits is a widespread alternative to stably-integrated circuit characterization in mammalian cells [20–24]. Multiple plasmids, each carrying a single gene, can be co-delivered, leading to correlated gene copy numbers in individual cells. The expression of gene products in dividing cell cultures typically reaches quasi-steady-state two to three days post transfection, and decreases on days four to six due to plasmid dilution [24,25]. The advantage of the transient transfection is that the genome integration-specific effects can be ignored; likewise, secondary effects that often result from having a few genes close to each other on the genome do not play a role because each gene is encoded on a separate plasmid. On the other hand, transient transfections are extremely noisy due to large copy number variation (1–150 transcriptionally-active gene copies per cell [6]), which makes direct interpretation of the resulting datasets impossible. Accordingly, the standard analysis applied to transient transfection data is at the cell population level, with average values of inputs and outputs reported for entire cell populations (see, Schreiber et al. [26] as a representative case). This works sufficiently well for logic gene circuits that are often characterized at the extremes of their input values. Progress towards deriving continuous input/output relationship using transient transfection data has been made in the past [6,27–29]. However, these methods were designed to extract monomodal input/output curves and are thus unsuitable for bi- or multimodal circuits. Moreover, there has been very little computational or experimental validation of these results, in particular, how they compare to stably-integrated systems, and to what the different input/output curves correspond.

We sought to develop a data analysis strategy that would determine input/output relationships from transient transfection data and be applicable to all steady-state networks, including those with bi-modal or bi-stable behavior. We also sought to understand what exactly constitutes a comparable "stable integration" scenario for the information extracted from raw transient transfection data. Accordingly, we first investigate the gene copy number distributions in cell populations that are easily identifiable in flow cytometry-like datasets. We address the question numerically and find a number of important reproducible trends that make it possible to draw reliable and interpretable conclusions from data obtained in transient transfections, and map them back to their stable-integration counterparts. In order to validate the method, we perform *in-silico* experiments by simulating flow cytometry data expected in a transient transfection using dynamic circuit models. At the same time, we use the exact same models and parameter values to simulate the input/output relationship for the case of stable genomic integration. With this approach, we are able to evaluate whether our workflow, when applied to transient transfection data, results in an input/output behavior that is similar to the input/output behavior one would expect for a stable integration.

As benchmarks, we focus on three-node gene network motifs that have been extensively studied earlier [30,31]. We find excellent correspondence between the results of our processing pipeline and the ground truth of the stable integration. Importantly, we are able to capture multi-value, bi-modal responses. Therefore, the method described here can be used to analyze transient transfection data and draw conclusions about the underlying input/output mapping in complex gene circuits, without the need to construct stable cell lines.

## Results

### Statistical framework for transient transfection

In what follows, we define a gene circuit as a set of *N* genes

$$g=[g_{1},g_{2},\ldots ,g_{N}]$$ (1)

and their corresponding gene products

$$G=[G_{1},G_{2},\ldots ,G_{N}]$$ (2)

in which a subset of components

$$I=[I_{1},I_{2},\ldots ,I_{Q}]\subset G$$ (3)

is defined as *input* and a subset of components

$$O=[O_{1},O_{2},\ldots ,O_{P}]\subset G$$ (4)

is defined as *output*.

Further, consider a cell that harbors a gene circuit, either in a stably-integrated or transiently-delivered fashion, such that a gene *g*_*i*_ is present in *k*_*i*_ copies in that cell, and the entire set of copy numbers is a vector

$$k=[k_{1},k_{2},\ldots ,k_{N}]$$ (5)

Hereafter, we consider only interactions between circuit components that have been intentionally engineered (*i*.*e*., chromatin-related effects do not interfere with circuit function in the stable case), and assume that the biochemical parameters describing individual interactions do not change between stably integrated and transiently-delivered components. Even though individual cells in a population of stable clones may behave differently, *e*.*g*., through stochastic effects [32], we expect the aggregate statistics of different clones containing identical circuit copy number to be similar to the aggregate statistics of cells transiently transfected with the same circuit copy number. Therefore, when considering stable clones, we imply an idealized "averaged" clone in which the integrated circuit is governed by the same parameters as the transiently transfected circuit. It then follows that if we apply the same input ***I*** to a population of cells that all harbor the circuit with the copy number vector ***k***, and allow the cells to arrive at a steady state in the stable case and to the quasi-steady state in the transient case (see S1 Text “*In-silico* time-courses”), then the outputs ***O*** will form the same statistical distribution, which can be mono- or multimodal [33], in both cases. Reporting the distribution of ***O*** for various inputs ***I*** would conclude the characterization of a stably-integrated circuit, because all cells harbor the exact same vector ***k***, which can be engineered or experimentally determined *post factum* after clonal isolation.

In the transient transfection experiment, while the values of ***I*** and ***O*** could be collected for individual cells, the underlying values of ***k*** are unknown because the process of transient delivery is extremely noisy. The only way to derive useful data from transient transfections is to deduce, at least for a subset of cells, their ***k*** values, and group together data from cells with similar values of ***k***. If this can be accomplished, the input and output values measured in these cells will be similar to the values one would have obtained with a circuit stably integrated at ***k*** copies. Below, we develop a statistical description of a transient co-transfection process, which leads us to identify cells residing in binned modes of input and output distributions as cells for which the copy number vector ***k*** can be estimated.

We start with the statistical description of a multi-plasmid co-transfection of *N* constitutively expressed and mutually independent genes *g*_1_,*g*_2_,…,*g*_*N*_, generating (fluorescent) protein products *O*_1_,*O*_2_,…,*O*_*N*_. Note that there is no input in this system, so every protein product can be called an “output”. Available data [6] suggest that experimentally-observed distributions of a protein level expressed from a constitutive promoter are lognormal. The mean of the distribution is proportional to the gene copy number *k*_*i*_ with the promoter-dependent global proportionality coefficient *β*_*i*_ being independent of *k*_*i*_; the standard deviation *σ*_*i*_ of the log-transformed protein level distribution may, in principle, depend on a copy number, but we assume it to be constant in the following equations. Let us define a random variable *Y*_*i*_ as the log-transformed protein output of the gene *g*_*i*_.

$$Y_{i}=\ln\;O_{i}$$ (6)

*Y*_*i*_ is distributed normally and its mean/mode *μ*_*i*_, and standard deviation, *σ*_*i*_, relate as follows to the mean of the underlying pre-transformed distribution

$$E[O_{i}]=\beta_{i}k_{i}=exp\left(\mu_{i}+\frac{\sigma_{i}^{2}}{2}\right)$$ (7)

and therefore

$$\mu_{i}=ln(\beta_{i}k_{i})-\frac{\sigma_{i}^{2}}{2}$$ (8)

The conditional probability density function (*pdf*) of *Y*_*i*_ given a gene copy number *k*_*i*_ and parameter *β*_*i*_ is then described by

$$p(Y_{i}|k_{i})=\frac{1}{\sqrt{{2\pi \sigma }_{i}^{2}}}exp\left(-\frac{{\left(Y_{i}-{ln(\beta }_{i}k_{i})+\frac{\sigma_{i}^{2}}{2}\right)}^{2}}{2\sigma_{i}^{2}}\right)$$ (9)

For a vector of gene copy numbers ***k*** = [*k*_1_,*k*_2_,…,*k*_*N*_], a conditional multivariate *pdf* of the log-transformed protein expression values ***Y*** = [*Y*_1_,*Y*_2_,…,*Y*_*N*_], provided that each gene generates its own protein output independently of each other, is described by a multivariate normal distribution without covariances (for simplicity, we assume *σ*_*i*_ to be the same for all genes and use a symbol *σ* in what follows):

$$p_{Y}(Y|k)=p_{Y}([Y_{1},Y_{2},\ldots ,Y_{N}]|k)=\prod_{i=1}^{N}p(Y_{i}|k_{i})=\frac{1}{\sigma^{N}\sqrt{{2\pi }^{N}}}exp\left(-\sum_{i=1}^{N}\frac{{\left(Y_{i}-{ln(\beta }_{i}k_{i})+\frac{\sigma^{2}}{2}\right)}^{2}}{2\sigma^{2}}\right)$$ (10)

To describe the distribution of gene copy numbers in a transient transfection, we introduce an independent parameter *m* that we call “multiplicity of transfection”. Indeed, there is no experimental data that concerns the probability distribution of genes in a co-transfection, and it likely depends on the exact transfection protocol. Therefore, we make a baseline assumption about the *pdf* of the gene copy number vector ***k*** as a multivariate normal distribution without covariance that depends on the multiplicity of transfection. The standard deviation of each gene copy number distribution scales linearly with multiplicity, with the scaling factor *ε*. To account for gene combinations that deviate from an equimolar ratio, a parameter *a*_*i*_ describes the relative abundance of a gene. In this case, one gene is assigned as the "reference" with *a*_*i*_ = 1.

$${p_{g}(k|m)=p}_{g}([k_{1},k_{2},\ldots ,k_{N}]|m)=\frac{1}{{\left(\varepsilon m\right)}^{N}\sqrt{{2\pi }^{N}}\prod_{i=1}^{N}a_{i}}exp\left(-\sum_{i=1}^{N}\frac{{\left(k_{i}-a_{i}m\right)}^{2}}{2{\left(\varepsilon a_{i}m\right)}^{2}}\right)$$ (11)

Lastly, *m* itself can be distributed non-uniformly according to its *pdf p*(*m*). Distributions such as Poisson [34], Gamma [35], lognormal [36] or even a combination of them [37], have been used to describe the process of DNA or viral vector delivery to cells. For transient lipofection of DNA, lognormal distributions approximate experimental data well, and therefore

$$p(m)=LN(\mu_{m},\sigma_{m})=\frac{1}{m\sigma_{m}\sqrt{2\pi }}exp\left(-\frac{{\left(ln(m)-\mu_{m}\right)}^{2}}{2{\sigma_{m}}^{2}}\right)$$ (12)

One of the genes and its protein product is assigned the role of, respectively, a reference gene and a reference protein (sometimes called “transfection marker”); let us assume it is *k*_1_, with gene product *O*_1_ and its log-transformed counterpart *Y*_1_. Thus, by definition *a*_1_ = 1. To derive the conditional marginal *pdf p*(*Y*_*i*_|*Y*_1_), which is the probability to find the value *Y*_*i*_ of the log-transformed protein *O*_*i*_ expression in a cell in which the log-transformed reference protein expression equals *Y*_1_, we first drop irrelevant variables from Eq 10 to evaluate joint probability distribution of log-transformed protein levels [*Y*_*i*_,*Y*_1_] given the underlying gene copy numbers [*k*_*i*_,*k*_1_]:

$$p(\left[Y_{i},Y_{1}]|[k_{i},k_{1}\right])=\frac{1}{\sigma^{2}\sqrt{{2\pi }^{2}}}exp\left(-\frac{{\left(Y_{i}-{ln(\beta }_{i}k_{i})+\frac{\sigma^{2}}{2}\right)}^{2}}{2\sigma^{2}}-\frac{{\left(Y_{1}-{ln(\beta }_{1}k_{1})+\frac{\sigma^{2}}{2}\right)}^{2}}{2\sigma^{2}}\right)$$ (13)

In turn the gene copy numbers are conditionally dependent on the multiplicity parameter *m*:

$$p([k_{i},k_{1}]|m)=\frac{1}{{a_{i}(\varepsilon m)}^{2}\sqrt{{2\pi }^{2}}}exp\left(-\frac{{\left(k_{i}-a_{i}m\right)}^{2}}{2{\left(\varepsilon a_{i}m\right)}^{2}}-\frac{{\left(k_{1}-m\right)}^{2}}{2{\left(\varepsilon m\right)}^{2}}\right)$$ (14)

The global joint probability distribution *p*([*Y*_*i*_,*Y*_1_]) of *Y*_*i*_ and *Y*_1_ over all values of [*k*_*i*_,*k*_1_] is obtained by integrating over all values of [*k*_*i*_,*k*_1_]:

$$p(\left[Y_{i},Y_{1}\right])=\iint p(\left[Y_{i},Y_{1}]|[k_{i},k_{1}\right])p(\left[k_{i},k_{1}\right]m)p(m)d[k_{i},k_{1}]dm$$ (15)

It is customary, as already done earlier [6,38], to bin cells that share the same *Y*_1_, the log-transformed value of *O*_1_, because this is the only readout independent of the other components, as it is a self-contained gene expressed from a constitutive promoter. We follow this approach here: cells binned according to their *Y*_1_ value will exhibit certain log-transformed distributions of the other proteins *Y*_2_,…,*Y*_*N*_. Knowing the joint *pdf* (Eq 15), one can derive the conditional probability of *Y*_*i*_ given *Y*_1_ (that is, the *pdf* of *Y*_*i*_ among cells that express *Y*_1_ log-transformed copies of the reference protein), as follows:

$$p(Y_{i}|Y_{1})=\frac{p(\left[Y_{i},Y_{1}\right])}{p(Y_{1})}=\frac{\iint p(\left[Y_{i},Y_{1}]|[k_{i},k_{1}\right])p(\left[k_{i},k_{1}\right]|m)p(m)d[k_{i},k_{1}]dm}{\iint p(Y_{1}|k_{1})p(k_{1}|m)p(m)dk_{1}dm}$$ (16)

The mode of this distribution, *i*.*e*., the most probable value of *Y*_*i*_ given the reference *Y*_1_, can be found by solving the equation

$$\frac{dp(Y_{i}|Y_{1})}{dY_{i}}=0$$ (17)

Let us denote this most probable value as $Y_{i}^{MODE}(Y_{1})$. The value of $Y_{i}^{MODE}(Y_{1})$ can be determined experimentally as a mode of *Y*_*i*_ distribution after binning the cells according to their *Y*_1_ value. The equation may have more than one solution, corresponding to multimodal probability density function from Eq 16.

This bring us to the most relevant question of this section: What is the distribution of the gene copy number *k_i_* for the cells that reside in the mode(s) of *Y_i_*, and what is the most probable value of *k_i_*? To answer this question, we evaluate the conditional probability *p*(*k*_*i*_|*Y*_*i*_) according to Bayes’ theorem:

$$p(k_{i}|Y_{i})=\frac{p(Y_{i}|k_{i})p(k_{i})}{p(Y_{i})}=\frac{\frac{1}{\sqrt{{2\pi \sigma }^{2}}}exp(-\frac{{\left(Y_{i}-{ln(\beta }_{i}k_{i})+\frac{\sigma^{2}}{2}\right)}^{2}}{2\sigma^{2}})\int p(k_{i}|m)p(m)dm}{\iint p(Y_{i}|k_{i})p(k_{i}|m)p(m)dk_{i}dm}$$ (18)

In order to find the most probable copy number $k_{i}^{MODE}(Y_{i}^{MODE}(Y_{1}))$ we solve the equation (or equations, when $Y_{i}^{MODE}(Y_{1})$ takes more than one value)

$$\frac{dp(k_{i}|Y_{i}^{MODE}(Y_{1}))}{dk_{i}}=0$$ (19)

Knowing the most probable gene copy numbers in the cells residing in the modes of log-transformed protein distributions allows us to correlate the data to what might be obtained in cells with stably integrated constructs harboring similar gene copy numbers.

### Numerical analysis of transient co-transfection of constitutively expressed genes

An analytical solution of Eq 19 does not exist, and we solve it using numerical simulations. To this end, we performed *in-silico* simulations of a transient co-transfection containing multiple (*N* = 5) independent genetic constructs (Methods). The change in protein expression over time, ${\dot{O}}_{i}$, of each gene *g*_*i*_ can be described by an ordinary differential equation (ODE) with kinetic parameter ${\bar{b}}_{i}$, gene copy number *k*_*i*_ and degradation rate *δ*_*i*_

$$\dot{O_{i}}={\bar{b}}_{i}k_{i}-\delta_{i}O_{i}$$ (20)

In the steady state, i.e. ${\dot{O}}_{i}=0$, the steady-state level of *O*_*i*_ is proportional to *k*_*i*_ with the global coefficient of proportionality $\beta_{i}={\bar{b}}_{i}/\delta_{i}$ and identical to Eq 7:

$$O_{i}^{s.s.}=\beta_{i}k_{i}$$ (21)

Iterating multiple times to simulate multiple single cells *j* (1≤*j*≤*C*, where *C* is the total number of simulated "cells"), we draw the multiplicity *m*_*j*_ from a lognormal distribution (Eq 12) with parameters that roughly fit experimental data (see below) and initialize gene copy number vectors

$$k_{j}=[k_{j1},k_{j2},\ldots ,k_{jN}]$$ (22)

according to Eq 11 with pre-set parameters (Methods). To create log-normal protein distributions given ***k***_***j***_ according to Eq 10, for each *k*_*ji*_ in ***k***_***j***_, local proportionality factor *b*_*ji*_ is drawn from a log-normal distribution:

$$LN\left(\ln\left(\beta_{i}\right)-\frac{\sigma^{2}}{2},\sigma \right)$$ (23)

with fixed *β*_*i*_ values (Methods, S1 Table); the values of *σ* are fixed for a given simulation run and systematically varied between 0.00 and 0.32 in different runs. A value

$$O_{ji}=k_{ji}b_{ji}$$ (24)

is the level of protein *O*_*i*_ in cell *j* (S1 Fig).

The generated *in-silico* dataset (S2A Fig) is similar to a flow cytometry dataset what one would obtain in a transient co-transfection experiment of constitutively-driven genes. In order to confirm that the parameters, and in particular the values of *σ* are realistic, we transiently co-transfected five plasmids, each expressing constitutively different fluorescent protein (*O*_1_: SBFP2, *O*_2_: Cerulean, *O*_3_: Citrine, *O*_4_: mCherry and *O*_5_: iRFP; Methods) (S2B Fig). We find that the standard deviation of the log-transformed protein expression distribution in cells pre-binned on similar values of the reference protein, which we denote σ*,

$${\sigma^{*}=\sigma }_{Y_{i}|Y_{1}}$$ (25)

depends on *Y*_1_, and indeed ranges between 0.1–0.3 (S2C Fig). Higher values of σ*are observed as very low *Y*_1_ values, and they plateau towards 0.1 for larger *Y*_1_. Accordingly, the range of *σ* values used in the simulations, and given that *σ*<σ*, constitutes a realistic range for the gene expression variability due to "intrinsic noise".

**Fig 1:**
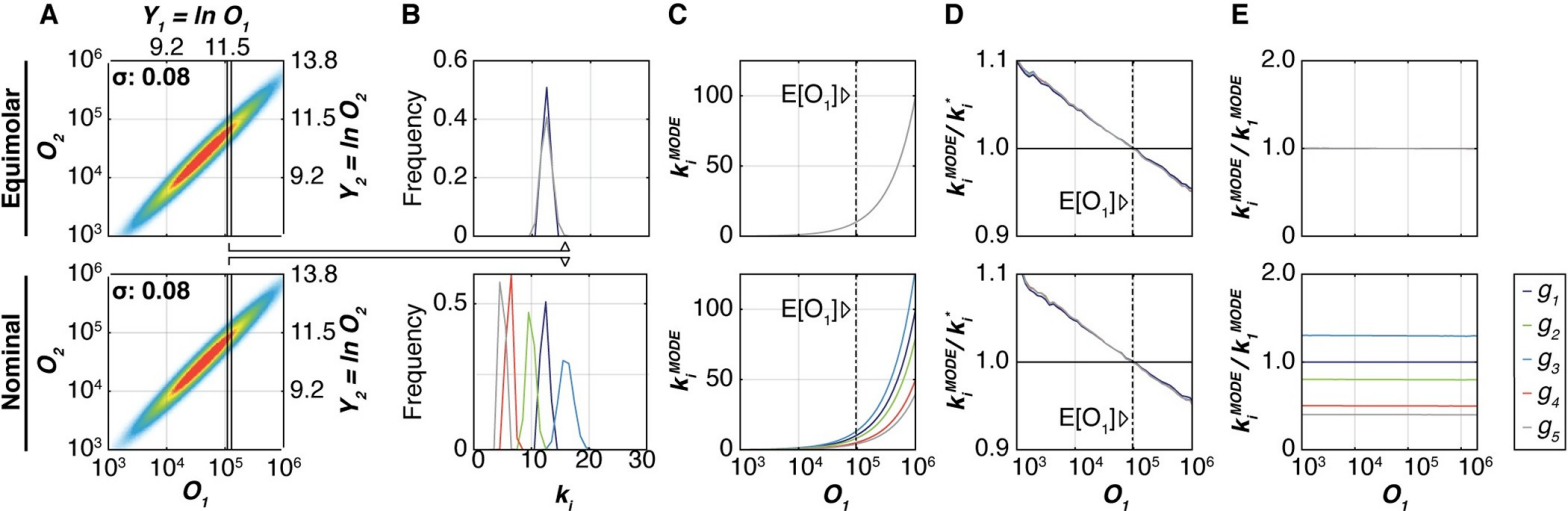
*In-silico* simulation of multiple gene co-transfections. *In-silico* simulations shown for two cases with parameter settings *σ* = 0.08, *ε* = 0.04 and *m* = 10: equimolar (*a*_1_:*a*_2_:*a*_3_:*a*_4_:*a*_5_ = 1.0: 1.0: 1.0: 1.0: 1.0; top row) and "nominal" (*a*_1_:*a*_2_:*a*_3_:*a*_4_:*a*_5_ = 1.0: 1.3: 0.8: 0.5: 0.4; bottom row) ratio of gene mix. **(A)** Density plots show the amount of expressed proteins *O*_2_ versus *O*_1_. Solid black lines indicate the edges of an example bin for the transfection reference protein *O*_1_. **(B)** The plots show the distribution of gene copy numbers in cells whose *O*_1_ values fall into the bin shown in panel A. Copy number distributions corresponding to different genes *g*_*i*_ are shown using different colors (legend on the very right of the figure). In the equimolar case, gene copy number distributions overlap, while in the "nominal" case they are separated. **(C)** The modes of the copy number distributions are plotted versus the median signal of the transfection reference protein *O*_1_ in all bins (colored lines). The dash-dotted line marks the mean (*E*[*O*_1_]) of the global *O*_1_ distribution. **(D)** For each bin of the transfection reference protein *O*_1_, the modes of the gene copy number distributions from each gene *g*_*i*_ are determined numerically. The ratio of the numerically-determined mode of the copy number distribution $k_{i}^{MODE}$ and the anticipated copy number $k_{i}^{*}$ are computed and plotted versus the corresponding *O*_1_ values in individual bins. The global mean of *O*_1_ is shown with a dash-dotted line. **(E)** Ratios of gene copy number modes $k_{i}^{MODE}$ relative to the gene copy number mode of the transfection reference protein $k_{1}^{MODE}$, as a function of the *O*_1_ median value in the bins.

Next, we simulate transient co-transfections using two different gene ratios; (i) equimolar and (ii) a ratio of 1.0:1.3:0.8:0.5:0.4, the latter following some fine-tuning in a parallel experimental project (manuscript under preparation), to generate a joint *pdf p(****Y****)* (Figs 1A, S3A, S3B, S4A and S4B). We use these datasets to solve Eqs 17 and 19 numerically, that is, determine the $Y_{i}^{MODE}(Y_{1})$ and $k_{i}^{MODE}(Y_{i}^{MODE}(Y_{1}))$. To do so, we bin cells that share a log-transformed reference protein value *Y*_1_, evaluate the conditional *pdf p*(*Y*_*i*_|*Y*_1_) and, first, determine numerically the value of $Y_{i}^{MODE}(Y_{1})$. Second, we retrospectively look up the values of *k*_*i*_ in cells whose *Y*_*i*_ and *Y*_1_ expression levels lie in the vicinity of a vector [$Y_{i}^{MODE}(Y_{1});Y_{1}$]. The empirical distribution of *k*_*i*_ (Figs 1B, S3C and S4C) is $p(k_{i}|Y_{i}^{MODE}(Y_{1}))$ from Eq 19, and the mode of the gene copy number distribution, $k_{i}^{MODE}(Y_{i}^{MODE}(Y_{1}))$, or $k_{i}^{MODE}(Y_{1})$, is determined numerically (Figs 1C, S3D and S4D, Methods).

According to Eq 7 there is a simple, linear relation between *O*_*i*_ and the gene copy number *k*_*i*_, linking them via the global coefficient of proportionality *β*_*i*_. The global coefficient of proportionality can be determined experimentally using *e*.*g*., a calibrated Western blot to measure the absolute amount of protein and calibrated qPCR to measure absolute mean internalized gene copy numbers. For the transfection reference protein, we introduce the variable $k_{1}^{*}$ that corresponds to the gene copy number that one would "naïvely" anticipate to be the most probable value leading to a particular *Y*_1_, given *β*_1_:

$$k_{1}^{*}(Y_{1})=\frac{exp(Y_{1})}{\beta_{1}}$$ (26)

For the other log-transformed outputs *Y*_*i*_ the naïvely anticipated copy number in cells that express certain level of the reference protein *Y*_1_, is defined by a similar relationship:

$$k_{i}^{*}(Y_{1})=\frac{exp(Y_{1})}{\beta_{1}}{\cdot a}_{i}$$ (27)

Given that the value of *Y*_1_ and *β*_1_ are the only “knowable” parameters, it is of interest to ask how the actual copy numbers relate to these anticipated values. Using our simulated datasets, we compute the ratio between numerically found $k_{i}^{MODE}(Y_{1})$ and the anticipated copy number $k_{i}^{*}(Y_{1})$ from Eq 27 (Fig 1D) as a function of *Y*_1_. We find that the deviation from the anticipated value is a decreasing monotonous function of *Y*_1_ with the following properties: (1) The deviation is always positive for values of *Y*_1_<ln *E*[*O*_1_]; (2) the deviation is essentially zero when *Y*_1_ = ln *E*[*O*_1_], and (3) it is negative for *Y*_1_>ln *E*[*O*_1_]. Further, the absolute magnitude of the deviation increases with increasing *σ* (S3E and S4E Figs). However, for all noise levels, the deviation is zero at the global mean of the *O*_1_ distribution, *E*[*O*_1_], and

$$\frac{k_{i}^{MODE}(ln(E[O_{1}]))}{k_{i}^{*}(ln(E[O_{1}]))}\approx 1$$ (28)

We further analyzed the ratio of the modes of gene copy number distributions $k_{i}^{MODE}$ to $k_{1}^{MODE}$ in cells that reside in the close vicinity of the log-transformed expression vector $\left[Y_{1},Y_{2}^{MODE}(Y_{1}),Y_{3}^{MODE}(Y_{1}),\ldots ,Y_{N}^{MODE}(Y_{1})\right]$. The ratio stays constant for almost the entire range of *Y*_1_ values (Figs 1E, S3F and S4F). Since the naïve estimate and the numerical mode of the absolute gene copy number coincide at the global mean of the *O*_1_, both the relative and the absolute abundance of the gene copy numbers can be deduced with high certainty in cells that express *O*_1_ around its global mean. This is true regardless of the chosen distribution of *m* and *β*_*i*_. Simulations that employ Poisson, Gamma or lognormal distributions show a strikingly similar effect (S5 Fig). Appropriate experimental techniques allow measuring both the protein copy number [39] and the gene copy [6] number in the cells residing at the global mean of *O*_1_, making it possible to determine the value of *β*_1_ experimentally and therefore extrapolate directly to the ground truth expected in the stable cell line with the similar gene copy number.

### Numerical analysis of transient co-transfection of non-trivial gene circuits

Next, we consider the case when the same genes, apart from the transfection reference protein gene *g*_1_, encode a set of genes interacting in a circuit. Depending on the circuit, log-transformed distribution *Y*_*i*_ of protein *O*_*i*_ in cells pre-binned on the value of *Y*_1_ may exhibit mono-, bi- or multimodality. We may consider the joint probability distribution of the vector of independent constitutive genes and their gene products, ***k***∙***Y***, as a baseline state of any circuit. When the genes are interconnected (not including the reference gene *g*_1_ and its log-transformed product *Y*_1_), this baseline distribution is transformed because the values of ***Y*** are no longer independent. However, the values of ***k*** remain the same, because they represent the exact same underlying process of DNA delivery, and only the ***Y*** values change relative to the independent, constitutive values. We hypothesize that despite the fact that the values of ***Y*** are no longer independent of *k*_*j*_ for *i*≠*j*, ***k*** vectors corresponding to the (possible multiple) multivariate modes of ***Y***|*Y*_1_, would not deviate far from the ***k*** vectors obtained in the case of independent co-transfection. We further hypothesize that this deviation will decrease as the noise in the system increases to biologically-plausible levels.

**Fig 2:**
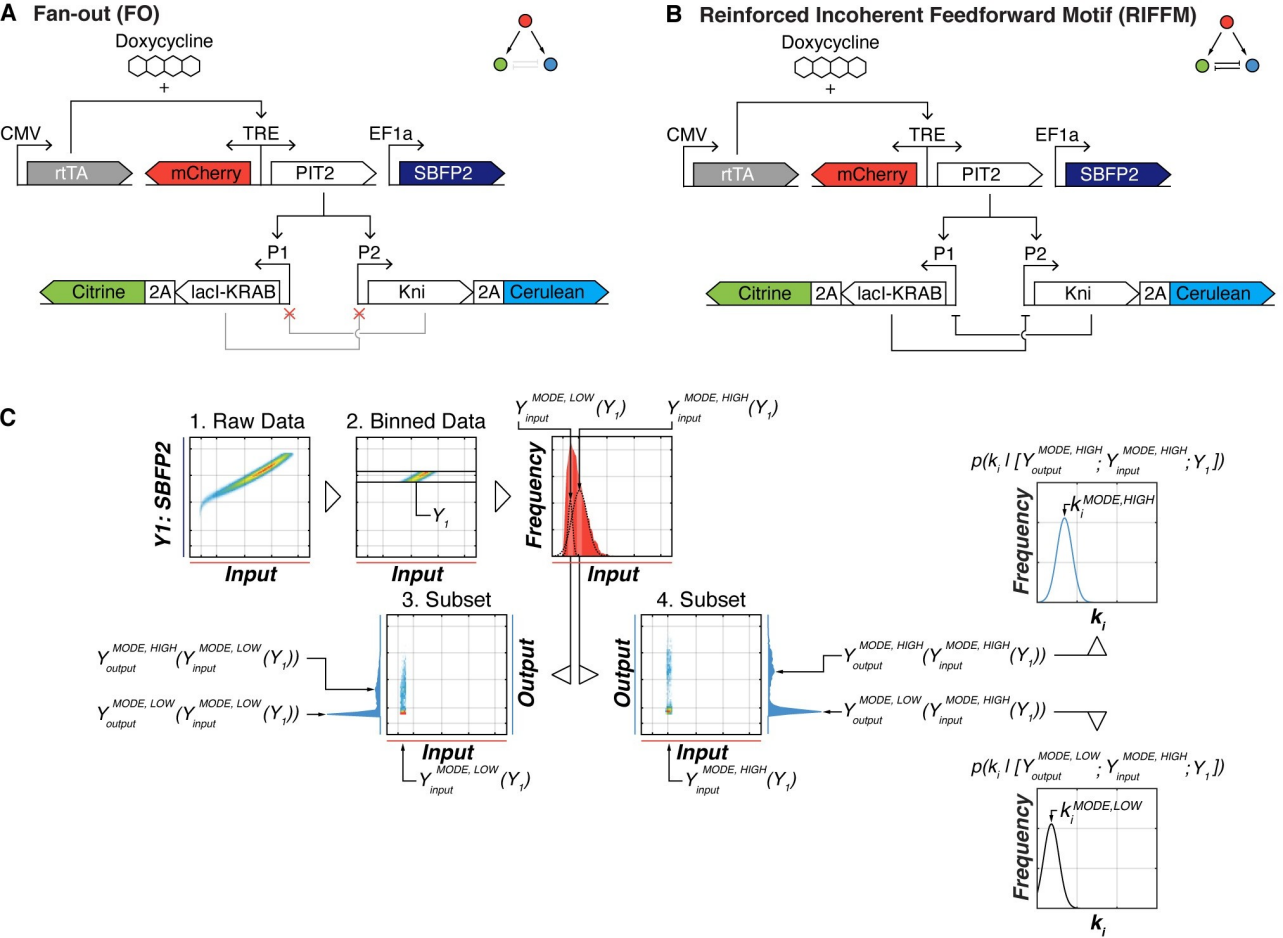
Schematics of gene circuits FO and RIFFM and analysis outline. (**A**)| Monomodal/fan-out (FO) and (**B**) bi-modal (RIFFM) gene circuit. The circuits are composed of five independent genes. Constitutively-expressed transcription factor rtTA co-induces PIT2 and the fluorescent protein mCherry in a Doxycycline-dependent fashion. PIT2 in turn activates two promoters P1 and P2, which express a transcriptional repressor lacI-KRAB and Kni, respectively, co-expressed via P2A linkers with Citrine and Cerulean fluorescent reporters. In the FO circuit the repressors do not interact due to mutated promoter binding sites, while in the RIFFM circuit they repress each other, establishing a mutual inhibition. (**C**) Graphical illustration of all steps to find the modes within our simulated data sets. The raw flow cytometry like data (**1**) is binned by the transfection marker (i.e. *Y*_1_: SBFP2). The binned data is isolated and subsequent analysis is done only on this subset (**2**). Afterwards the distribution of the log-transformed input signal (mCherry) within the binned subset is determined and at least one Gaussian is fitted to the distribution. A narrow bin(s) around the mode(s) (black arrows) of the fitted distribution(s) is determined (pink bars), thus obtaining a subset of the originally-binned dataset. The subset around the peaks ($Y_{input}^{MODE,LOW}(Y_{1})$ and $Y_{input}^{MODE,HIGH}(Y_{1})$) are now analyzed individually. For illustrative purposes, we show the input (mCherry) and output (Cerulean) signal of the subset around $Y_{input}^{MODE,LOW}(Y_{1})$ (**3**) and $Y_{input}^{MODE,HIGH}(Y_{1})$ (**4**). The modes of the log-transformed output distributions are identified using a similar peak finding procedure as for the input signal (mCherry). The output histograms and their modes (black arrows) are shown on the sides of plot. Within these modes, we look at the *pdf* of the gene copy number distributions for all circuit genes as well as the transfection reference gene and identify the vectors $k^{MODE,HIGH}=[{k_{1}^{MODE,HIGH},\ldots ,k}_{i}^{MODE,HIGH},\ldots ,k_{N}^{MODE,HIGH}]$ and $k^{MODE,LOW}=[{k_{1}^{MODE,LOW},\ldots ,k}_{i}^{MODE,LOW},\ldots ,k_{N}^{MODE,LOW}]$, respectively.

To test these hypotheses, we simulated two three-node gene circuit architectures (currently being investigated experimentally in a related project, see S6 Fig for the experimental results of the fan-out circuit); a simple monomodal *fan-out* circuit (*FO*; Fig 2A) and a non-trivial *pitchfork bifurcation circuit*, also known as *reinforced incoherent feed forward motif RIFFM* [4,30,40,41] (Fig 2B). The input to the circuit is a transcriptional activator PIT2 [42], whose level is tuned by Doxycycline via an bi-directional TRE promoter that also drives a fluorescent protein mCherry as a proxy for input expression. The first PIT2 target promoter (P1) drives the *D*. *melanogaster* derived transcriptional repressor Knirps (*kni*) and translationally-linked fluorescent protein Cerulean, constituting the first circuit output. The second PIT2 target promoter (P2) drives the transcriptional repressor LacI fused to a KRAB domain and translationally linked to a fluorescent protein Citrine, representing the second circuit output. In *RIFFM* circuit, *kni* is able to repress P2 while LacI is able to repress P1; in *FO*, the mutual repression is eliminated via mutations.

We built mechanistic kinetic models of the circuits *FO* and *RIFFM* (S2 Text "Simple Fan-Out Model" and S3 Text "Detailed Models") and simulated the flow cytometry dataset for multiple transiently transfected cells *j* (1≤*j*≤*C*, where *C* is the total number of simulated "cells"). As above, every gene is encoded on a separate plasmid. We also compared this to a single-plasmid setup with all five genes are located on a single DNA backbone, but saw only a marginal difference in outcomes (S7 Fig). The multiplicity of transfection and the gene copy numbers are simulated as above (S1A Fig); the gene copy numbers become the initial conditions for running a simulation. Differently from that case of constitutive co-transfection, we directly simulate circuit dynamics governed by kinetic parameters ***p***; to simulate the effects of intrinsic gene expression noise, the parameters that govern protein translation rates are sampled independently from a lognormal distributions with nominal parameter values *π*_*i*_ and preset "noise" levels ranging, as above, from 0.00 to 0.32:

$$LN\left(\ln\left(\pi_{i}\right)-\frac{\sigma^{2}}{2},\sigma \right)$$ (29)

For every cell *j*, the drawn parameter values *p*_*ij*_ are used in a dynamic simulation ran to a steady state, with the simulated steady state input and output protein levels corresponding to the readouts from that cell.

First, we simulate mono- and bi-modal gene circuits for a single Doxycycline/input level. Similar to the data analysis above, we bin the cells according to *Y*_1_ value. In the bin, we first focus on the input protein *Y*_*input*_ and identify its mode. Importantly, in the general case the distribution of *Y*_*input*_|*Y*_1_ can be bi-modal, leading to two numerically-found values $Y_{input}^{MODE,HIGH}(Y_{1})$ and $Y_{input}^{MODE,LOW}(Y_{1})$. In this case, we consider separately the cells residing close to the expression vectors $\left[Y_{input}^{MODE,HIGH}(Y_{1});Y_{1}\right]$ and $\left[Y_{input}^{MODE,LOW}(Y_{1});Y_{1}\right]$. Next, for every circuit output we consider the distributions $Y_{output}|Y_{input}^{MODE,HIGH}(Y_{1})$ and $Y_{output}|Y_{input}^{MODE,LOW}(Y_{1})$. These distributions can also be multimodal; in what follows we assume they are bi-modal. We denote the modes of the output distribution corresponding to the high mode of the input $Y_{output}^{MODE,HIGH}(Y_{input}^{MODE,HIGH}(Y_{1}))$ and $Y_{output}^{MODE,LOW}(Y_{input}^{MODE,HIGH}(Y_{1}))$, and use similar notation for the output modes corresponding to the low mode of the input, if the latter is present. Lastly, we consider all cells in the vicinity of the expression vector ${[Y}_{output}^{MODE,HIGH}(Y_{input}^{MODE,HIGH}(Y_{1}));\;Y_{input}^{MODE,HIGH}(Y_{1});\;Y_{1}]$ and ${[Y}_{output}^{MODE,LOW}(Y_{input}^{MODE,HIGH}(Y_{1}));\;Y_{input}^{MODE,HIGH}(Y_{1});\;Y_{1}]$ and evaluate the copy number distribution of every circuit gene as well as the reference gene. These are monomodal distributions, with the modes denoted respectively as $k_{i}^{MODE,HIGH}$ and $k_{i}^{MODE,LOW}$ (see Fig 2C for schematic description of the process). These numerically evaluated values are then compared to the naively anticipated values calculated according to the Eq 27. Note that in these simulations, the transfection reference gene expression is modelled explicitly as a transcription/translation/degradation cascade with corresponding kinetic parameters; the value of *β*_1_ for Eq 27 is calculated according to Eqs 20 and 21.

**Fig 3:**
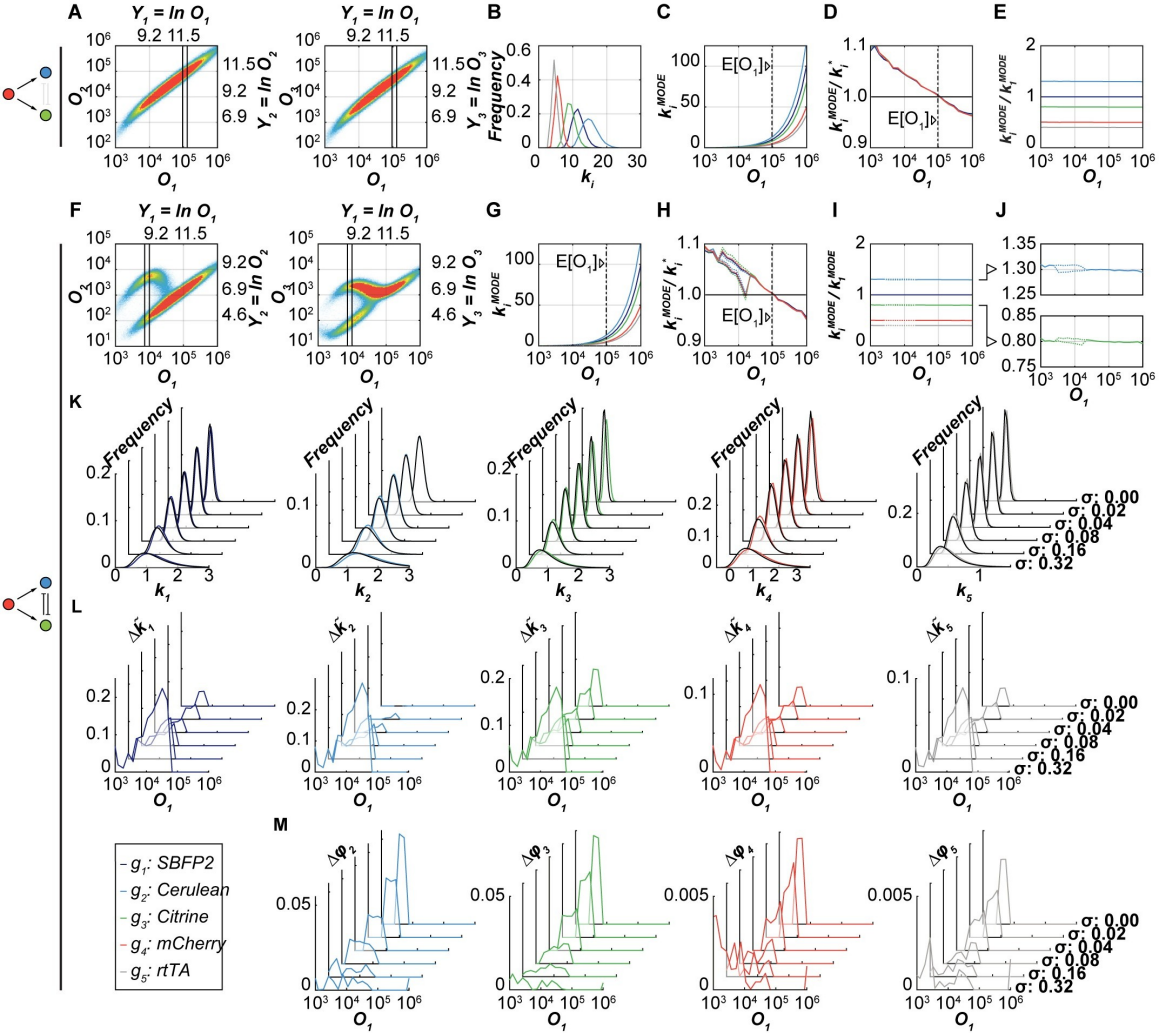
*In-silico* simulation of transiently-transfected gene circuits FO and RIFFM and the copy number analyses at various *σ* noise levels. *In-silico* simulations (global parameters *σ* = 0.08, *ε* = 0.04, *μ*_*m*_ = 1.4979, *σ*_*m*_ = 1.2686 and *a*_1_:*a*_2_:*a*_3_:*a*_4_:*a*_5_ = 1.0: 1.3: 0.8: 0.5: 0.4) shown for two circuits: **A-E** monomodal/fan-out (FO) and **F-M** bi-modal (RIFFM). **(A)** Raw data of the simulated transiently transfected FO circuit. The amount of expressed proteins *O*_2_ (left: Cerulean) or *O*_3_ (right: Citrine) versus *O*_1_ (transfection marker: SBFP2) is shown as a density plot. Solid lines indicate the edges of a transfection marker bin. **(B)** Gene copy number distributions of cells binned by a particular value of log-transformed *O*_1_ in a bin shown in panel **A**. **(C)** The modes of the copy number distributions, $k_{i}^{MODE}$, are plotted versus the median signal of the transfection reference protein *O*_1_ for all bins (colored lines). The dash-dotted line marks the mean (*E*[*O*_1_]) of the global *O*_1_ distribution. **(D)** The ratio of the numerically-determined mode of the copy number distribution $k_{i}^{MODE}$ and the anticipated copy number $k_{i}^{*}$ plotted versus the *O*_1_ values in individual bins. The global mean of *O*_1_ is shown with a dash-dotted line. **(E)** Modes of the gene copy number distributions $k_{i}^{MODE}$ normalized by the mode of the transfection reference gene $k_{1}^{MODE}$ are shown as a function of *O*_1_ values in individual bins. **(F)** Raw data of the simulated RIFFM circuit with bi-modal output *O*_2_ (Cerulean; left) or *O*_3_ (Citrine; right). The black lines indicate a bin within the bimodal range. **(G)** The modes of the copy number distributions, $k_{i}^{MODE,HIGH}$ and $k_{i}^{MODE,LOW}$, are plotted versus the median signal of the transfection reference protein *O*_1_ of all bins (colored lines). Dashed segments indicate the range of *O*_1_ in which the high and low modes do not coincide; the black dash-dotted line indicates the mean (*E*[*O*_1_]) of the global *O*_1_ distribution. **(H)** The ratios of the numerically-determined mode of the copy number distribution $k_{i}^{MODE,HIGH}$ and $k_{i}^{MODE,LOW},$ and the anticipated copy number $k_{i}^{*}$ are plotted versus the corresponding *O*_1_ values in individual bins. Dashed segments indicate the range of *O*_1_ in which the ratios corresponding to high and low modes do not coincide. The global mean of *O*_1_ is shown with a straight dash-dotted line. **(I)** Modes of the gene copy number distributions $k_{i}^{MODE,HIGH}$ and $k_{i}^{MODE,LOW}$, normalized by the mode of the transfection reference gene $k_{1}^{MODE}$ are shown as a function of *O*_1_ values in individual bins. Dashed lines indicate the range of *O*_1_ where the values do not coincide. **(J)** The ratios $k_{i}^{MODE}/k_{1}^{MODE}$ depicted in panel **I** are shown in greater detail for the outputs *O*_2_ (Cerulean; top) and *O*_3_ (Citrine; bottom). **(K)** Fitted gene copy number distributions of the indicated bin in **F** are shown for all genes (left to right) and noise levels *σ*. Black curves indicate the fitted distributions to the low output mode, $k_{i}^{LOW}$, and colored curves the fitted distributions of the high output mode $k_{i}^{HIGH}$. **(L)** Divergence in gene copy number modes $\Delta {\tilde{k}}_{i}$ between high and low protein output modes normalized to the mode of copy number distribution of the transient co-transfection for all noise levels *σ* as a function of *O*_1_ values in individual bins. **(M)** Difference of copy number modes’ ratios *Δϕ*_*i*_ in the high and low protein output modes for all noise levels *σ* as a function of *O*_1_ values in individual bins.

The analysis of the simulated data reveals the following: for the monomodal FO circuit, the behavior of the copy number modes of the input and the output genes is quantitatively identical to what is observed in the simulation of multiple constitutive gene co-transfection (Fig 3A–3E). The bi-modal circuit (Fig 3F) shows its bi-modal behavior at the lower intensities of the transfection reference protein *O*_1_ (10^3^–10^5^). In this range, distributions of gene copy numbers for the high and low modes of output expression are slightly diverging (Figs 3G–3K). They are, however, almost fully overlapping, and their modes differ only by a few percent respectively upwards or downwards relative to the monomodal case, despite large difference in the corresponding protein modes. We quantify the divergence in gene copy number modes between high and low protein output modes by introducing a metric

$$\Delta {\tilde{k}}_{i}={\tilde{k}}_{i}^{HIGH}-{\tilde{k}}_{i}^{LOW}=\frac{k_{i}^{MODE,HIGH}}{{\hat{k}}_{1}^{MODE}}-\frac{k_{i}^{MODE,LOW}}{{\hat{k}}_{1}^{MODE}}$$ (30)

with ${\hat{k}}_{1}^{MODE}$ being the mode of the transfection marker copy number distribution found in the constitutive co-transfection simulation (Fig 1). We observe a steady increase in $\Delta {\tilde{k}}_{i}$ upon an increase in noise level σ (Fig 3L), however, it is less that 10% for realistic levels of noise. Furthermore, we quantify bi-modality-dependent deviations of gene copy number ratios between the high and low output modes and introduce the metric

$${\Delta \phi_{i}=\phi }_{i}^{HIGH}-\phi_{i}^{LOW}=\frac{k_{i}^{MODE,HIGH}}{k_{1}^{MODE,HIGH}}-\frac{k_{i}^{MODE,LOW}}{k_{1}^{MODE,LOW}}$$ (31)

Unlike $\Delta {\tilde{k}}_{i}$, this deviation of gene copy number ratios, *Δϕ*_*i*_, decreases with an increase in the noise level *σ* (Fig 3M). These observations confirm our hypothesis that even in multimodal circuits, the cells that share the same amount of the reference protein, also share very similar gene copy numbers, both in absolute and especially, in relative terms (S8–S10 Figs). Moreover, deviations from the nominal gene copy number ratio decrease with the increase of noise levels *σ*.

### The rationale for extracting input/output relationship from transient transfection data

The analyses above suggest a workflow for analyzing and deducing input/output relationships from transiently-transfected circuits. To summarize the findings so far, we show that cells, which express a certain level of reference protein *O*_1_ and reside at multivariate modes of log-transformed input and output expression, harbor both input and output genes (or plasmids) with the following properties: (1) even for multimodal outputs with large differences in protein level modes, the distribution of the input and output genes copy numbers corresponding to the different output protein modes are almost overlapping, with the copy number modes varying by about 10% for biologically-realistic noise values, and thus can be treated as the same copy number for all practical purposes; (2) the copy number distribution modes’ ratio almost exactly corresponds to the nominal ratio used in a transfection for all reference protein bin values, for both low and high output modes, and the deviation from the nominal ratio decreases with increased noise; (3) the distribution modes’ absolute values exactly match the naïve anticipation (Eq 27) when the reference protein is expressed at the level *E*[*O*_1_]; (4) the distribution modes’ absolute values (for both high and low output modes) deviate from the naïve expectation in a predictable linear fashion as a function of log-transformed reference protein level *Y*_1_, with the magnitude of the deviation increasing with the overall noise level. However, because the actual noise level and thus the degree of deviation can be quantified experimentally, even in cells that lie away from *E*[*O*_1_] the copy numbers can be estimated not only in relative but also in absolute terms. We show that this holds for a case of a complex circuit that generates bi-modal output distribution, with only slight deviations of the copy number modes from the expectation. Accordingly, by analyzing the input and output values in the cells that reside in the multivariate modes of circuit inputs’ and outputs’ distributions (after binning by the log-transformed reference protein value *Y*_1_), we should be able to extract the information about the input/output response of the circuit that is comparable to the stable cell line harboring the circuit at the copy number derived from *Y*_1_ according to Eq 26 and corrected by the measure of deviation that depends on the noise level.

### Overview of the workflow validation procedure

To validate the workflow suggested above, we simulate transient transfection and stable integration for *FO* and *RIFFM* circuits for a wide range in circuit input levels using the exact same ODE model, with the input modulated via varying Dox level; otherwise the parameter randomization is performed as described above according to Eq 29. To simulate a stable integration dataset, we initialize a copy number vector ***k*** such that the ratio between individual genes corresponds to the nominal ratio of the transient transfection, and the absolute copy number of the reference gene is set to different fixed values corresponding to the bins used for transient transfection data analysis (S11 Fig; Methods). After the datasets are simulated, we extract input/output relationships corresponding to various bins of log-transformed values of the (transfection) reference protein *Y*_1_ from the simulated transient transfection data, as described in detail in the next section. This is compared to the results of the stable integration simulation performed for the copy numbers that correspond to those reference protein levels. The simulation of the stable integration scenario generates an input/output “cloud”, as has also been demonstrated experimentally [8,43,44]. The cloud can be used "as is" for the purpose of comparison, or it can also be processed via output mode identification for different input levels and building averaged curves. The process is illustrated schematically in S12 Fig.

**Fig 4:**
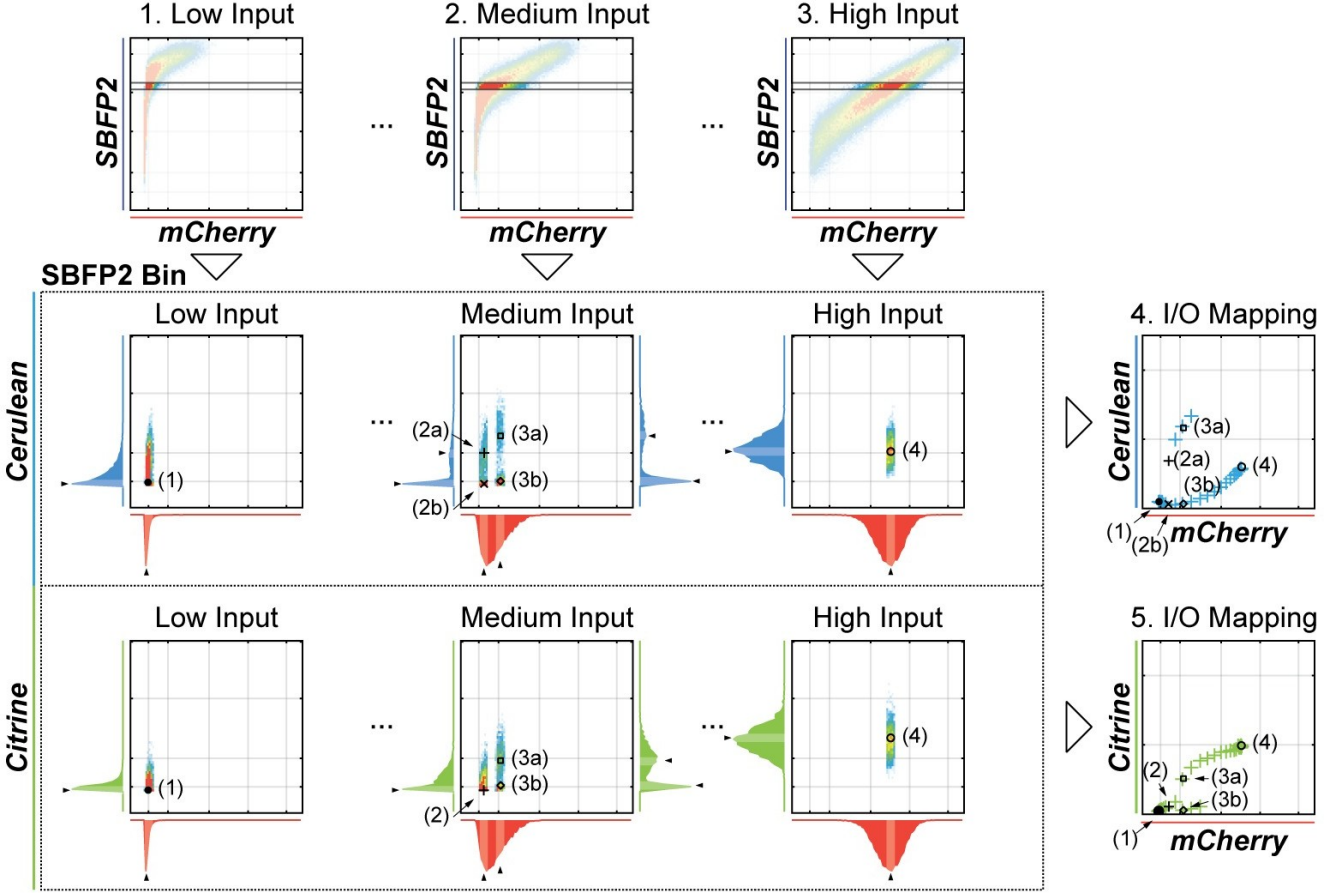
Peak Finder Algorithm For Flow cytometry (PFAFF) analysis strategy. The steps of finding peaks in distributions of a binned data as shown in Fig 2C are repeated for every input induction level (i.e. Doxycycline level; here three representative cases for low (**1**), medium (**2**) and high (**3**) input modulation levels are depicted) for each output (here, Cerulean and Citrine). Initially, a bin of the transfection reference protein (here, SBFP2) is determined and all downstream analyses only deals with cells residing in this bin. The plots in the dashed box show the example workflow of applying the peak finding to the individual input modulation levels. The density plots depict the binned data and the adjacent histograms show the distributions of their respective input and output proteins. Black wedges indicate the modes of the (convoluted) distributions and black markers indicate their location on the density plots. The [*input*; *output*] mode pairs, identified in this workflow (markers), derived from the raw data corresponding to the different input modulation level are plotted on the input/output mapping charts the output Cerulean (**4**) and Citrine (**5**).

The input/output relationship is generated for each level of log-transformed reference protein *Y*_1_. We make use of the datasets simulated with different Doxycycline levels and thus different amounts of input expressed per gene copy. (Note that Doxycycline does not affect the gene copy number or the expression of the transfection reference protein, it is not a direct input and is not a part of the input/output relationships that we seek.) For a given *Y*_1_ bin, a single transfection experiment simulation with a fixed Doxycycline value generates (for a bimodal case) at most four points on the input/output curve: [$Y_{input}^{MODE,LOW};\;Y_{output}^{MODE,LOW}(Y_{input}^{MODE,LOW})$]; $\left[Y_{input}^{MODE,LOW};\;Y_{output}^{MODE,HIGH}(Y_{input}^{MODE,LOW})\right]$; ${[Y}_{input}^{MODE,HIGH};\;Y_{output}^{MODE,LOW}(Y_{input}^{MODE,HIGH})]$; and [$Y_{input}^{MODE,HIGH};\;Y_{output}^{MODE,HIGH}(Y_{input}^{MODE,HIGH})$]. In most cases, the input will only exhibit a single mode that we denote for uniformity $Y_{input}^{MODE,HIGH}$ and thus only two points will be generated for a bi-modal case, and one for a monomodal case. For this same reference protein bin, we repeat the procedure defined earlier (Fig 2C) for every Doxycycline level and generate multiple [*input*; *output*] pairs that cover the entire input range. The procedure can be done for any desired reference protein bin, thus showing circuit behavior for different absolute gene copy number of its components (Fig 4).

The fact that every transient transfection performed with a certain Doxycycline level generates only up to four, and usually one or two, points on the input/output relationship curve is slightly counterintuitive because a flow cytometry plot would reveal wide distribution of the input values. However, this distribution results from the variability in the copy number of the input gene in the cells and is therefore irrelevant to the determination of the input/output relationship. In order to characterize an entire curve, there must be a practical way to modulate input expression per gene copy and repeat the experiment multiple times, every time with a different degree of modulation. This can be done with Doxycycline as in our case; when this is not feasible, one can mimic input modulation by systematically changing the relative dosage of a constitutive input-expressing gene, or use a series of constitutive promoters of varying strengths. In another observation, when extracting the modes, the high output modes corresponding to both the low and the high input modes fall on the same curve when plotted against the input values; the same is true for the low output modes (S13 Fig). This is not surprising, because the input value is the only determinant of the output. Therefore, we pool high and low output modes, respectively, and interpret them as the averaged input/output relationship of a circuit; when the behavior is bimodal, two curves are generated.

### Validation using direct simulation data

**Fig 5:**
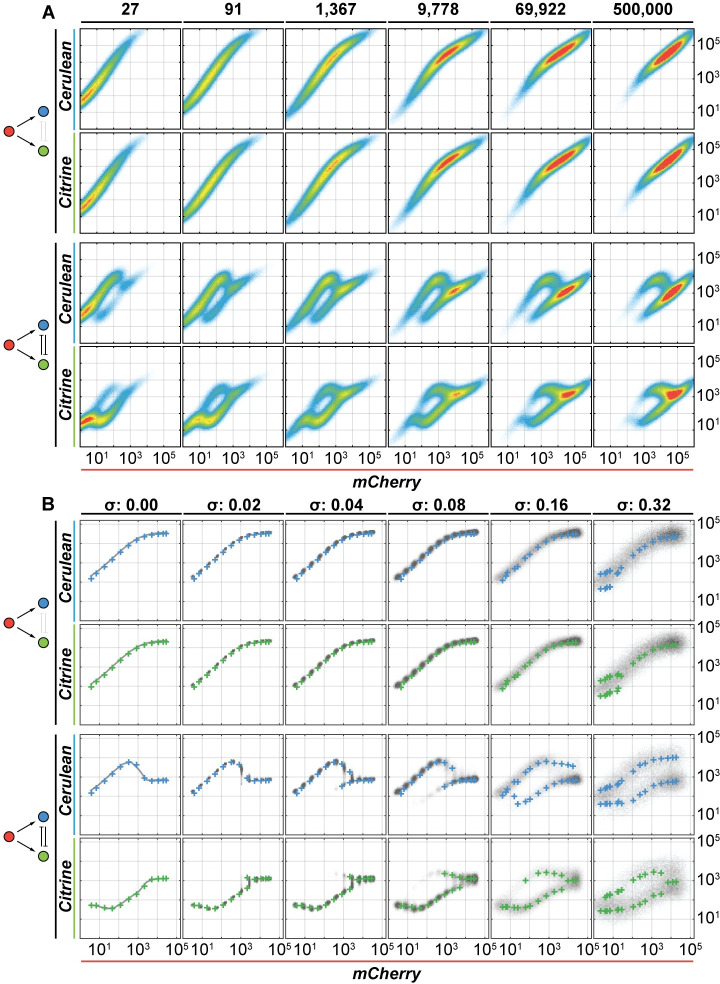
Results of PFAFF analysis on simulated FO and RIFFM flow cytometry data set. **(A)** Raw simulated transient transfection data for circuits FO and RIFFM at noise level *σ* = 0.16, modulated by different input levels of Doxycycline (columns). Each circuit (FO, RIFFM) is represented by two rows of charts depicting the input versus their respective output signals. **(B)** Simulated flow cytometry data set of the bin that lies at the transfection reference protein's global mean (transfection reference: SBFP2) at different gene expression noise levels *σ* (columns) ranging from 0.00–0.32. The plots show the input/output curves extracted by PFAFF (colored crosses; top row: Cerulean, bottom row: Citrine) atop of the simulated stably integrated circuit at the indicated gene expression noise level *σ* (grey density plot). FO undergoes an activation in both output colors. The RIFFM circuit shows a bi-modal behavior already at low noise levels for both output colors.

We applied our data generation tool for transient transfections to FO and RIFFM circuit architectures and simulated 500,000 cells at twelve different Doxycycline input concentrations and six noise levels of *σ*. In Fig 5A we show representative examples of the raw data from transient circuit simulations at a single noise level (*σ* = 0.16) and various Doxycyline modulations. Note the shift in the scatter plots in response to Doxycycline increase. For each noise level, we extract the corresponding input/output relations of the data set with our analysis strategy that we call **P**eak**F**inder **A**nalysis **F**or **F**low cytometry, or PFAFF. The algorithm bins simulated cells according to the expression level of the transfection reference protein SBFP2 each bin containing an equal number of cells (9.5% of total population, 10 bins in total). Next, we determine the modes of log-transformed input and output protein distributions of cells residing in each bin for the different Doxycycline levels as described above, and build the input/output relationships corresponding to that bin (S14–S19 Figs). In the stable integration scenario, we build datasets that correspond to different fixed sets of gene copy numbers. Specifically, the copy numbers are set to correspond to the median copy numbers of the bins used to process the simulated transient transfection data (Methods). We simulate 5,000 cells per Doxycycline value and repeat this for twelve different Doxycycline values to cover the entire input range. This simulation is repeated for each *σ*. These data serve as the gold standard to evaluate the performance of our method for transient transfection data processing, by how well the input/output relationships match the stable integration simulation for matching bin/stable copy number.

In Fig 5B we show the stable integration simulation and the analysis results from PFAFF, the latter extracted from a transfection reference bin that lies close to the global mean of the reference protein (i.e. SBFP2 bin 5; see S14–S19 Figs for all bins, input and noise levels); the former simulated for the gene copy number that corresponds to this reference protein level. Note that the number of transfection reference bins does not influence the outcome (S20 Fig). We plot the stable integration outputs at various input levels as density plots in the background (grayscale). For the lowest noise case (*σ* = 0.00), the density plots for the stable integration collapse to curves, as expected. Gradually increasing *σ* leads to increasingly diffuse input/output relationships. Atop these density plots we superimpose the mode values extracted by PFAFF from the corresponding simulated transient transfection data and binned for the cells that express the same level of the transfection marker as the stable integration. The analysis suggests that the input/output relationship extracted using PFAFF superimposes with the input/output “cloud” simulated for the stable integration, when both reflect similar underlying absolute gene copy number.

**Fig 6:**
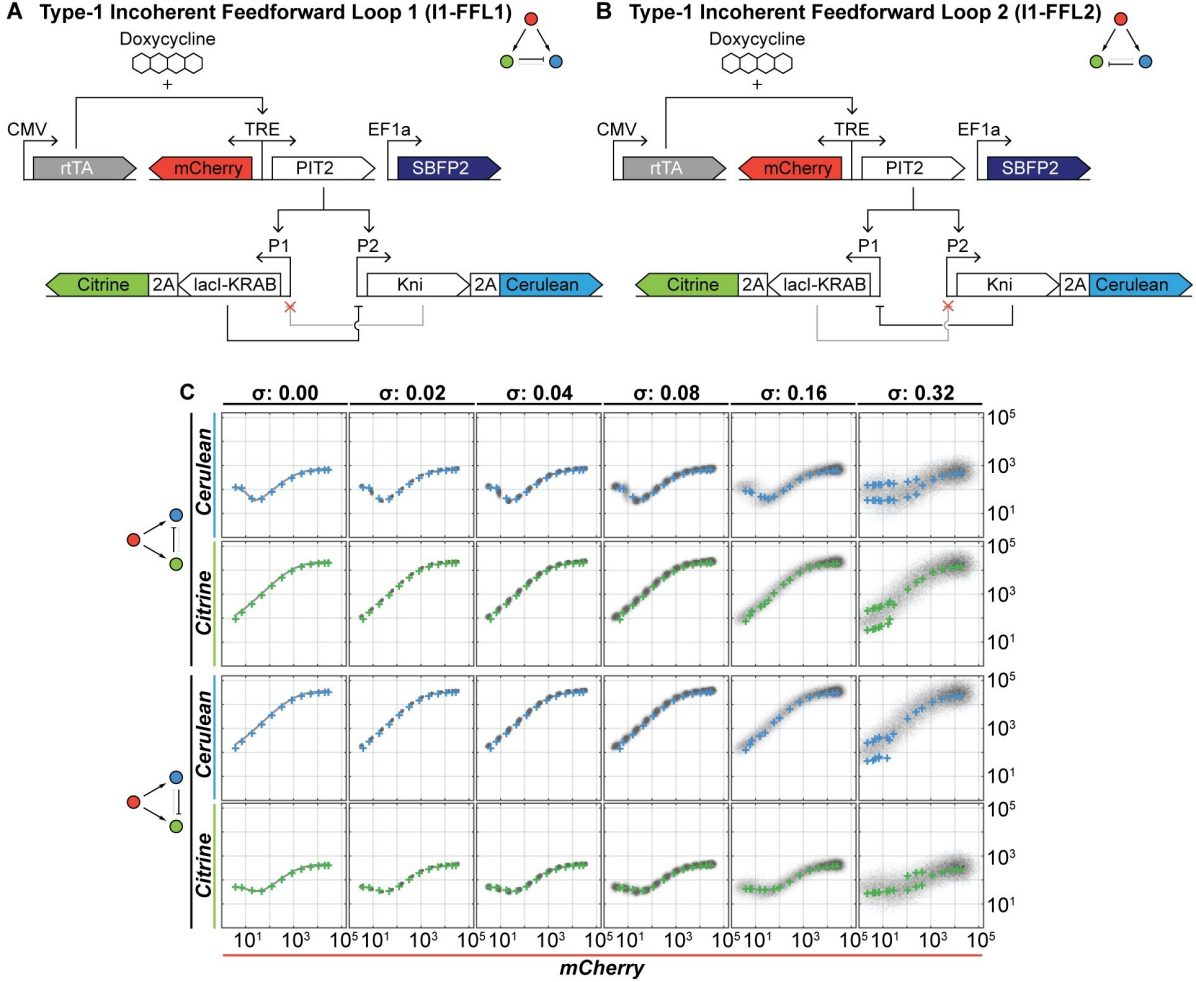
Results of PFAFF analysis on simulated I1-FFLs flow cytometry data sets. **(A)** and **(B)** Circuit architecture of two additional I1-FFLs. **(C)** Simulated flow cytometry data set of the bin that lies at the transfection reference protein's global mean (transfection reference: SBFP2) at different gene expression noise levels *σ* (columns) ranging from 0.00–0.32 is processed by PFAFF. The plots show the input/output curves extracted by PFAFF (colored crosses; top row: Cerulean, bottom row: Citrine) atop of the simulated stably integrated circuit at the indicated gene expression noise level *σ* (grey density plot). Both I1-FFLs show adaptive behaviors in their respective outputs.

In order to expand the number of circuits for analyses, we simulated another commonly studied circuit family–the type-1 incoherent feed-forward motifs. Our simulations include two versions of the I1-FFL (one for each repressor; I1-FFL1 and I1-FFL2; Fig 6A and 6B). We applied the same analyses as before to both I1-FFLs and show the comparison of input/output from stable integrated and transiently transfected circuits (Fig 6C; see S21–S26 Figs for all bins, inputs and noise levels). As is the case for other two circuits, there is excellent correspondence between the input/output curves extracted from simulated transient transfection data, and the input/output behavior of the comparable simulated stable case. This motivated us to expand the number of circuits by a coherent feed-forward, a negative feedback and lastly a positive feedback motif; all of them showing similar, excellent agreement (S27–S29 Figs).

**Fig 7:**
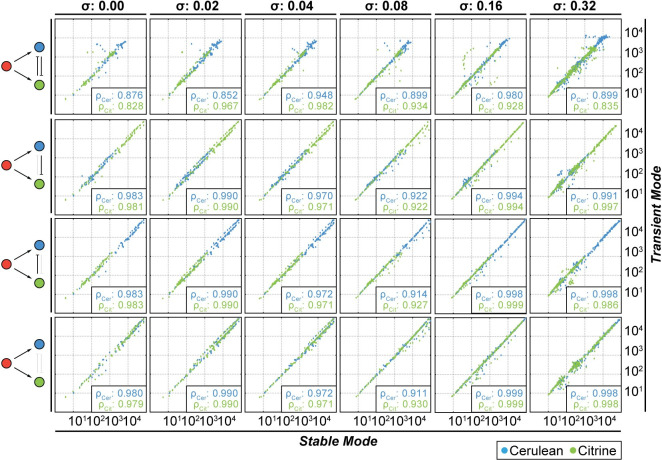
Quantitative analysis of the stable integration and transient transfection data sets. Correlation between extracted output modes of stable integration simulation and PFAFF results from transient transfection simulations. The mode values for both output colors (Cerulean and Citrine) for all input modulator levels and for all bins/stable copy number sets are plotted, and Pearson correlation coefficient (*ρ*_*Cer*_ or *ρ*_*Cit*_) is shown for each plot.

The initial qualitative analysis uncovers excellent overlap between the input/output relationships found by PFAFF, and the input/output clouds from the corresponding stable integration simulations. Only at the highest simulated levels of *σ*, i.e. 0.32, the PFAFF algorithm has minor difficulties with extracting expected input/output relationships. Indeed, a *σ* of 0.32 is much larger than variations observed typically in nature [9,45]. To obtain a quantitative measure of the correspondence, we extracted modes from the log-transformed protein expression distributions of input (mCherry) and outputs (Cerulean and Citrine) from the stable integration data sets with the same peak finder algorithm that we employ in PFAFF (Methods). We correlated the obtained modes with the modes that were found by PFAFF in the transient transfection case (Fig 7). In the pooled modes from all data sets, meaning all external input levels and bins for each noise level, we find a high correlation between the modes from both simulation scenarios for all expression noise levels (mean of Pearson correlation coefficient *ρ*>0.91±0.01 for output modes; *σ*: 0.00–0.32).

## Discussion

In this study we show that transient transfection data can be used to extract input/output relationships of gene circuits that are comparable to the data that would have been obtained with stably-integrated circuits. Our findings reveal that it is sufficient to focus on small subsets of transiently transfected cells that lie at multivariate modes of input and output expression, post-binning on a transfection reference protein, otherwise known as a "transfection marker". We prove numerically that cells in these modes harbor distributions of circuit genes with the following properties: (1) the modes’ absolute values can be deterministically deduced from the observed level of the transfection reference protein expression, the noise level in the experimental data, and the distance from the global mean of the transfection reference protein; (2) the modes’ ratios are identical to the gene or plasmid ratios used in the transient transfection experiments, for all values of the reference protein expression. Moreover, in the case of multimodal data, large differences in protein expression do not translate into significant differences between the underlying gene copy number distributions, and for all practical purposes the absolute and relative copy numbers can be considered identical for the different protein expression modes. Interestingly, the cells that belong to the bin that lies close to the global mean of the reference protein expression, harbor gene copy numbers whose modes' absolute **and** relative values correspond exactly to what one would expect from the naive expectation, namely the ratio between the knowable expressed protein level and the knowable global coefficient of proportionality between the protein and gene copy number. The detailed understanding of the gene copy number behavior in the multivariate protein expression modes that can be identified in the experimental data, provides a degree of confidence in relevance of extracted data to the ground truth behavior of the same circuit when stably integrated in a cell. This confidence is confirmed by the direct *in-silico* validation experiments, where both types of data are directly simulated, the PFAFF workflow analysis is applied to the data from simulated transient transfections, and the results are compared to, and are shown to reproduce, the ground truth.

While transient transfections are often valued as a tool to rapidly analyze genetic circuit behavior, they are rarely used to draw fine-tuned conclusions about the input/output relationship of corresponding stably integrated circuits, more so for multimodal circuits. This is likely due to various pitfalls in existing analysis methods, the most prominent being the insufficient treatment of multimodal systems and the lack of conclusive analysis of the underlying gene copy number distributions in identifiable cell populations. Our analysis strategy allows a thorough comparison of input/output relationships from both scenarios and results in an excellent agreement between them. This will play an important role in gene circuit design and characterization, as it alleviates the need to generate multiple stable cell lines.

## Materials and methods

### Cloning

Standard cloning techniques were used to clone all plasmids. We used *E*. *coli* DH5a and DH10B as the cloning strains, cultured in LB Broth Miller Difco (BD; Cat. no. 244610) and Ampicillin (100ug/ml, Sigma-Aldrich; Cat. no. A0166-5G) as selection medium.

### Cell culture and reagents

All experiments were done with HEK 293 cells (Life Technologies) and were grown at 37°C, 5% CO_2_ in complete medium (DMEM (Thermo Fischer; Cat no. 11965092) supplemented with 10% fetal bovine serum (FBS; Sigma-Aldrich; Cat. no. F9665) and 1% Penicillin/Streptomycin (Sigma-Aldrich; Cat. no. P4333)). They were sub-cultured by seeding 10^6^ cells into T75 flasks every 3–4 days.

### Transfection

One day prior transfection, cells were passed through a 40um cell strainer (Falcon; Cat. No 352340) and counted with Bio Rad TC10. In each well (uncoated 6-well plates, Thermo Scientific Nunc; Cat. No. 2020–10) 300,000 cells were seeded and incubated for another 24 hours. On the day of transfection DNA was diluted in 250ul Opti-MEM I Reduced Serum (Gibco, Life Technologies Cat no. 31985–962) and mixed with a 244ul Opti-MEM I/6ul Lipofectamin 2000 Transfection Reagent (Thermo Fischer; Cat. no. 11668019). After a 20 minutes’ incubation step at room temperature, the transfection mix was added drop wise to the wells. The cells were incubated for another 72 hours before being measured by flow cytometry.

### Flow cytometry

All samples were measured with a BD LSR Fortessa cell analyzer. The medium was removed and cells were incubated with 300ul StemPro Accutase Cell Dissociation Reagent (Thermo Fischer; Cat. no. A1110501) at 37°C, 5% CO_2_ for 10 minutes. Reporter specific combinations were used to measure all four fluorescent proteins independently, but still providing a setup of little bleed over. In particular, we used for: SBFP2 a 405nm laser with 445/15, Cerulean 445nm laser with 473/10, Citrine 488nm laser with 542/27, mCherry 561nm laser with 610/20 and iRFP 640nm laser with 710/50 emission filter sets. We used the same PMTs (FSC: 350, SSC: 350, SBFP2: 220, Cerulean: 242, Citrine: 220, mCherry: 245, iRFP: 460) throughout all measurements and controlled for consistency of the instrument by using SPHERO RainBow Calibration particles (Cat no. 559123, BD).

### Co-transfection experiment

We experimentally co-transfected five different fluorescent protein genes (SBFP2 [46], Cerulean [47], Citrine [48], mCherry [49], iRFP [50]; S30 Fig), individually driven by an Ef1a promoter and analyzed them via flow cytometry. The amount of transfected DNA (ng) was adjusted according to each plasmid’s size (nominal ratio: SBFP2: Cerulean: Citrine: mCherry: iRFP = 504: 634: 400: 239: 249). We collected more than 1,000,000 events and stringently gated the live population (~750,000 cells). This experiment created a five-dimensional distribution of fluorescent values.

### Fan-out gene circuit experiment

We transfected five gene cassettes on individual plasmids as depicted in Fig 2A into HEK293 cells and activated the circuit through the addition of Doxycycline at eight different input modulation levels (0nM, 0.90nM, 3.15nM, 0.01uM, 0.05uM, 0.13uM, 0.45uM and 1.35uM). After 72h post-transfection we analyzed the induced cells using flow cytometry and collected more than 1,000,000 events per replicate (*n* = 3). The obtained data were subjected to our analysis pipeline as outlined in section Data Analysis.

### Model

#### Co-transfection model

We generated the model of multiple constitutively expressed genes using a steady state approximation (Eqs 7 and 21). Once the gene copy number *k*_*i*_ and expression parameter *β*_*i*_ are determined, the protein output *O*_*i*_ is computed as described in S1 Fig.

#### Circuit models

ODE circuit models were created with Simbiology, a MathWorks MATLAB 2018b package. Each molecular interaction was modeled according to the law of mass action (S3 Text "Detailed Models" and parameter values in S2 Table). This includes binding and unbinding of a transcription factor to inducible promoters, transcription of mRNAs and translation into proteins. All circuits have the same underlying interaction map. We created four different topologies by inactivating the translation reaction of the respective repressor mRNAs. Therefore, we generated *in silico* "knock outs" with minimal changes to the model.

### Parameters

#### Expression rate parameters

The vector of global proportionality coefficients ***β*** used in the simulation of constitutive gene co-transfection is a measure for the conversion of gene copy numbers to the number of proteins. In our circuit models, this coefficient is derived from expression and degradation rate constants. We adjusted the values of *β*_*i*_ according to the maximum expression levels of our circuit models to obtain similar and biologically feasible amounts (S1 Table).

In dynamic circuit models, binding/unbinding and transcription rates were either fitted from experimental data (Manuscript in preparation), literature values, or set arbitrarily at biologically feasible values. The translation rates *π*_*i*_ are based on previously-reported values [51] and were adjusted according to the length of each protein (S3 Text "Detailed Models").

#### Anticipated gene copy number $k_{i}^{*}$

In order to compute the values of anticipated gene copy numbers $k_{i}^{*}$ from Eq 27, we first have to determine the *Y*_1_ values. This is done by removing the top and bottom 0.1 percentile from the *in-silico* co-transfection and circuit simulations and binning the obtained data set into equally spaced bins (50 bins for co-transfection simulations; 25 bins for circuit simulations) according to the signal intensity of the transfection marker (*O*_1_). Since the *O*_1_ distribution within a bin is just a subset of the global *O*_1_ distribution, we use the median of the log-transformed *O*_1_ signal for each bin, which is then identified as *Y*_1_ value. Together with the global proportionality coefficient *β*_1_ (see above) and the abundance parameter *a*_*i*_ we can compute $k_{i}^{*}$ according to Eq 27 for each bin of the respective data set.

### In-silico flow cytometry simulations

#### Gene expression noise from lognormal distributions

Within our *in-silico* simulations, we introduce gene expression noise through the randomization of kinetic parameters. In the case of co-transfection simulations we randomize the vector of the proportionality coefficients ***b*** by drawing its individual values from a lognormal distribution $LN(\mu_{\beta },\sigma )$, with the mean *μ*_*β*_ being their coefficient of proportionality of the respective gene *g*_*i*_ (S1 Table) in log-space $\left(\mu_{\beta }=\ln\left(\beta_{i}\right)-\frac{\sigma^{2}}{2}\right)$ and the standard deviation *σ* being one of the six noise levels (0.00, 0.02, 0.04, 0.08, 0.16 or 0.32). In the case of circuit simulations, we introduce variability through the translation parameter vector ***p***. Likewise, it is drawn from a lognormal distribution $LN(\mu_{\pi },\sigma )$ with the mean being set according to S2 Table in log-space $\left(\mu_{\pi }=\ln\left(\pi_{i}\right)-\frac{\sigma^{2}}{2}\right)$ and the standard deviation being again one of the six noise levels.

#### Gene expression noise from *Γ* distributions

Gene expression noise is introduced by drawing individual values ***b*** of the proportionality coefficients from a *Γ* (gamma) distribution, *Γ*(*k*,*θ*). We chose the shape parameter *k* and the scaling parameter *θ*, so that the mean (*β*_*i*_) and the variance of the distribution are the same as in the lognormal case. We simulated transient co-transfections at *σ* = 0.08.

#### Gene copy number/extrinsic noise

We introduce extrinsic noise in our transient transfection simulations by drawing gene copy numbers ***k*** from a five-dimensional multivariate normal distribution $N_{N}(\mu ,\Sigma )$. The mean of the distribution, ***μ*** = ***a****m*, depends on the multiplicity parameter *m*, which is drawn from a lognormal distribution $LN(\mu_{m},\sigma_{m})(\mu_{m}=1.4979,\;\sigma_{m}=1.2686$), and the abundance vector ***a*** (equimolar: *a*_1_:*a*_2_:*a*_3_:*a*_4_:*a*_5_ = 1.0: 1.0: 1.0: 1.0: 1.0 or nominal: *a*_1_:*a*_2_:*a*_3_:*a*_4_:*a*_5_ = 1.0: 1.3: 0.8: 0.5: 0.4). For our systematic comparison of gene copy number distributions (S5 Fig), we draw the multiplicity parameter *m* from a Poisson (*Pois*(*λ*), *λ* = 10) and *Γ* distribution (*Γ*(*k*,*θ*), *k* = 0.7436, *θ* = 13.46), respectively. The covariance **Σ** = *diag*((***a****mε*)^2^) is diagonal matrix and depends on the abundance ***a***, the multiplicity *m* and the constant factor *ε* = 0.04. For every simulated cell, the multivariate distribution $N_{N}(\mu ,\Sigma )$ changes along the lognormal distribution$LN(\mu_{m},\sigma_{m})$ and in every iteration five gene copy numbers are drawn from it.

#### Single-plasmid simulations

The single-plasmid circuit contains all gene cassettes on a single entity. We achieve this by setting the constant factor *ε* to zero. Consequently, the covariance matrix of the five-dimensional multivariate distributions **Σ** also turns zero. The remaining simulation is performed as described below in section “Gene circuit simulations”.

#### Co-transfection simulations

We simulated our simple model independently 5×10^6^ times (*C*), randomizing both the parameters ***b*** and gene copy number ***k***, according to the description above. Each simulated run corresponds to a single cell and contains a randomized set of ***b*** and ***k***. We simulated the steady-state and the runs were stored in a single .*csv*-file that contained all information used to generate the data afterwards. This includes the individual parameters ***b*** and ***k*** for every cell as well as the output values. Thus, we obtained a data set that resembles a transient co-transfection experiment aided by the information of individual parameters. The simulations were performed in MATLAB 2018b.

1: program: Simulation Transient Co-transfection

2:    initialize abundance ***a***, constant factor *σ*_*e*_ = 0.04

3:    for Noise-Level *σ* in [0.00, 0.02, 0.04, 0.08, 0.16, 0.32]

4:        for Cell *j* in 1:*C*

5:            draw *m*_*j*_ from $LN(\mu_{m},\sigma_{m})$, *μ*_*m*_ = 1.4979 and *σ*_*m*_ = 1.2686

6:            seed and draw ***k***_***j***_ from $N_{N}(\mu ,\Sigma ),$ ***μ*** = ***a****m*_*j*_, **Σ** = *diag*((***a****m*_*j*_*σ*_*e*_)^2^)

7:            draw ***b***_***j***_ from $LN(\mu_{\beta },\sigma )$, $\mu_{\beta }=\ln\left(\beta \right)-\frac{\sigma^{2}}{2}$

8:            Model: ***O***_***j***_ = ***k***_*j*_***b***_***j***_

9:        **end**

10:    **end**

11:    **save** Simulation.csv

12: **end program**

#### Gene circuit simulations

*In-silico* simulations of flow cytometry data for our circuits requires a mathematical model (ODE) generated by MATLAB’s Simbiology toolbox. The model was exported into the workspace and to decrease computational effort, we generated a SimFunction object. This function has five outputs: the number of fluorescent proteins (SBFP2, Cerulean, Citrine, mCherry) and transcription factor rtTA bound to Doxycycline at steady state (i.e. 1,500,000s). As the SimFunction’s input serves a matrix wherein each column represents the gene copy number (*k*_1_: pCS187, *k*_2_: pCS171, *k*_3_: pCS166, *k*_4_: pCS200, *k*_5_: pZ91), the Doxycycline level ***DOX*** (*Z* = 12 logarithmically spaced values from 10–500,000 molecules) and eight translation parameters ***p***, each for every protein ***O*** produced. Sets of gene copy numbers ***k***_***j***_ and gene expression noise variations ***p***_***j***_ were drawn as described above. The simulation input, output as well as all parameters used for each cell are stored in a table and saved as.*csv*-files for documentation and further analysis. The simulations were performed in MATLAB 2018b.

1: program: Simulation Transient Circuits

2:    initialize abundance ***a***, constant factor *σ*_*e*_ = 0.04

3:        for Noise-Level *σ* in [0.00, 0.02, 0.04, 0.08, 0.16, 0.32]

4:            for Input-Level *l* in 1: *Z*

5:                for Cell *j* in 1: *n*

6:                    draw *m*_*ij*_ from $LN(\mu_{m},\sigma_{m})$, *μ*_*m*_ = 1.4979 and *σ*_*m*_ = 1.2686

7:                    seed and draw ***k***_***lj***_ from $N_{N}(\mu ,\Sigma ),\;\mu =am_{lj},\;\Sigma =diag{\left((am_{lj}\sigma_{e}\right)}^{2})$

8:                    draw ***p***_***lj***_ from $LN(\mu_{\pi },\sigma ),\;\mu_{\pi }=\ln\left(\pi \right)-\frac{\sigma^{2}}{2}$

9:                    initialize and simulate SimFunction-Model

10:                **end**

11:            **end**

12:        **end**

13:    **save** Simulation.csv

14: **end program**

#### Determine copy numbers for stably integrated gene circuits

We first simulated the transient transfection data set according to our initial parameters, which we drew from previous experiences. After binning the data set according to the protein output from our transfection marker *O*_1_, we determined the mode of the gene copy number *k*_1_ within each bin. The other values are derived from *k*_1_ according to their abundance coefficients. These values serve as the gene copy number for stably integrated gene circuits.

### Data analysis

#### Pre-processing of experimental flow cytometry data

Retrieved data from BD LSR Fortessa was recorded with BD FACS Diva Software. The resulting files were exported in .*fcs* format and loaded into FlowJo software [52]. There, compensation of individual fluorescent channels was performed, live population gated and exported as scaled values into .*csv*-files.

#### Bi-exponential transformation

Scaled FACS values were transformed into bi-exponential space when needed via formulas from Parks et al. [53] with parameters *M* = 4.5, *p* = 2, *T* = 262144 and *W* = 0.401:

$$S(X;W)=T*10^{-\Delta }(10^{\Delta }-p^{2}*10^{-\frac{\Delta }{p}}+p^{2}-1)$$

where Δ = *X*−*W for X*≥*W* and Δ = *W*−*X else*.

#### Gene copy number distributions *k*_*i*_ in output peaks

After binning the data set according to the transfection marker (50 bins in case of the co-transfection simulations, 25 bins in case of the circuit simulations), we fit Gaussians to the log-transformed values. We slice a window of ±0.15 log_10_ units around the mode(s) of the fitted distribution. Within this narrow window, we repeat the process for the remaining genes (co-transfection case: 1. Cerulean, 2. Citrine, 3. mCherry, 4. iRFP; circuit case: 1. mCherry, 2. Cerulean or Citrine). Since all parameters needed for the simulations are stored in an array, we can select all cells within that final slice and look up the gene copy numbers that were used to generate this subset of output data. The distributions of the gene copy numbers are then processed to discover their modes.

#### Peak Finder Algorithm for Flow cytometry (PFAFF)

The software is available on GitHub (https://github.com/benensonlab/PFAFF). The repository contains the code, detailed S4 Text "PFAFF User Manual", S5 Text "Description of the example data set" and sample simulated data for running the analysis. User-provided data can also be analyzed according to the steps described in User Manual.

The algorithm’s procedure starts by discarding the tails of the transfection control’s distribution. Within this window (i.e. 2.5–97.5% of transfection control fluorescence intensity) the distribution is segmented into bins of equal number of events (i.e. ten bins). Each bin is analyzed sequentially and all values are transformed bi-exponentially. The input distribution (i.e. mCherry) is approximated by a histogram in bi-exponential space and Gaussians are fitted to it. A following set of rules determines the number of fitted Gaussians:

1: **program** Fit Gaussians to mCherry Distribution

2:        **if** Goodness-of-Fit for one Gaussian **>** 0.975 **then**

3:          **save** mode value

4:          exit

5:        else

6:          fit two Gaussians

7:          **if** Goodness-of-Fit for two Gaussians **>** 0.99 **then**

8:          **save** mode values

9:          else

10:          fit three Gaussians

11:          **if** distance between two peaks **<** 0.42 **then**

12:          go back to use two Gaussian fit

13:          exit

14:          **elseif** distance (mean closest-modes) to (two-Gaussian-Fit modes) < 0.3

15:          **save** two-Gaussian-Fit mode and remaining three-Gaussian-Fit mode

16:          end if

17:          **save** mode values

18:          end if

19:          **if** distance between the two modes **<** 0.75 **then**

20:          go back to use one Gaussian fit

21:          exit

22:          end if

23:        end if

24: end program

A window of ±0.1 bi-exponential units is sliced around the peaks’ center. Within that subset of cells, distributions of the output colors (i.e. Cerulean and Citrine) are again approximated by histograms. Much like before a set of rules determines the number of Gaussians that are fitted to these distributions:

1: **program** Fit Gaussians to Cerulean or Citrine Distribution

2:        **if** Goodness-of-Fit for one Gaussian **>** 0.975 **then**

3:          **save** mode value

4:          exit

5:        else

6:          fit two Gaussians

7:          **if** Goodness-of-Fit for two Gaussians **>** 0.995 **then**

8:          **save** mode values

9:          else

10:          fit three Gaussians

11:          **if** distance between peaks with highest intensities **<** 0.9 **then**

12:          remove remaining peak from the data set

13:          fit one Gaussian for the highest peak

14:          **if** Goodness-of-Fit **>** = Goodness-of-Fit for two Gaussians **then**

15:          **save** mode values

16:          else

17:          **save** mode values of two Gaussian-Fit

18:          end if

19:          **if** distance between the two modes **<** 0.75 **then**

20:          go back to use one Gaussian fit

21:          exit

22:          end if

23:        end if

24: end program

For each bin, we repeat this fitting procedure. All extracted modes are re-transformed into flow cytometry units and stored in a table. The output of this algorithm is saved as MATLAB workspaces, that contain variables for generating (weighted) input/output mappings. Furthermore, various plots are generated (density plots of (raw) data, individual fits to data distributions, weighted input/output mappings and weighted mean input/output mappings) and saved as individual files in the result folder (see provided manual for details).

## Supporting information

S1 Text In-silico time-courses. (DOCX)

S2 Text Simple Fan-Out Model. (DOCX)

S3 Text Detailed Models. (DOCX)

S4 Text PFAFF User Manual. (DOCX)

S5 Text Description of the example data set. (DOCX)

S1 Fig Parameter drawing workflow. (TIF)

S2 Fig Co-transfection experiment and in silico simulations. (TIF)

S3 Fig In-silico simulations of a transient co-transfection with equimolar plasmid ratio. (TIF)

S4 Fig In-silico simulations of a transient co-transfection with nominal plasmid ratio. (TIF)

S5 Fig In-silico simulation of transient co-transfections at various initial gene copy number and parameter distributions. (TIF)

S6 Fig PFAFF applied to experimental data of a FO circuit. (TIF)

S7 Fig Comparison of multi-plasmid and single-plasmid gene circuits. (TIF)

S8 Fig In-silico simulations of a transiently transfected monomodal circuit. (TIF)

S9 Fig In-silico simulations of a transiently transfected bi-modal circuit. (TIF)

S10 Fig Gene copy number ratios from the simulations of transiently transfected bi-modal circuit. (TIF)

S11 Fig In silico simulations of stable integrations and transient transfections. (TIF)

S12 Fig Workflow for simulation and analysis of genetic circuits. (TIF)

S13 Fig Concatenation of high and low input and output modes. (TIF)

S14 Fig In-silico simulation of stably integrated and transiently transfected (PFAFF input/output) circuits (RIFFM and FO) at intrinsic noise level 0.00. (TIF)

S15 Fig In-silico simulation of stably integrated and transiently transfected (PFAFF input/output) circuits (RIFFM and FO) at intrinsic noise level 0.02. (TIF)

S16 Fig In-silico simulation of stably integrated and transiently transfected (PFAFF input/output) circuits (RIFFM and FO) at intrinsic noise level 0.04. (TIF)

S17 Fig In-silico simulation of stably integrated and transiently transfected (PFAFF input/output) circuits (RIFFM and FO) at intrinsic noise level 0.08. (TIF)

S18 Fig In-silico simulation of stably integrated and transiently transfected (PFAFF input/output) circuits (RIFFM and FO) at intrinsic noise level 0.16. (TIF)

S19 Fig In-silico simulation of stably integrated and transiently transfected (PFAFF input/output) circuits (RIFFM and FO) at intrinsic noise level 0.32. (TIF)

S20 Fig PFAFF output for various bin numbers. (TIF)

S21 Fig In-silico simulation of stably integrated and transiently transfected (PFAFF input/output) circuits (I1-FFL1 and I1-FFL2) at intrinsic noise level 0.00. (TIF)

S22 Fig In-silico simulation of stably integrated and transiently transfected (PFAFF input/output) circuits (I1-FFL1 and I1-FFL2) at intrinsic noise level 0.02. (TIF)

S23 Fig In-silico simulation of stably integrated and transiently transfected (PFAFF input/output) circuits (I1-FFL1 and I1-FFL2) at intrinsic noise level 0.04. (TIF)

S24 Fig In-silico simulation of stably integrated and transiently transfected (PFAFF input/output) circuits (I1-FFL1 and I1-FFL2) at intrinsic noise level 0.08. (TIF)

S25 Fig In-silico simulation of stably integrated and transiently transfected (PFAFF input/output) circuits (I1-FFL1 and I1-FFL2) at intrinsic noise level 0.16. (TIF)

S26 Fig In-silico simulation of stably integrated and transiently transfected (PFAFF input/output) circuits (I1-FFL1 and I1-FFL2) at intrinsic noise level 0.32. (TIF)

S27 Fig Results of PFAFF analysis on simulated cFFL flow cytometry data sets. (TIF)

S28 Fig Results of PFAFF analysis on simulated negFB flow cytometry data sets. (TIF)

S29 Fig Results of PFAFF analysis on simulated posFB flow cytometry data sets. (TIF)

S30 Fig Maps of plasmids used in co-transfection experiment. (TIF)

S31 Fig (Quasi) steady states of a fan-out circuit at different protein degradation rates. (TIF)

S1 Table Model parameter values for co-transfection simulations. (DOCX)

S2 Table Model parameter values for tested circuit architectures (RIFFM, I1-FFL1/2, FO, cFFL, negFB, posFB). (DOCX)

S3 Table List of plasmids. (DOCX)

S4 Table List of primers. (DOCX)

S5 Table Model parameter values for simple fan-out circuit. (DOCX)
