# Supplementary material for: Precise determination of input-output mapping for multimodal gene circuits using data from transient transfection
Source: PLoS Comput Biol. 2020 Nov 30;16(11):e1008389. doi: 10.1371/journal.pcbi.1008389 (PMC7728399; doi:10.1371/journal.pcbi.1008389)
Supplement: S1 Text — (DOCX) [file pcbi.1008389.s001.docx]

## S1 Text

## *In-silico* time-courses

In order to understand the effects of short protein half-life times (or alternatively cell divisions and thus gene/plasmid dilutions in the transient transfection scenario) during our *in-silico* simulations, we set out to simulate a transiently-transfected gene circuit. For this purpose, we used a simplified version of the fan-out circuit consisting of a reduced set of ordinary differential equations (ODE), supplemented with reactions for protein degradation and gene dilution (see SI section Simple Fan-Out Model and table S5). We simulated the model as described in the Materials and Methods section earlier. All simulations were performed at gene expression noise $\sigma=0.16$ and repeated for 10,000 cells at ten input modulation levels ($\beta_{g_{2}}$: 0.0001, 0.0002, 0.0005, 0.0012, 0.0029, 0.0068, 0.0158, 0.0369, 0.0859 and 0.2000 [molecules/s]). To account for cell division, we set the gene dilution half-life time $\tau_{DNA}$ to 48h (cell division or gene dilution rate $\delta_{DNA}=\frac{\ln\left( 2 \right)}{3600\cdot\tau_{DNA}}$ in [1/s]) and set the global protein half-life time parameter $\tau_{p}$ to either 1.5h, 3h, 6h, 12h 24h or 48h (protein degradation rate $\delta_{P}=\frac{\ln\left( 2 \right)}{3600\cdot\tau_{p}}$ in [1/s]). As ground truth we used the same model/parameter setup, but set the dilution rate parameter $\delta_{DNA}\equiv0$ [1/s] (**no dilution**).

We ran the model to steady-state for the scenario without gene dilution ($\delta_{DNA}=0$ [1/s]; **no dilution**) and to quasi steady-state in scenarios including gene dilution ($\delta_{DNA}\neq0$ [1/s]). As quasi steady-state we considered (i) the timepoint at the **peak** value of protein expression (protein half-life time of 1.5h: 26,666 sec; 3h: 43,333 sec; 6h: 70,000 sec; 12h: 100,000 sec; 24h: 136,666 sec; 48h: 173,333 sec) or (ii) a random timepoint picked from an **interval** around the peak protein expression (1.5h: [30,000 sec -50,000 sec]; 3h: [40,000 sec -70,000 sec]; 6h: [70,000 sec -100,000 sec]; 12h: [100,000s-140,000 sec]; 24h: [140,000 sec -190,000 sec]; 48h: [170,000 sec -200,000 sec]). The latter method reflects the reality of a snapshot flow cytometry measurement, where is it difficult to capture every cell at the peak of its output.

The raw input ($O_{2}$)/output ($O_{3}$, $O_{4}$) density plots are qualitatively alike in all three cases (**no dilution**, timepoint at **peak** value and timepoint picked from an **interval**; S31A Fig). The data sets were analyzed using PFAFF and revealed that the absolute output levels decrease with a decrease in protein half-life time (S31B Fig), as expected. There is almost no difference between the two methods of output readout (**peak** vs **interval**), highlighting the fact that snapshot readout should provide data that it close to the ideal case. The comparison to the **no dilution** case shows the expected result, in that the I/O behavior is virtually not affected by DNA dilution when protein half life time is much shorter than that of DNA (*i.e.*, much shorter than cell generation time); and on the other hand, the absolute output values get reduced when the protein and the DNA half life time are of the same order of magnitude. This reduction is a consequence of the decrease in the apparent protein half life time due to cell division. That said, the qualitative nature of the I/O relationship does not change. The practical conclusion of this simulation is that it is preferable to design circuits in which protein half life time is substantially shorter than the cell division time, and if possible, use cells that are almost quiescent, as long that those can be transiently transfected.
