# Supplementary material for: Precise determination of input-output mapping for multimodal gene circuits using data from transient transfection
Source: PLoS Comput Biol. 2020 Nov 30;16(11):e1008389. doi: 10.1371/journal.pcbi.1008389 (PMC7728399; doi:10.1371/journal.pcbi.1008389)
Supplement: S2 Text — (DOCX) [file pcbi.1008389.s002.docx]

### S2 Text

### Simple Fan-Out Model

$$\dot{g_{1}}=-\delta_{DNA}\cdot g_{1}$$

$$\dot{g_{2}}=-\delta_{DNA}\cdot g_{2}$$

$$\dot{g_{3}}=-k_{on}^{P_{2}}\cdot P_{2}^{2}\cdot g_{3}+k_{off}\cdot g_{3}\_P_{2}-\delta_{DNA}\cdot g_{3}+\delta_{P}\cdot g_{3}\_P_{2}$$

$$\dot{g_{4}}=-k_{on}^{P_{2}}\cdot P_{2}^{2}\cdot g_{4}+k_{off}\cdot g_{4}\_P_{2}-\delta_{DNA}\cdot g_{4}+\delta_{P}\cdot g_{4}\_P_{2}$$

$$\dot{g_{3}\_P_{2}}=k_{on}^{P_{2}}\cdot P_{2}^{2}\cdot g3-k_{off}\cdot g_{3}\_P_{2}-\delta_{DNA}\cdot g_{3}\_P_{2}-\delta_{P}\cdot g_{3}\_P_{2}$$

$$\dot{g_{4}\_P_{2}}=k_{on}^{P_{2}}\cdot P_{2}^{2}\cdot g_{4}-k_{off}\cdot g_{4}\_P_{2}-\delta_{DNA}\cdot g_{4}\_P_{2}-\delta_{P}\cdot g_{4}\_P_{2}$$

$$\dot{P_{1}}=\beta_{g_{1}}\cdot g_{1}-\delta_{DNA}\cdot P1-\delta_{P}\cdot P1$$

$$\dot{P_{2}}=\beta_{g_{2}}\cdot g_{2}-\delta_{DNA}\cdot P_{2}-\delta_{P}\cdot P2-2\cdot\left( k_{on}^{P_{2}}\cdot P_{2}^{2}\cdot g_{3}-k_{off}\cdot g_{3}\_P_{2} \right)-2\cdot(k_{on}^{P_{2}}\cdot P_{2}^{2}\cdot g_{4}-k_{off}\cdot g_{4}\_P_{2})$$

$$\dot{P_{3}}=\beta_{g_{3}}\cdot g_{3}\_P_{2}-\delta_{DNA}\cdot P_{3}-\delta_{P}\cdot P_{3}$$

$$\dot{P_{4}}=\beta_{g_{4}}\cdot g_{4}\_P_{2}-\delta_{DNA}\cdot P_{4}-\delta_{P}\cdot P_{4}$$
