# Supplementary material for: Precise determination of input-output mapping for multimodal gene circuits using data from transient transfection
Source: PLoS Comput Biol. 2020 Nov 30;16(11):e1008389. doi: 10.1371/journal.pcbi.1008389 (PMC7728399; doi:10.1371/journal.pcbi.1008389)
Supplement: S3 Text — (DOCX) [file pcbi.1008389.s003.docx]

## S3 Text

## Detailed Models

The simulation of our investigated gene circuits requires a mathematical model. We chose to model it with odes that capture the dynamics of the circuits. Each model consists of a basic set of ODEs that are identical in all circuits and ODEs that are specific for each circuit. They contain the translation parameters of the respective repressors and are either turned on or off. In the off state, we achieve a “genetic knock out”. Thus, we are able to model all circuits with only minimal changes in the models. The model parameter values are listed in S2 Table.

### Basic set of ODEs

| 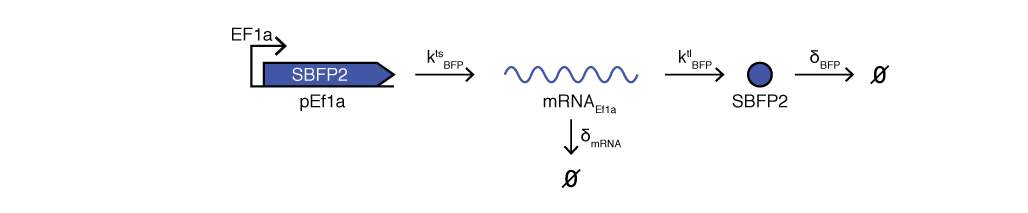  $\dot{pEf1a}=-\delta_{DNA}\cdot pEf1a$  $\dot{mRNA_{Ef1a}}=k_{BFP}^{ts}\cdot pEf1\alpha-\delta_{mRNA}\cdot mRNA_{Ef1a}$  $\dot{BFP}=\pi_{BFP}\cdot{mRNA}_{187}-\delta_{BFP}\cdot BFP$ |
| --- |

| 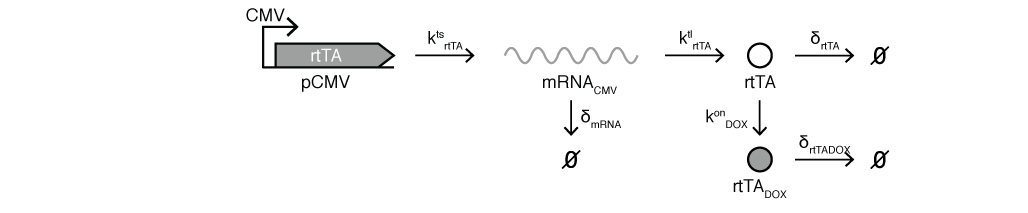$\dot{pCMV}=-\delta_{DNA}\cdot pCMV$  $\dot{mRNA_{CMV}}=k_{rtTA}^{ts}\cdot pCMV-\delta_{mRNA}\cdot mRNA_{CMV}$  $\dot{rtTA}=\pi_{rtTA}\cdot mRNA_{91}-\left( k_{DOX}^{on}\cdot rtTA\cdot DOX-k_{DOX}^{off}\cdot rtTA_{DOX} \right)-\delta_{rtTA}\cdot rtTA$  $\dot{{rtTA}_{DOX}}=\left( k_{DOX}^{on}\cdot rtTA\cdot DOX-k_{DOX}^{off}\cdot rtTA_{DOX} \right)-\left( k_{rtTA_{DOX}}^{on}\cdot pTRE\cdot rtTA_{DOX}-k^{off}\cdot p{TRE}_{rtTA_{DOX}} \right)-\delta_{rtTA_{DOX}}\cdot rtTA_{DOX}$ |
| --- |

| 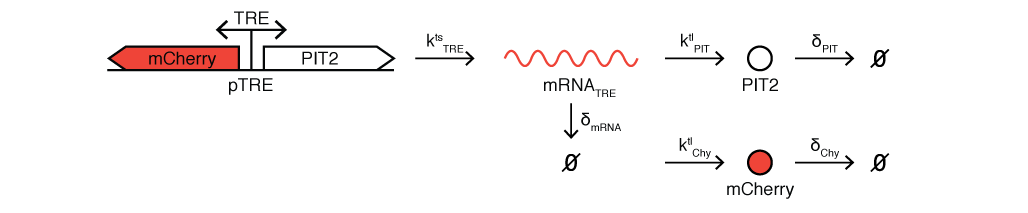  $\dot{\mathrm{DOX}}=-(k_{\mathrm{DOX}}^{\mathrm{on}}\cdot rtTA\cdot DOX-k_{\mathrm{DOX}}^{\mathrm{off}}\cdot rtTA_{\mathrm{DOX}})+ \delta_{\mathrm{DOX}}\cdot DOX$  $\dot{pTRE}=-\left( k_{rtTA_{DOX}}^{on}\cdot pTRE\cdot rtTA_{DOX}-k^{off}\cdot p{TRE}_{rtTA_{DOX}} \right)+\delta_{rtTA_{DOX}}\cdot p{TRE}_{rtTA_{DOX}}-\delta_{DNA}\cdot pTRE$  $\dot{p{TRE}_{DOX}}=\left( k_{rtTA_{DOX}}^{on}\cdot pTRE\cdot rtTA_{DOX}-k^{off}\cdot p{TRE}_{rtTA_{DOX}} \right)-\left( \delta_{rtTA_{DOX}}+\delta_{DNA} \right)\cdot p{TRE}_{rtTA_{DOX}}$  $\dot{mRNA_{TRE}}=k_{TRErtTA_{DOX}}^{ts}\cdot p{TRE}_{rtTA_{DOX}} -\delta_{mRNA}\cdot mRNA_{TRE}$  $\dot{mCherry}=\pi_{Chy}\cdot{mRNA}_{200}-\delta_{Chy}\cdot mCherry$  $\dot{PIT}=\pi_{PIT}\cdot mRNA_{200}-\left( k_{P1_{PIT}}^{on}\cdot PIT\cdot\left( P1+P1_{lac} \right)-k^{off}\cdot\left( P1_{PIT}+P1_{PITlac} \right) \right)-\left( k_{P2_{PIT}}^{on}\cdot PIT\cdot\left( P2+P2_{Kni} \right)-k^{off}\cdot\left( P2_{PIT}+P2_{PITKni} \right) \right)-\delta_{PIT}\cdot PIT$ |
| --- |
| 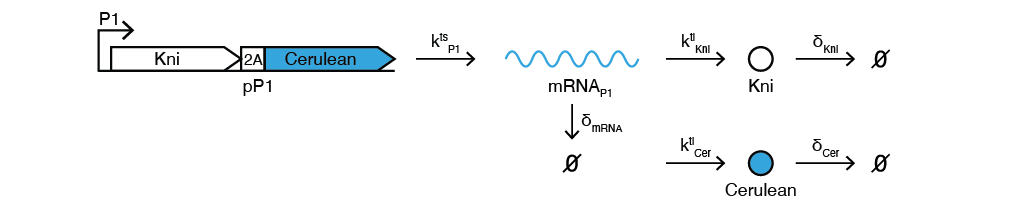  $\dot{Cerulean}=\pi_{Cer}\cdot{mRNA}_{171}-\delta_{Cer}\cdot Cerulean$  $\dot{mRNA_{P1}}=k_{P1}^{ts}\cdot P1 + k_{P1PIT}^{ts}\cdot P1_{PIT}+k_{P1lac}^{ts}\cdot P1_{lac}+k_{P1PITlac}^{ts}\cdot P1_{PITlac}-\delta_{mRNA}\cdot mRNA_{P1}$  $\dot{P1_{lac}}=\left( k_{lac}^{on}\cdot P1\cdot la{cI}^{4}-k^{off}\cdot P1_{lac} \right)-\left( k_{P1_{PIT}}^{on}\cdot P1_{lac}\cdot PIT-k^{off}\cdot P1_{PITlac} \right)-\left( \delta_{lac}+\delta_{DNA} \right)\cdot P1_{lac}$  $\dot{P1_{PITlac}}=\left( k_{lac}^{on}\cdot P1_{PIT}\cdot lacI^{4}-k^{off}\cdot P1_{PITlac} \right)+\left( k_{P1_{PIT}}^{on}\cdot P1_{lac}\cdot PIT-k^{off}\cdot P1_{PITlac} \right)-\left( \delta_{PIT}+\delta_{DNA} \right)\cdot P1_{PITlac}$  $\dot{P1_{PIT}}=\left( k_{P1PIT}^{on}\cdot P1\cdot PIT-k^{off}\cdot P1_{PIT} \right)-\left( k_{lac}^{on}\cdot P1_{PIT}\cdot lacI^{4}-k^{off}\cdot P1_{PITlac} \right)-\left( \delta_{PIT}+\delta_{DNA} \right)\cdot P1_{PIT}$  $\dot{P1}=-\left( k_{P1PIT}^{on}\cdot P1\cdot PIT-k^{off}\cdot P1_{PIT} \right)-\left( k_{lac}^{on}\cdot P1\cdot lacI^{4}-k^{off}\cdot P1_{lac} \right)+\delta_{PIT}\cdot\left( P1_{PIT}+P1_{PITlac} \right)+ \delta_{lac}\cdot P1_{lac}-\delta_{DNA}\cdot P1$ |
| 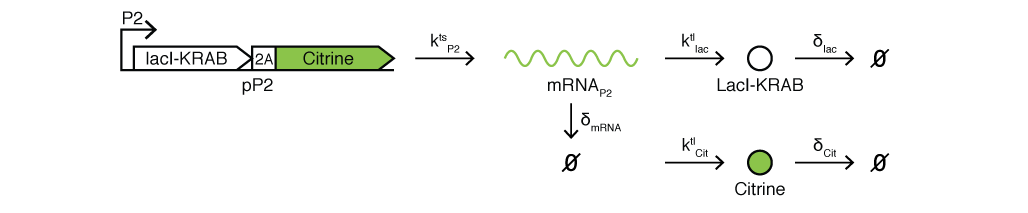  $\dot{Citrine}=\pi_{Cit}\cdot{mRNA}_{166}-\delta_{Cit}\cdot Citrine$  $\dot{mRNA_{P2}}=k_{P2}^{ts}\cdot P2 + k_{P2PIT}^{ts}\cdot P2_{PIT}+k_{P2Kni}^{ts}\cdot P2_{Kni}+k_{P2PITKni}^{ts}\cdot P2_{PITKni}-\delta_{mRNA}\cdot mRNA_{P2}$  $\dot{P2_{Kni}}=\left( k_{Kni}^{on}\cdot P2\cdot Kni^{2.1}-k^{off}\cdot P2_{Kni} \right)-\left( k_{P2_{PIT}}^{on}\cdot P2_{Kni}\cdot PIT-k^{off}\cdot P2_{PITKni} \right)-\left( \delta_{Kni}+\delta_{DNA} \right)\cdot P2_{Kni}$  $\dot{P2_{PITKni}}=\left( k_{Kni}^{on}\cdot P2_{PIT}\cdot Kni^{2.1}-k^{off}\cdot P2_{PITKni} \right)+\left( k_{P2_{PIT}}^{on}\cdot P2_{Kni}\cdot PIT-k^{off}\cdot P2_{PITKni} \right)-\left( \delta_{PIT}+\delta_{DNA} \right)\cdot P2_{PITKni}$  $\dot{P2_{PIT}}=\left( k_{P2PIT}^{on}\cdot P2\cdot PIT-k^{off}\cdot P2_{PIT} \right)-\left( k_{Kni}^{on}\cdot P2_{PITKni}\cdot Kni^{2.1}-k^{off}\cdot P2_{PITKni} \right)-\left( \delta_{PIT}+\delta_{DNA} \right)\cdot P2_{PIT}$  $\dot{P2}=-\left( k_{P2PIT}^{on}\cdot P2\cdot PIT-k^{off}\cdot P2_{PIT} \right)-\left( k_{Kni}^{on}\cdot P2\cdot Kni^{2.1}-k^{off}\cdot P2_{Kni} \right)+\delta_{PIT}\cdot\left( P2_{PIT}+P2_{PITKni} \right)+ \delta_{Kni}\cdot P2_{Kni}-\delta_{DNA}\cdot P2$ |

### RIFFM circuit

$$\dot{Kni}=\pi_{Kni}\cdot{mRNA}_{P1}-2.1\cdot(k_{Kni}^{on}\cdot Kni^{2.1}\cdot\left( P2+P2_{PIT} \right)-k^{off}\cdot(P2_{Kni}+P2_{PITKni})-\delta_{Kni}\cdot Kni$$

$$\dot{lacI}=\pi_{lac}\cdot{mRNA}_{P2}-4\cdot\left( k_{lac}^{on}\cdot lac^{4}\cdot(P1+P1_{PIT})-k^{off}\cdot(P1_{lac}+P1_{PITlac}) \right)-\delta_{lac}\cdot lac$$

### I1-FFL1 circuit

$$\dot{Kni}=0\cdot{mRNA}_{P1}-2.1\cdot(k_{Kni}^{on}\cdot Kni^{2.1}\cdot\left( P2+P2_{PIT} \right)-k^{off}\cdot(P2_{Kni}+P2_{PITKni})-\delta_{Kni}\cdot Kni$$

$$\dot{lacI}=\pi_{lac}\cdot{mRNA}_{P2}-4\cdot\left( k_{lac}^{on}\cdot lac^{4}\cdot(P1+P1_{PIT})-k^{off}\cdot(P1_{lac}+P1_{PITlac}) \right)-\delta_{lac}\cdot lac$$

### I1-FFL2 circuit

$$\dot{Kni}=\pi_{Kni}\cdot{mRNA}_{P1}-2\cdot(k_{Kni}^{on}\cdot Kni^{2.1}\cdot\left( P2+P2_{PIT} \right)-k^{off}\cdot(P2_{Kni}+P2_{PITKni})-\delta_{Kni}\cdot Kni$$

$$\dot{lacI}=0\cdot{mRNA}_{P2}-4\cdot\left( k_{lac}^{on}\cdot lac^{4}\cdot(P1+P1_{PIT})-k^{off}\cdot(P1_{lac}+P1_{PITlac}) \right)-\delta_{lac}\cdot lac$$

### FO circuit

$$\dot{Kni}=0\cdot{mRNA}_{P1}-2\cdot(k_{Kni}^{on}\cdot Kni^{2.1}\cdot\left( P2+P2_{PIT} \right)-k^{off}\cdot(P2_{Kni}+P2_{PITKni})-\delta_{Kni}\cdot Kni$$

$$\dot{lacI}=0\cdot{mRNA}_{P2}-4\cdot\left( k_{lac}^{on}\cdot lac^{4}\cdot(P1+P1_{PIT})-k^{off}\cdot(P1_{lac}+P1_{PITlac}) \right)-\delta_{lac}\cdot lac$$

### Coherent feedforward loop circuit


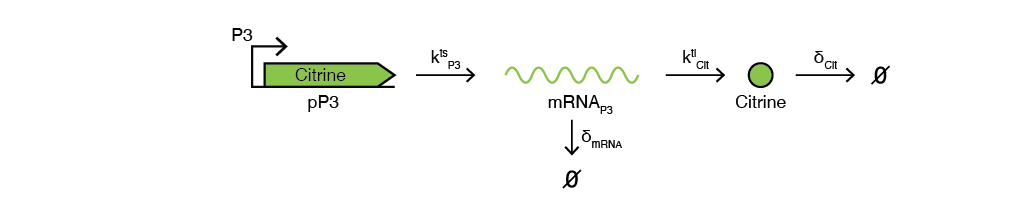

$$\dot{pP3}=-\delta_{DNA}\cdot pP3-k_{PIT}^{on}\cdot pP3\cdot PIT+k^{off}\cdot pP3\_PIT+\delta_{PIT}\cdot pP3\_PIT$$

$$\dot{pP3\_PIT}= k_{PIT}^{on}\cdot pP3\cdot PIT-k^{off}\cdot pP3\_PIT-\delta_{DNA}\cdot pP3-\delta_{PIT}\cdot pP3\_PIT$$

$$\dot{mRNA_{P3}}=k_{P3}^{ts}\cdot pP3+k_{P3\_PIT}^{ts}\cdot P3_{PIT}-\delta_{mRNA}\cdot mRNA_{P3}$$

$$\dot{Citrine}=\pi_{Cit}\cdot{mRNA}_{P3}-\delta_{Cit}\cdot Citrine$$

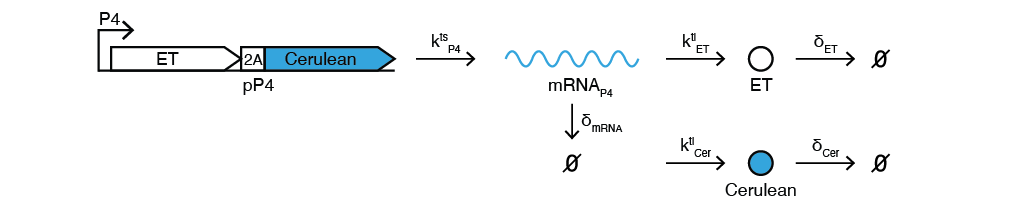

$$\dot{pP4}=-\delta_{DNA}\cdot pP4-k_{PIT}^{on}\cdot pP4\cdot PIT+k^{off}\cdot pP4\_PIT-k_{ET}^{on}\cdot pP4\cdot ET+k^{off}\cdot pP4\_ET+\delta_{PIT}\cdot\left( pP4\_PIT+pP4\_ET\_PIT \right)+\delta_{ET}\cdot pP4\_ET$$

$$\dot{pP4\_ET}=k_{ET}^{on}\cdot pP4\cdot ET-k^{off}\cdot pP4\_ET-k_{PIT}^{on}\cdot pP4\_ET\cdot PIT+k^{off}\cdot pP4\_ET\_PIT-\delta_{DNA}\cdot pP4-\delta_{ET}\cdot pP4\_ET$$

$$\dot{pP4\_ET\_PIT}=k_{ET}^{on}\cdot pP4\_ET\cdot PIT-k^{off}\cdot pP4\_ET\_PIT+k_{PIT}^{on}\cdot pP4\_ET\cdot PIT-k^{off}\cdot pP4\_ET\_PIT-\delta_{DNA}\cdot pP4\_ET\_PIT-\delta_{PIT}\cdot pP4\_ET\_PIT$$

$$mRNA_{P4}=k_{P4\_PIT}^{ts}\cdot pP4\_PIT+k_{P4\_ET}^{ts}\cdot pP4\_ET+k_{P4\_ET\_PIT}^{ts}\cdot pP4\_ET\_PIT+k_{P4}^{ts}\cdot pP4-\delta_{mRNA}\cdot mRNA_{P4}$$

$$\dot{ET}=\pi_{ET}\cdot mRNA_{P4}-k_{ET}^{on}\cdot pP4\cdot ET+k^{off}\cdot pP4\_ET-k_{ET}^{on}\cdot pP4\_ET\cdot PIT+k^{off}\cdot pP4\_ET\_PIT-\delta_{ET}\cdot ET$$

### Negative feedback circuit


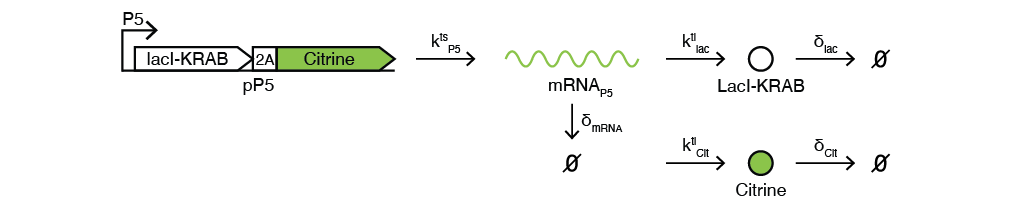

$$\dot{pP5}=-\delta_{DNA}\cdot pP5-k_{PIT}^{on}\cdot pP5\cdot PIT+k^{off}\cdot pP5\_PIT-k_{lacI}^{on}\cdot pP5\cdot lacI^{4}+k^{off}\cdot pP5\_lacI+\delta_{PIT}\cdot pP5\_PIT+\delta_{lacI}\cdot pP5\_lacI+\delta\_PIT\cdot pP5\_PIT\_lacI$$

$$\dot{pP5\_PIT}=k_{PIT}^{on}\cdot pP5\cdot PIT-k^{off}\cdot pP5\_PIT-k_{lacI}^{on}\cdot pP5\_PIT\cdot lacI^{4}+k^{off}\cdot pP5\_PIT\_lacI-\delta_{DNA}\cdot pP5\_PIT-\delta_{PIT}\cdot pP5\_PIT$$

$$\dot{pP5\_lacI}=k_{lacI}^{on}\cdot pP5\cdot lacI^{4}-k^{off}\cdot pP5\_lacI-k_{PIT}^{on}\cdot pP5\_lacI\cdot PIT+k^{off}\cdot pP5\_PIT\_lacI-\delta_{DNA}\cdot pP5\_lacI-\delta_{lacI}\cdot pP5\_lacI$$

$$\dot{pP5\_PIT\_lacI}=k_{lacI}^{on}\cdot pP5\_PIT\cdot lacI^{4}-k^{off}\cdot pP5\_PIT\_lacI+k_{PIT}^{on}\cdot pP5\_lacI\cdot PIT-k^{off}\cdot pP5\_PIT\_lacI-\delta_{DNA}\cdot pP5\_PIT\_lacI-\delta_{PIT}\cdot pP5\_PIT\_lacI$$

$$\dot{mRNA_{P5}}=-\delta_{mRNA}\cdot mRNA_{P5}+k_{P5\_PIT}^{ts}\cdot pP5\_PIT+k_{P5\_lac}^{ts}\cdot pP5\_lacI+k_{P5\_PIT\_lac}^{ts}\cdot pP5\_PIT\_lacI+k_{P5}^{ts}\cdot pP5$$

$$\dot{lacI}=\pi_{lac}\cdot{mRNA}_{P5}-4\cdot\left( k_{lac}^{on}\cdot lac^{4}\cdot(P1+P1_{PIT})-k^{off}\cdot(P1_{lac}+P1_{PITlac}) \right)-\delta_{lac}\cdot lac$$

### Positive feedback circuit


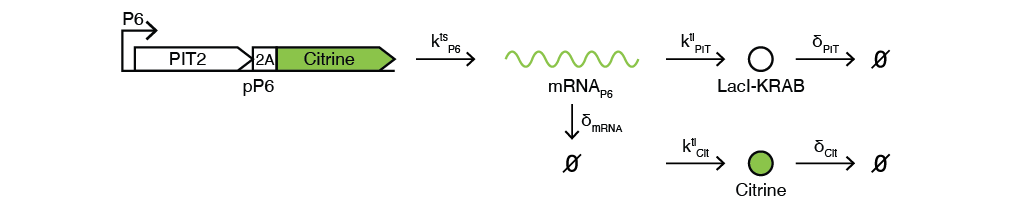

$$\dot{pP6}=-k_{PIT}^{on}\cdot pP6\cdot PIT^{2}+k^{off}\cdot pP6\_PIT+\delta_{PIT}\cdot pP6\_PIT-\delta_{DNA}\cdot pP6$$

$$\dot{pP6\_PIT}=k_{PIT}^{on}\cdot pP6\cdot PIT^{2}-k^{off}\cdot pP6\_PIT-\delta_{DNA}\cdot pP6\_PIT-\delta_{PIT}\cdot pP6\_PIT$$

$$\dot{mRNA_{P6}}=-\delta_{mRNA}\cdot mRNA_{P6}+k_{P6\_PIT}^{ts}\cdot pP6\_PIT+k_{P6}^{ts}\cdot pP6$$

$\dot{Citrine}=\pi_{Cit}\cdot{mRNA}_{P6}-\delta_{Cit}\cdot Citrine$
