# Supplementary material for: Precise determination of input-output mapping for multimodal gene circuits using data from transient transfection
Source: PLoS Comput Biol. 2020 Nov 30;16(11):e1008389. doi: 10.1371/journal.pcbi.1008389 (PMC7728399; doi:10.1371/journal.pcbi.1008389)
Supplement: S4 Text — (DOCX) [file pcbi.1008389.s004.docx]

S4 Text PFAFF User Manual

# General description and minimal data requirements

Peak finder analysis for flow cytometry data (PFAFF) is a MATLAB tool, which extracts input/output relationships for one or more gene circuits in a single program run, when provided with a particular set of circuit characterization data as described below. The total number of different circuits whose characterization data comprise the input dataset is indicated by a variable F in this manual. The characterization data arise from transient transfections experiments or from the simulation of such experiments, such as performed for example in the accompanying manuscript. The individual characterization data packages are collections of single-cell readouts that reflect, respectively, the expression of the circuit input, circuit output, and a multiplicity of transfection in this cell as reflected in the expression level of the protein encoded by the transfection reference gene. Such datasets can be obtained in flow cytometry experiments, where the input to a gene circuit is fused to a fluorescent reporter, and the output(s) are (fused to) another fluorescent reporter(s). However, similar datasets can be obtained during image analysis of fluorescent images, mass cytometry experiments, single-cell FISH experiments or antibody staining experiments. What is important is that every cell is described by a vector of values, of which one is designated as input, one or more are designated as outputs (the total number of outputs is denoted as P), and an additional value is designated as a reporter for the multiplicity of transfection. An experiment outcome has to be formatted as a table where every row corresponds to a single cell and every column corresponds to a particular readout.

To obtain extended input/output relationships, a number of such experimental datasets are required; they have to differ among themselves in the level of input expression per copy of a genetic template. A typical example could be an input driven by Doxycycline-inducible TRE promoter; another example could be a panel of constitutive promoters whose strength ranges from low to high.

Accordingly, the input to PFAFF has to encompass at least one, but ideally many, tabulated experimental or simulated datasets of the following format (compare Fig. S9 for indexes; C refers to the number of cells or events, M to the total number of readouts and Z to the total number of input modulations)

| Input modulation level 1 | | |
| --- | --- | --- |
| [Cell_1,1__Readout_1_] | ... | [Cell_1,1__Readout_M_] |
| ... | ... | ... |
| [Cell_1,C__Readout_1_] | ... | [Cell_1,C__Readout_M_] |

...

| Input modulation level Z | | |
| --- | --- | --- |
| [Cell_Z,1__Readout_1_] | ... | [Cell_Z,1__Readout_M_] |
| ... | ... | ... |
| [Cell_Z,C__Readout_1_] | ... | [Cell_Z,C__Readout_M_] |

# Prepare data for input

The files containing the tables above (one table per file) must be in comma separated values (.csv) format that can be imported into MATLAB. In general, there are multiple ways to achieve this. As an example, we outline how to generate such tables using FlowJo: After importing .fcs-files into FlowJo and subsequent gating of the desired events, the data set is exported using the built-in function in FlowJo by right click on the file and exporting the **Scaled Values** as a .csv-file. This can also be done for a group of files.

Consider preparing lookup tables for easy interpretation of the output data

***Circuit look up table***

| Full name/description of the circuit | Shorthand notation to use in the input file name, aka [Circuit Name] below |
| --- | --- |
| A fan out circuit with PIT2 inducing Cerulean and Citrine | FO |
| An incoherent feed-forward motif circuit, with the input inducing LacI repressor and output, LacI repressing the output | I1FFL1 |
| ... | ... |

***Input modulation lookup table***

| Input modulator level in an experiment | Shorthand notation to be used in the input file name and in the output [Input_Modulation_Level] |
| --- | --- |
| Dox = 0 | Input01 |
| Dox = 1 ng/mL | Input02 |
| ... | ... |
| Dox = 10 ug/mL | Input0Z [Z: total number of different input modulation levels used in the experiment] |

***Replicate lookup table***

| Full description of a replicate experiment | Shorthand notation to be used in the input file name and in the output [Rep0R] |
| --- | --- |
| Done on 01.01.2021, data folder: /NNN/ | Rep01 |
| Done on 03.01.2021, data folder: /NNN/ | Rep02 |
| ... | ... |
| Done on 09.01.2021, data folder: /NNN/ | Rep0R [R: total number of replicates] |

***Readouts lookup table***

| Full description of a readout | Column number in the raw data file | ID number in the output. This is always a running number beginning at 1, the numbering corresponds to the order of passing the parameters to the PFAFF function |
| --- | --- | --- |
| Transfection multiplicity reporter SBFP | 5 | N/A |
| Input mCherry | 3 | Input |
| Output mCerulean | 2 | Output1 |
| Output mCitrine | 6 | Output2 |
| … | … | … |

**Note 1:** When multiple circuits are analyzed in a single PFAFF run, the number of input modulation levels (Z) for each circuit **must** be the same between experiments, i.e. Z must be the same for each circuit. If the data set is collected in R replicates, this number of replicates **must** be the same for all circuits and all input modulation levels, i.e. every input modulation level for each circuit must be measured or simulated R times.

The .csv-files must be named in a standardized way. The provided import function (‘Import_for_PFAFF’) can be used if the file names follow the format:

[DataID]_[Circuit Name]_[Input Modulation Level]_Rep[R].csv

Here, ‘[DataID]’ refers to an alphanumeric string that is numbered sequentially at its terminus (e.g. [DataID] = Tube001, Tube002, Tube003, etc.). The string must be the same for each .csv-file, only differing in the attached number, thus creating a **unique** [DataID] for each file in the data set. Often, this is done automatically by the acquisition software (e.g. BD FACSDiva, NIS-Elements). ‘[Circuit Name]’ indicates the name of the circuit to be analyzed and may contain alphanumeric characters (e.g. [Circuit Name] = RIFFM, I1FFL1, FO etc.). This tag will also be displayed as the circuit labels throughout the analysis with PFAFF. Use *Circuit Lookup Table* to record the names. ‘[Input Modulation Level]’ refers to the input modulation of the sample. It may contain alphabetic characters only, followed by a number. The numbers must range from 1 to Z and correspond to the input modulation level as defined in an *Input Modulation Lookup Table*, and this must be a running number starting at 1, i.e. the sample with input modulation level z must contain the number z at the end of the [Input Modulation Level] string (e.g. [Input Modulation Level] = Input01, Input02, Input03, etc.). ‘[R]’ indicates replicate series the sample is part of. [R] must only be an integer, e.g. [R] = 1, 2, 3, etc. All other characters of the .csv-file name, i.e. parts that are not enclosed by square brackets, must match the format above. Even if the experiment only exists in a single replicate, add Rep1 to the file name.

*Examples of file names readable by the provided import function:*

*Import 1 circuit with 4 input modulations and 1 replicate:*

Tube001_RIFFM_Input01_Rep1.csv
Tube002_RIFFM_Input02_Rep1.csv
Tube003_RIFFM_Input03_Rep1.csv
Tube004_RIFFM_Input04_Rep1.csv

*Import 2 circuits with 2 input modulations and 1 replicate:*

Tube001_RIFFM_Input01_Rep1.csv
Tube002_RIFFM_Input02_Rep1.csv
Tube013_I1FFL1_Input01_Rep1.csv
Tube014_I1FFL1_Input02_Rep1.csv

*Import 1 circuit with 2 input modulations and 2 replicates:*

Tube089_FO_Input01_Rep2.csv
Tube090_FO_Input02_Rep2.csv
Tube137_FO_Input01_Rep3.csv
Tube138_FO_Input02_Rep3.csv

Each file contains a table consisting of C rows and M columns that correspond to the number of recorded events (i.e. cells) and the number of measured readouts, respectively. Thus, the size of the table is $C\times M$.

**Note 2:** The number of recorded events/cells does not have to be the same for all files. In fact, it can vary for every circuit, input modulation and replicate. However, **within** a single file the number of measured events/cells must be equal among all readouts, i.e. each readout in column#1 must have the same length as the readout in column#2, etc.

The readouts of the transfection reference, the input, and outputs (number of outputs P is not limited) are passed to PFAFF. Therefore, column numbers representing the transfection reference, the input, and the outputs from each .csv-file, must be known to the user. For the remainder of this manual, we will use the following notation (refer to ***Readouts Lookup Table***):

A column number between 1 and M in which the transfection reference readout is stored:

ColumnTM

A column number between 1 and M in which the input readout is stored:

ColumnInput

A column number between 1 and M in which output #1 readout is stored:

ColumnOutput_1

[a number of P output columns can be defined]

...

A column number between 1 and M in which the output #P readout is stored:

ColumnOutput_P

**Note 3:** For all files, same columns must correspond to the same readouts; the total number of readouts M must be the same in all .csv-files.

# Import data into MATLAB workspace

## Automated import using a provided function

Add the folder ‘PFAFF/’ and its subfolders to your MATLAB path:

>>addpath(genpath(‘PFAFF/’));

Move all .csv-files that should be analyzed to a separate folder (e.g. ‘MyData/’), change your current working directory (‘cd MyData’) to that folder and run the provided import function:

>>[MyDataStruct,MyLabels] = Import_for_PFAFF;

The automated import function generates two outputs; (1) the data assigned to the structure ‘MyDataStruct’ and the structure ‘MyLabels’ containing labels extracted from the .csv-files, i.e. the circuit names, input names and replicate names. These structures can be passed to PFAFF to run the analysis (see section 6).

## Manual import

Alternatively, the input variable ‘MyDataStruct’ can also be constructed manually. Start by assigning the data, i.e. the content of each .csv-file, into a cell array, e.g. called ‘FlowData’, of the size $F\times Z$. The cells within that cell array contain the single-cell datasets from individual characterization experiments (i.e. corresponding to individual tables from each .csv-file as outlined in section 1) from a given circuit f (f = 1, ..., F) at a specific input modulation level z (z = 1, ..., Z). Thus, rows in the cell array ‘FlowData’ correspond to input modulation levels of a single circuit f, while columns correspond to the different circuits characterized for a given single input level z.

*Example for assigning the data in a file ‘csvFile_Circuit_f_Input_z.csv’ to the cell array ‘FlowData’ at row position f and column position z:*

FlowData{f,z} = csvread(‘csvFile_Circuit_f_Input_z.csv’,1,0);

*Example for the content of cell ‘FlowData{1,1}’ (data from circuit f = 1 and input modulation z = 1):*

| [Cell_1,1__Readout_1_(Circuit_1_)] | ... | [Cell_1,1__Readout_M_(Circuit_1_)] |
| --- | --- | --- |
| ... | ... | ... |
| [Cell_1,C__Readout_1_(Circuit_1_)] | ... | [Cell_1,C__Readout_M_(Circuit_1_)] |

*Example for the content of cell ‘FlowData{4,3}’ (data from circuit f = 4 and input modulation z = 3):*

| [Cell_3,1__Readout_1_(Circuit_4_)] | ... | [Cell_3,1__Readout_M_(Circuit_4_)] |
| --- | --- | --- |
| ... | ... | ... |
| [Cell_3,C__Readout_1_(Circuit_4_)] | ... | [Cell_3,C__Readout_M_(Circuit_4_)] |

This cell array is grouped into a structure named according to its replicate index r (r = 1, ..., R), and has to be named ‘Rep_r’:

Rep_r.FlowData{f,z} = FlowData{f,z};

*Example for replicate structure r = 1:*

Rep_1.FlowData{f,z} = FlowData{f,z};

*Example for replicate structure r = 3:*

Rep_3.FlowData{f,z} = FlowData{f,z};

Lastly, all replicate variables are grouped into the structure variable ‘MyDataStruct’:

MyDataStruct = struct(‘Rep_1’,Rep_1,’Rep_2’,Rep_2,...,’Rep_R’,Rep_R);

*Example for concatenating R = 3 replicates into the MyDataStruct:*

MyDataStruct = struct(‘Rep_1’,Rep_1,’Rep_2’,Rep_2,’Rep_3’,Rep_3);

The requirement from **Note 1** also apply for the manual import of files: Z must be the same for each circuit. If the data set is collected in R replicates, the number of replicates **must** be the same for all circuits and all input modulation levels, i.e. every input modulation level for each circuit must be measured or simulated R times.

Similar to creating the ‘MyDataStruc’ manually, the user can also create the ‘MyLabels’ manually. The structure ‘MyLabels’ contains 3 cell arrays: (1) ‘Circuit’, stores all labels for each circuit as strings, i.e. FO, I1FFL1, RIFFM, etc., (2) ‘Input’ stores all labels for each input modulation levels as strings, i.e. Input01, Input02, Input03, etc. and (3) ‘Replicate’ stores all labels for each replicates as strings, i.e. Rep1, Rep2, Rep3, etc.

# (Optional) Save the variable as a MATLAB workspace

After importing the files into a PFAFF-readable format/variable (‘MyDataStruct’), the structure can be saved using the built-in MATLAB function:

>>save MyData_PFAFF_format.mat MyDataStruct MyLabels

# (Optional) Setting parameters for bi-exponential transformation

For fitting and illustration purposes PFAFF employs a bi-exponential transformation of the data set. The default settings ($\mathcal{M}$ = 4.5; $\mathcal{p}$ = 2; $\mathcal{T}$ = 262144; $\mathcal{W}$ = 0.401) cover a range of [-62, 262144] units. If the data set requires a different range or adjustment of the transformation parameter, the user has to re-define them manually in the structure ‘biex’ within the ‘PFAFF.m’ file. For a detailed description of each parameter ‘$\mathcal{M}$’, ‘$\mathcal{p}$’, ‘$\mathcal{T}$’ and ‘$\mathcal{W}$’, the user is kindly referred to Parks et al.

# Execute the function PFAFF

If not done in any of the previous steps, add the folder ‘PFAFF/’ and its subfolders to your MATLAB path:

>>addpath(genpath(‘PFAFF/’));

In order to analyze the data set using PFAFF, the structure variable ‘MyDataStruct’ (and optional ‘MyLabels’) has to be in the active workspace. If the import was done earlier, one can simply use the variable and pass it to PFAFF. Otherwise, the saved workspace ‘MyData_PFAFF_format.mat’ must be loaded:

>>load ‘MyData_PFAFF_format.mat’;

Afterwards, PFAFF can be executed by calling:

>>PFAFF([MyDataStruct],[B],[ColumnTM],[ColumnInput],...

[ColumnOutput_1],[ColumnOutput_2],...,[ColumnOutput_P],...

‘Labels’,’MyLabels’);

The set of input arguments for PFAFF consists of the structure variable [MyDataStruct], which contains all data subjected for analysis (see section 3), one integer value ‘[B]’, that corresponds to the number of bins the data set should be divided into (bins are ordered from highest intensity of the transfection marker signal, i.e. bin 1 to lowest intensity of the transfection marker signal, i.e. bin B), two integer values that correspond to the column number of the transfection reference (‘[ColumnTM]’) and the column number of the input (‘[ColumnInput]’), P integer values, each corresponding to the column number of each respective output ('[ColumnOutput_1], [ColumnOutput_2], …, [ColumnOutput_P]’) and an optional Name-Value pair (‘Labels’,’MyLabels’) that allow passing of labels (i.e. the structure ‘MyLabels’) from the .csv-filenames to PFAFF.

*Example for a dataset (‘MyDataStruct’) with P = 1 output that should be split into 10 bins, with the readouts ColumnTM = 1, ColumnInput = 2, ColumnOutput_1 = 3.*

>>PFAFF(MyDataStruct,10,1,2,3);

*Example for a dataset (‘MyDataStruct’) with P = 4 outputs that should be split into 5 bins, with the readouts ColumnTM = 6, ColumnInput = 15, ColumnOutput_1 = 9, ColumnOutput_2 = 12, ColumnOutput_3 = 18, ColumnOutput_4 = 21.*

>>PFAFF(MyDataStruct,5,6,15,9,12,18,21);

*Example for a dataset (‘MyDataStruct’) with P = 2 outputs that should be split into 20 bins, with the readouts ColumnTM = 1, ColumnInput = 2, ColumnOutput_1 = 3, ColumnOutput_2 = 4 and the label structure ‘MyLabels’ as an Name-Value pair argument.*

>>PFAFF(MyDataStruct,20,1,2,3,4,’Label’,’MyLabels’);

**Note 4:** The column indices from the readout files are only used as input arguments for the PFAFF function. The sequence of the ColumnOutput arguments passed to PFAFF will determine the output index p in the results (see section 7, also ***Readouts Lookup Table***).

# Results obtained by PFAFF

PFAFF generates a series of files, which are all saved in a folder generated in the current working directory. It is named ‘PFAFF_Results_[STRUC]’, whereas ‘[STRUC]’ refers to the name of the data input structure, e.g. if the data input structure is named ‘MyDataStruct’ the output folder will be ‘PFAFF_Results_MyDataStruct’. This folder contains (1) two MATLAB workspaces (‘DataSet.mat’ and ‘Analysis.mat’) and (2) all the figures generated during the analyses.

The sequence of input arguments to the PFAFF function will determine the index of the outputs. This means that the measurements from a readout p, which was stored in ColumnOutput_p will be labeled as Output_p for all **plots**.

*Example:* *The output measurements of a circuit consist of two fluorescent proteins, Cerulean and Citrine, that are stored in ColumnOutput_1 = 9 and ColumnOutput_2 = 12 in each .csv-file from section 1, respectively. The obtained results from the analysis with PFAFF will label the plots/folders with the index p = 1 for output_1 (Cerulean) and p = 2 for output_2 (Citrine);* ***not*** *output_9 and output_12.*

When dealing with In PFAFF output data structures, the **index** to retrieve the values of Output_p is q, q = p+2. Accordingly, the output index p is **not** equal to the index variable q.

*Example: Like in the example above with two output measurements of a circuit, i.e. two fluorescent proteins, Cerulean and Citrine, are stored in ColumnOutput_1 = 9 and ColumnOutput_2 = 12 in each .csv-file, respectively. The data structures resulting from PFAFF run will index those two outputs p = [1, 2] as q = 3 for output_1 (Cerulean) and q = 4 for output_2 (Citrine). For input/output mapping of input and output_1, the index q = 3 will be used in the output variables. Likewise, the input/output mapping of input/output_2 uses the index q = 4 for the output variable (see below 7.1.1).*

## MATLAB workspaces generated by PFAFF

PFAFF generates workspaces, which contain data and variables used and generated during the analysis run. These data structures contain all information needed to recreate all plots (section 7.2) and all values corresponding to the input/output mappings.

### DataSet.mat

The created ‘DataSet.mat’ (inside‘PFAFF_[STRUC]/’ folder) contains a workspace with the structure variable ‘DataSet’. It consists of a series of variables that are used to plot input/output mappings. The variables themselves are (stacked) cell arrays with indices f, b and q. They correspond to the circuit index f (f = 1, …, F), the selected bin b (b = 1, …, B) and the PFAFF output index for variables q (q = 3, …, P+2), respectively. The ‘DataSet’ structure contains at least three cell array variables: ‘X’ (input modes), ‘Y’ (output modes) and ‘W’ (relative weights among the output modes). For any given [Variable] of the structure ‘DataSet’, i.e. ‘X’, ‘Y’ or ‘W’, each cell contains a matrix of size $\left( 2\cdot Z\cdot R \right)\times2$. The matrix can be divided into two major parts:

| *[Variable]_mode_high* | *[Variable]_mode_low* |  |  |
| --- | --- | --- | --- |
| [Variable]_1,1,high_ | [Variable]_1,1,low_ | **Replicate 1** | *Input_mode_high* |
| … | … |  |  |
| [Variable]_Z,1,high_ | [Variable]_Z,1,low_ |  |  |
| … | … | **…** |  |
| [Variable]_1,R,high_ | [Variable]_1,R,low_ | **Replicate R** |  |
| … | … |  |  |
| [Variable]_Z,R,high_ | [Variable]_Z,R,low_ |  |  |
| [Variable]_1,1,high_ | [Variable]_1,1,low_ | **Replicate 1** | *Input_mode_low* |
| … | … |  |  |
| [Variable]_Z,1,high_ | [Variable]_Z,1,low_ |  |  |
| … | … | **…** |  |
| [Variable]_1,R,high_ | [Variable]_1,R,low_ | **Replicate R** |  |
| … | … |  |  |
| [Variable]_Z,R,high_ | [Variable]_Z,R,low_ |  |  |

While the top half corresponds to values extracted from datasets around the high input mode, the bottom half corresponds to values extracted from the low input mode data set. Each row corresponds to an input modulation level z (z = 1, …, Z). Replicate values of [Variable] are attached below each set of input modulations so that rows [1, ..., Z] correspond to replicate 1, rows [Z+1, ..., 2Z] correspond to replicate 2, etc. The matrix has two columns. The left column corresponds to the high mode of the [Variable]’s distribution and the right column to low mode of the [Variable]’s distribution. In case of monomodal distributions, the ‘low’ mode values are substituted by a ‘NaN’ (‘Not a Number’) string.

##### Output’s mode weights W

W{q}{f,b}

PFAFF fits Gaussians to distributions of (input and) outputs. For bi-modal distributions the fits are convoluted Gaussians. The cell array ‘W’ is a relative measure for the area under each of the individually fitted Gaussian for bi-modal distributions of output p (p = q – 2) in circuit f (f = 1, …, F) and bin b (b = 1, …, B). The sum of each row z (z = 1, …, Z) is 1.

##### Input’s mode positions ‘X’

X{f,b}

The cell array ‘X’ contains a numeric matrix that corresponds to the mode’s position of the fitted Gaussians to the input distributions of circuit f (f = 1, …, F) at an input modulation level z (z = 1, …, Z) in bin b (b = 1, …, B), i.e. $Y_{z,f}^{MODE}\left( Y_{ColumnTM}(b) \right)$. Note that the columns (left and right) of the Matrix are identical.

##### Output’s mode positions ‘Y’

Y{q}{f,b}

The cell array ‘Y’ contains a numeric matrix that corresponds to the modes’ positions of Gaussians fitted to the distributions of output p (p = q - 2) of circuit f (f = 1, …, F) at an input modulation level z (z = 1, …, Z) in bin b (b = 1, …, B), i.e. $Y_{p,f}^{MODE}\left( Y_{z,f}^{MODE}\left( Y_{ColumnTM}(b) \right) \right)$.

##### Mean variables ‘W_bar’, ‘X_bar’ and ‘Y_bar’

In case of replicates, the mean of all replicates for each (applicable) [Variable] is available and denoted with a ‘_bar’ extension. In addition to the mean variables (‘_bar’ extension), the structure ‘DataSet’ further contains the lower and upper bounds of the standard deviation of [Variable] across all replicates. For a [Variable], i.e. ‘W_bar’, ‘X_bar’, ‘X_SD_lb’, ‘X_SD_ub’, ‘Y_bar’, ‘Y_SD_lb’ and ‘Y_SD_ub’, each cell contains a matrix is of size $\left( 2\cdot Z \right)\times2$ and the structure is illustrated below:

| *[Variable]_mode_high* | *[Variable]_mode_low* |  |  |
| --- | --- | --- | --- |
| [Variable]_1,1,high_ | [Variable]_1,1,low_ | **Mean of Replicates** | *Input_mode_high* |
| … | … |  |  |
| [Variable]_Z,1,high_ | [Variable]_Z,1,low_ |  |  |
| [Variable]_1,1,high_ | [Variable]_1,1,low_ | **Mean of Replicates** | *Input_mode_low* |
| … | … |  |  |
| [Variable]_Z,1,high_ | [Variable]_Z,1,low_ |  |  |

*Examples for plotting input/output mappings:*

*Scatter plot of input/output_1 mapping for circuit 3, bin 5*

>>scatter(DataSet.X{3,5}(:),DataSet.Y{3}{3,5}(:));

*Scatter plot of weighted input/output_2 mapping for circuit 2, bin 3*

>>scatter(DataSet.X{2,3}(:),DataSet.Y{4}{2,3}(:),50*DataSet.W{4}{2,3}(:));

*Scatter plot of weighted input/output_3 mapping for circuit 1, bin 1, averaged over all replicates*

>>scatter(DataSet.X_bar{1,1}(:),DataSet.Y_bar{5}{1,1}(:),...
 50*DataSet.W_bar{5}{1,1}(:));

*Scatter plot of input/output_1 mapping for circuit 1, bin 5, averaged over all replicates including the standard deviation as error bars*

>>errorbar(DataSet.X_bar{1,5}(:),DataSet.Y_bar{3}{1,5}(:),...
 DataSet.Y_SD_lb{3}{1,5}(:),DataSet.Y_SD_ub{3}{1,5}(:))

### Analysis.mat

All data that is used for PFAFF and generated while running it is stored in the workspace ‘Analysis.mat’ (be aware of its size). The structure ‘Data’ contains all raw data as well as every intermediate step, which can be useful for in-depth analysis and debugging (see section 7.1.2.2). Furthermore, the workspace contains the structure variable ‘DataSet’ (description of its content in section 7.1.1) and a structure named ‘ID’. The latter contains all label information gathered and used throughout the analysis (see section 7.1.2.1).

#### ID structure

**Replicates** refers to the number of replicates in the entire data set.

**ReplicateField** contains the name of the replicates in the data structure.

**DataVariable** stores the name of the input data structure.

**DataSize** stores the number of circuits F and input modulation levels Z.

**Circuits** refers to the number of circuits F.

**InputLvl** refers to the number of input modulation levels Z.

**TMBins** refers to the number of bins B the data set should be divided into.

**colTM** refers to the column of the transfection reference in the raw data set.

**colIn** refers to the column of the input readout in the raw data set.

**colOut** refers to the column(s) of the output(s) readout in the raw data set.

**Label** of the transfection reference, input and output(s) for annotating plots and figures.

**NrOut** refers to the total number of outputs P.

**StructName** refers to the name of the data structure from the input argument.

**FlowScale** contains the transformed values [0, 100, 1000, 10000, 100000] in bi-exponential space.

**Circuit** contains the labels of each circuit f as a string.

**Peak** contains the labels of high and low modes.

#### Data structure

Note that unless specified otherwise, the columns of the numeric matrices always refer to the sequence of input arguments to PFAFF, i.e. column 1: transfection reference; column 2: input; column 3: output_1; column 4: output_2; etc. It may however be, that a column remains empty. This is often the case for the transfection reference column.

**Data{f,z}** is a cell array that contains the raw data set of circuits f (f = 1, …, F) in rows and input modulation levels z (z = 1, …, Z) in columns. The matrices in each cell contain the same information as the .csv-files. Their size is $C\times M$.

**DataArray{f,z}** is a cell array of circuits f (f = 1, …, F) in rows and input modulation levels z (z = 1, …, Z) in columns. The matrix in each cell contains the readouts (in that order) of: (1) transfection reference, (2) input, (3) output_1, (4) output_2, etc.

**binned_events{f,z}{b}** is a stacked cell array of circuits f (f = 1, …, F) in rows and input modulation levels z (z = 1, …, Z) in columns. Each cell contains itself a cell array that correspond to the subset of data within a bin b (b = 1, …, B). The matrix in each cell contains the readouts (in that order) of: (1) transfection reference, (2) input, (3) output_1, (4) output_2, etc.

**biex_data{f,z,b}** is a 3-dimensional cell array containing circuits f (f = 1, …, F) in index 1, input modulations z (z = 1, …, Z) in index 2 and bins b (b = 1, …, B) in index 3. Each cell contains the bi-exponential transformed data of the readouts (in that order): (1) transfection reference, (2) input, (3) output_1, (4) output_2, etc.

**comp_biex{f,b}** is a cell array with circuits f (f = 1, …, F) in rows and bins b (b = 1, …, B) in columns. Each cell contains the concatenated data set of a circuit f at all input levels [1, …, Z] for a bin b. This data set is used for plotting the composite plots (see section 7.2.3.1). The matrix in each cell contains the concatenated readouts (in that order) of: (1) transfection reference, (2) input, (3) output_1, (4) output_2, etc.

**N{f,z,b,h}** refers to the counts of input or output distributions. It is a 4-dimensional cell array, with indices f (f = 1, …, F) for circuits, z (z = 1, …, Z) for input modulation levels, b (b = 1, …, B) for bins and h (h = 1,2) for the high or low peak of a bi-modal distribution. Each cell contains a matrix of the counts of the input distribution (column 2), output_1 distribution (column 3; p = 1), output_2 distribution (column 4; p = 2), etc.

**Mode_Input{f,z,b}** is a 3-dimensional cell array that refers to the parameters extracted from the Gaussian fits to the input distribution. The indices f, z and b refer to the circuit, input modulation level and bin, respectively. Each cell contains a 4x2 matrix, wherein column 1 refers to the fit of high mode and column 2 to the fit of low mode. The rows correspond to (1) height, (2) mode, (3) standard deviation and (4) area under the curve of the Gaussian fits.

**biex_data_Chypeak{f,z,b,h}** is a 4-dimensional cell array and contains all data points of the subset of data in a narrow window around the modes of the fitted Gaussians to the input distribution. The indices f, z, b and h refer to the circuit, input modulation level, bin and input distribution peak (h = 1, 2; high or low), respectively. The matrix in each cell contains the readouts (in that order) of: (1) transfection reference, (2) input, (3) output_1, (4) output_2, etc.

**Mode_Output{g}{h,f,z,b}** is a stacked, 4-dimensional cell array that refers to the parameters extracted from the Gaussian fits to the input distribution. The indices g, h, f, z and b refer to the output distribution peak (g = 1, 2; high or low), input distribution peak (h = 1, 2; high or low), circuit, input modulation level and bin, respectively. Each cell contains a 4x2 matrix, wherein column 1 refers to the fit of high mode and column 2 to the fit of low mode. The rows correspond to (1) height, (2) mode, (3) standard deviation and (4) area under the curve of the Gaussian fits.

## Figures generated by PFAFF

Besides the two MATLAB workspaces, PFAFF generates an array of figures, which are saved as .fig-files to allow extensive customization after running the analysis. All .fig-files are stored in different subfolders, grouped by their visual content.

The plots are labeled according to their circuit index f (alphabetically, ‘Circuit_A’, ‘Circuit_B’, ‘Circuit_C’, etc.), the bin number b (‘Bin1’, ‘Bin2’, ‘Bin3’, etc.), the output index p (‘Output_1’, ‘Output_2’, ‘Output_3’, etc.), the input mode h (‘Input_high_mode’ or ‘Input_low_mode’), the replicate index r (numeric ‘1’, ’2’, ‘3’, etc.) or a combination thereof.

### ‘IO_Plots/’

IO plot figures (.fig) show the input/output mapping (variables ‘X’ and ‘Y’, see section DataSet.mat) for each analyzed circuit, each output and each bin and each replicate in separate loglog-plots. All replicates are plotted on the same chart. The size of the dots represents the relative weight ‘W’ in bi-modal cases (see variable ‘W’ below). Separate figures are generated for combinations of outputs p (p = 1, …, P) and bins b (b = 1, …, B). The filenames for each figure specify the output p and the bin b that are shown in the plots and are generated according to this template:

Input_[Output_p]_Bin[b].fig

### ‘IO_Mean/’

In case the dataset contains more than one replicate (R > 1), this folder will be created. It contains similar figures as the ‘IO_Plots/’ folder. The plots show the averaged input/output mapping(s) (variables ‘X_bar’ and ‘Y_bar’) in loglog scale. Errorbars indicate the standard deviation between replicates. The filenames specify the output p and the bin b shown in the plots:

Input_[Output_p]_Bin[b]_Mean.fig

### ‘Repr/’

The folder ‘Repr/’ contains all plots specific to replicate r (r = 1, ..., R). A number of subfolders group the respective plots:

#### ‘Composite_Plots/’

Composite plots show concatenated data of one specific bin b for all input modulation levels. The composite data set is shown as false-color density plot on a bi-exponential scale. Superimposed as black circles is the input/output mapping extracted by PFAFF (variables ‘X’ and ‘Y’). The size of the black circle represents the relative weight ‘W’ in bi-modal cases. The figures are saved according to this template:

CompositeFlow_Plots_Rep[r]_Bin[b].fig

#### ‘Flow_Plots/’

False color density plots of raw experimental/simulated data on a bi-exponential scale. All circuits are plotted in one figure at different input modulation levels. Separate figures are generated for all combinations of input and outputs p and p' (p = 1, …, P; p’ ≠ p) and named according to this template:

FlowPlot_Input_v_Output_[p]_Rep[r].fig
FlowPlot_Output_[p]_v_Output_[p’]_Rep[r].fig

#### ‘InputPlots/’

Histograms of the input distributions for each circuit f (f = 1, …, F; rows) and input level z (z = 1, …, Z; columns) are shown for a particular bin b (b = 1, …, B). Modes, represented by red crosses, correspond to the fitted Gaussian distributions. The modes are labeled with either ‘b1’ or ‘b2’ that correspond to the $Y_{z}^{MODE,HIGH}(Y_{ColumnTM})$ and $Y_{z}^{MODE,LOW}(Y_{ColumnTM})$, respectively. The modes are saved in the variable ‘X’ and used for input/output mappings. Each plot has a unique title that refers to the circuit f, the bin b and input modulation level z, i.e. ‘[f][b], InputLvL [z]’.

*Examples for the plot title of circuit f = 1 (‘A’), bin b = 3, input modulation level z = 5*

A3, InputLvl 5

A separate figure is generated for each bin according to this template:

Bin[b]_Mode_Input_Rep[r].fig

#### ‘Output*p*_Plots/’

Similar to the folder ‘Input_Plots/’, the ‘Output*p*_Plots/’ folder stores all distributions for output p (p = 1, …, P). Since the output distributions depend on the mode (high or low) of the input distribution, the corresponding case is noted in the file name, whereas ‘Input_high_mode’ refers to the high mode and ‘Input_low_mode’ to the low mode of the input distribution ([Input Mode] = ‘Input_mode_high’ or ‘Input_mode_low’). Modes of fitted Gaussians to the output p’s distribution are indicated with red crosses and labels ‘b1’ or ‘b2’ correspond to the high and low modes, respectively. Each plot has a unique title that refers to the circuit f, the bin b and input modulation level z, i.e. ‘ [f][b], InputLvL [z]’.

*Examples for the plot title of circuit f = 3 (‘C’), bin b = 10, input modulation level z = 12*

C10, InputLvl 12

For every bin and input mode, a separate figure, depicting all circuits and input levels, is created and named according to the template:

Bin[b]_Mode_Output[p]_[Input Mode]_Rep[r].fig

As the number of outputs is not limited, the sum of Output_Plots folders is P.

# Acknowledgements of other MATLAB functions

cloudPlot
Daniel Armyr (2020). cloudPlot (https://www.mathworks.com/matlabcentral/fileexchange/23238-cloudplot), MATLAB Central File Exchange. Retrieved April 3, 2020.

# References

Parks, D. R., Roederer, M., and Moore, W. A. (2006) A new "Logicle" display method avoids deceptive effects of logarithmic scaling for low signals and compensated data, *Cytom Part A* *69a*, 541-551.
