# Supplementary material for: Precise determination of input-output mapping for multimodal gene circuits using data from transient transfection
Source: PLoS Comput Biol. 2020 Nov 30;16(11):e1008389. doi: 10.1371/journal.pcbi.1008389 (PMC7728399; doi:10.1371/journal.pcbi.1008389)
Supplement: S5 Text — (DOCX) [file pcbi.1008389.s005.docx]

S5 Text Description of the example data set

The example data set contains transient transfection simulations of F = 4 genetic circuits, labeled as ‘RIFFM’, ‘I1FFL1’, ‘I1FFL2’, ‘FO’. Each file corresponds to a single simulation of a given circuit at a specific input modulation z (z = 1, …, Z, Z = 12). The simulations were repeated R times, corresponding to individual replicate data sets (‘Rep1’,’Rep2’,’Rep3’; r = 1, …, R, R = 3). Thus, a given file name indicates all information about the initial condition of a single simulation, e.g.:

1. ‘Tube002_RIFFM_Input02_Rep1.csv’:
   corresponds to circuit ‘RIFFM’, input modulation 2 and replicate 1.
2. ‘Tube075_I1FFL2_Input03_Rep2.csv’:
   corresponds to circuit ‘I1FFL2’, input modulation 3 and replicate 2.
3. ‘Tube139_FO_Input07_Rep3.csv’:
   corresponds to circuit ‘FO’, input modulation 7 and replicate 3.

The .csv-files themselves contain 5 columns with numeric entries, that correspond to the measurements of the respective sample. A header is enclosed that represents the different measurement-channels (channel-IDs). The channel-IDs are: (1) ‘SBFP2’, (2) ‘Cerulean’, (3) ‘Citrine’, (4) ‘mCherry’, (5) ‘rtTA_DOX’ and they correspond to the ‘multiplicity marker’, ‘output_1’, ‘output_2’, ‘input’ and ‘internal_node’, respectively.

**Import data set into MATLAB and save a workspace**

Add the folder ‘PFAFF/’ and its subfolders to your path. Move all .csv-files that should be analyzed to a separate folder (e.g. ‘MyData/’), change your current working directory (‘cd MyData’) to that folder and run the provided import function:

>>[MyDataStruct,MyLabels] = Import_for_PFAFF;

Should the user choose to import only a subset, e.g. circuits ‘RIFFM’ and ‘FO’ at input modulations z = [1,5,6,7,8] and replicates r = [2,3], the folder must contain these files only. The total number of files in the folder ‘MyData/’ must be equal to the product of R F Z.

After importing the files into a PFAFF-readable format, the structures can be saved using the built-in MATLAB function:

>>save MyData_PFAFFformat.mat MyDataStruct MyLabels

This MATLAB workspace can then be used directly as an input for PFAFF.
