# Supplementary figures and images for: Precise determination of input-output mapping for multimodal gene circuits using data from transient transfection
Source: PLoS Comput Biol. 2020 Nov 30;16(11):e1008389. doi: 10.1371/journal.pcbi.1008389 (PMC7728399; doi:10.1371/journal.pcbi.1008389)

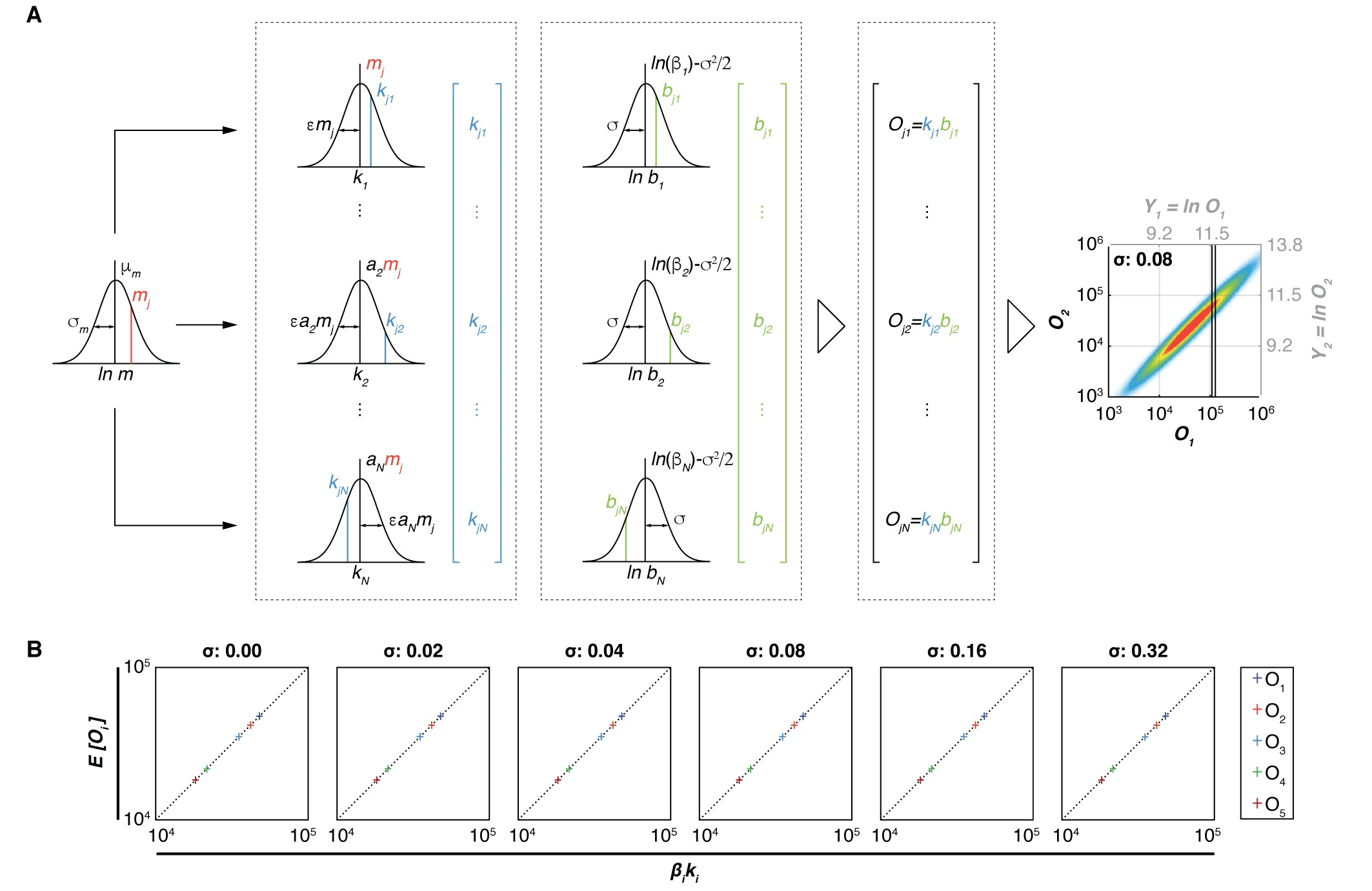

Supplement: S1 Fig — (TIF) [file pcbi.1008389.s006.tif]

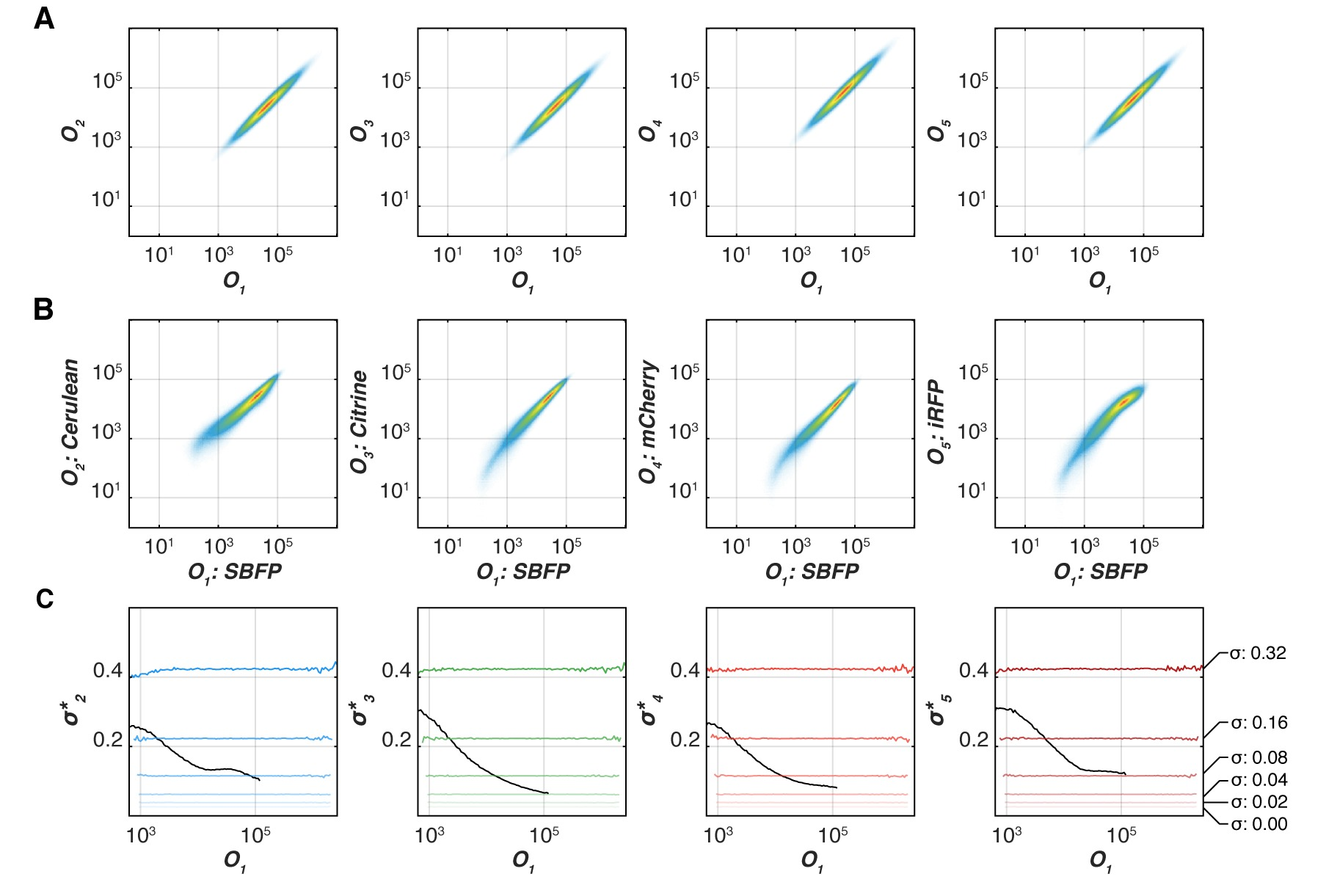

Supplement: S2 Fig — (TIF) [file pcbi.1008389.s007.tif]

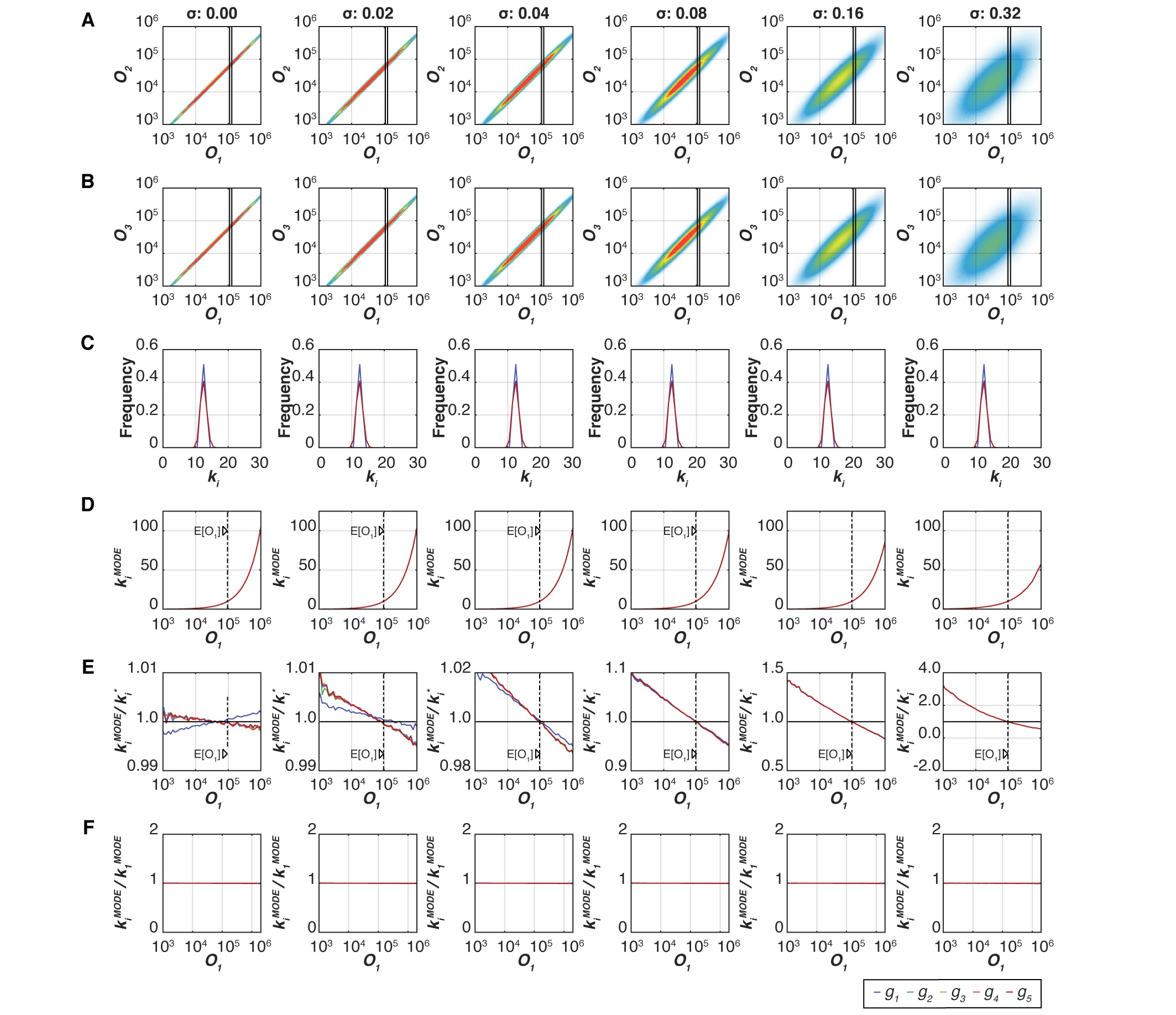

Supplement: S3 Fig — (TIF) [file pcbi.1008389.s008.tif]

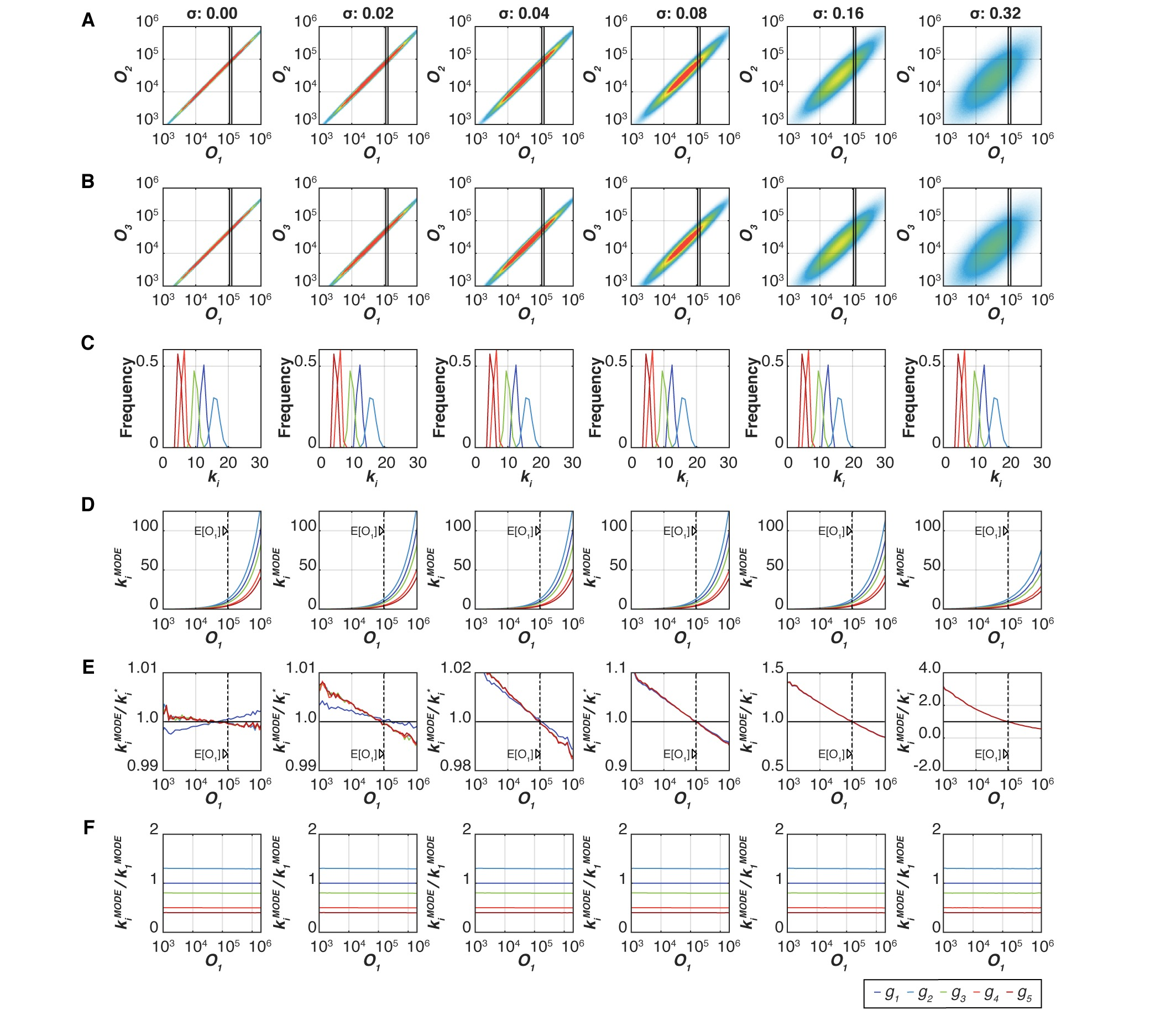

Supplement: S4 Fig — (TIF) [file pcbi.1008389.s009.tif]

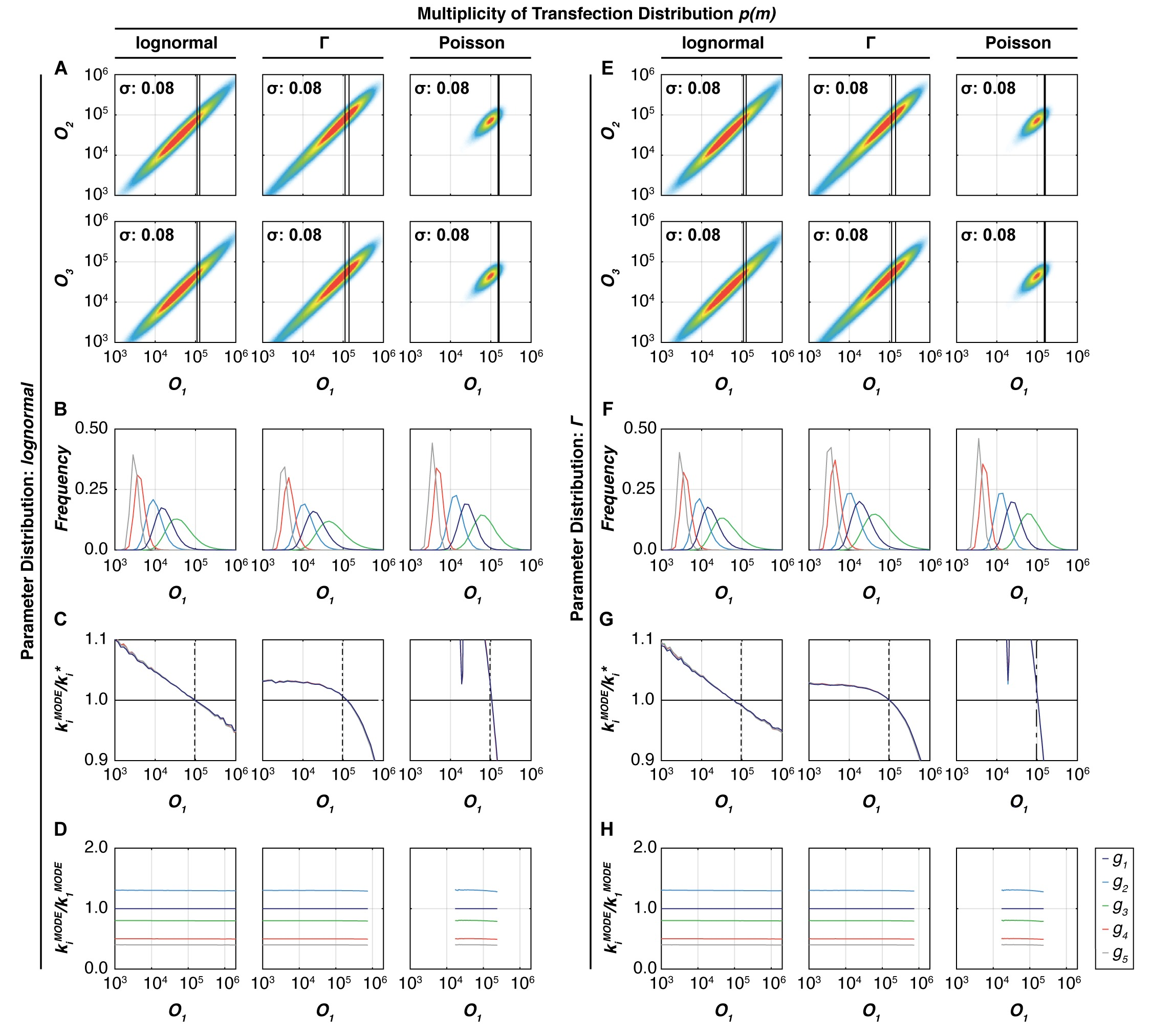

Supplement: S5 Fig — (TIF) [file pcbi.1008389.s010.tif]

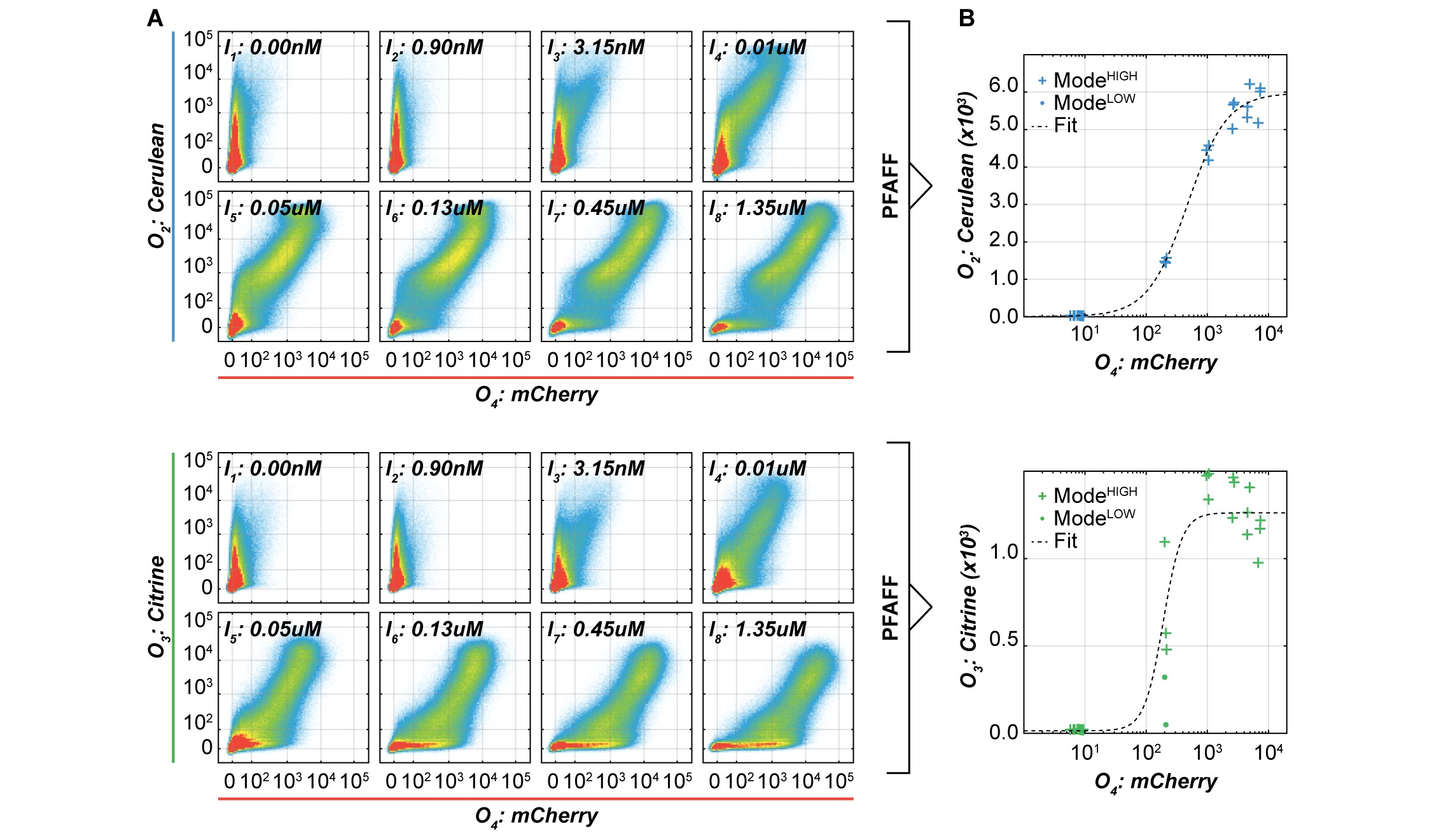

Supplement: S6 Fig — (TIF) [file pcbi.1008389.s011.tif]

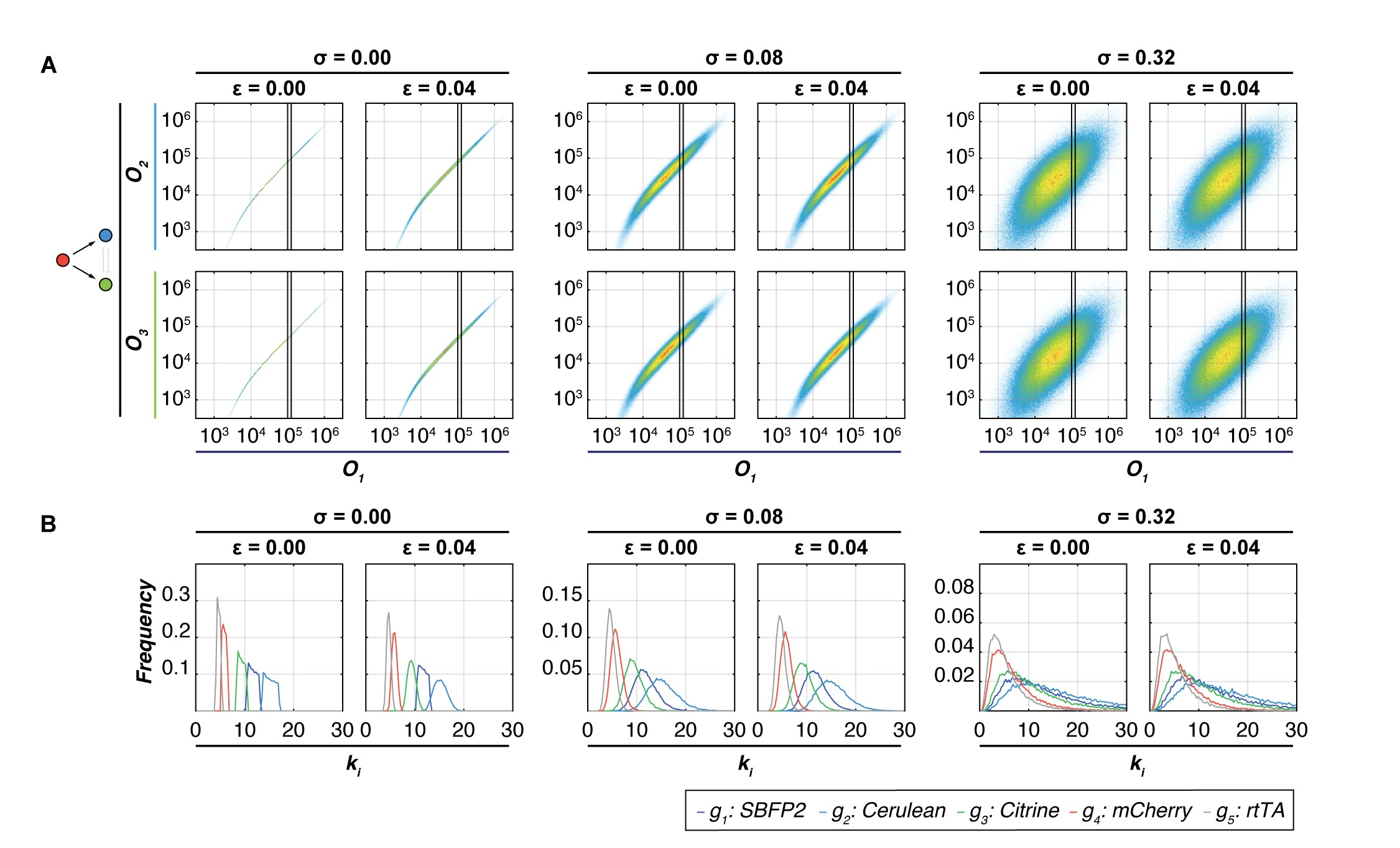

Supplement: S7 Fig — (TIF) [file pcbi.1008389.s012.tif]

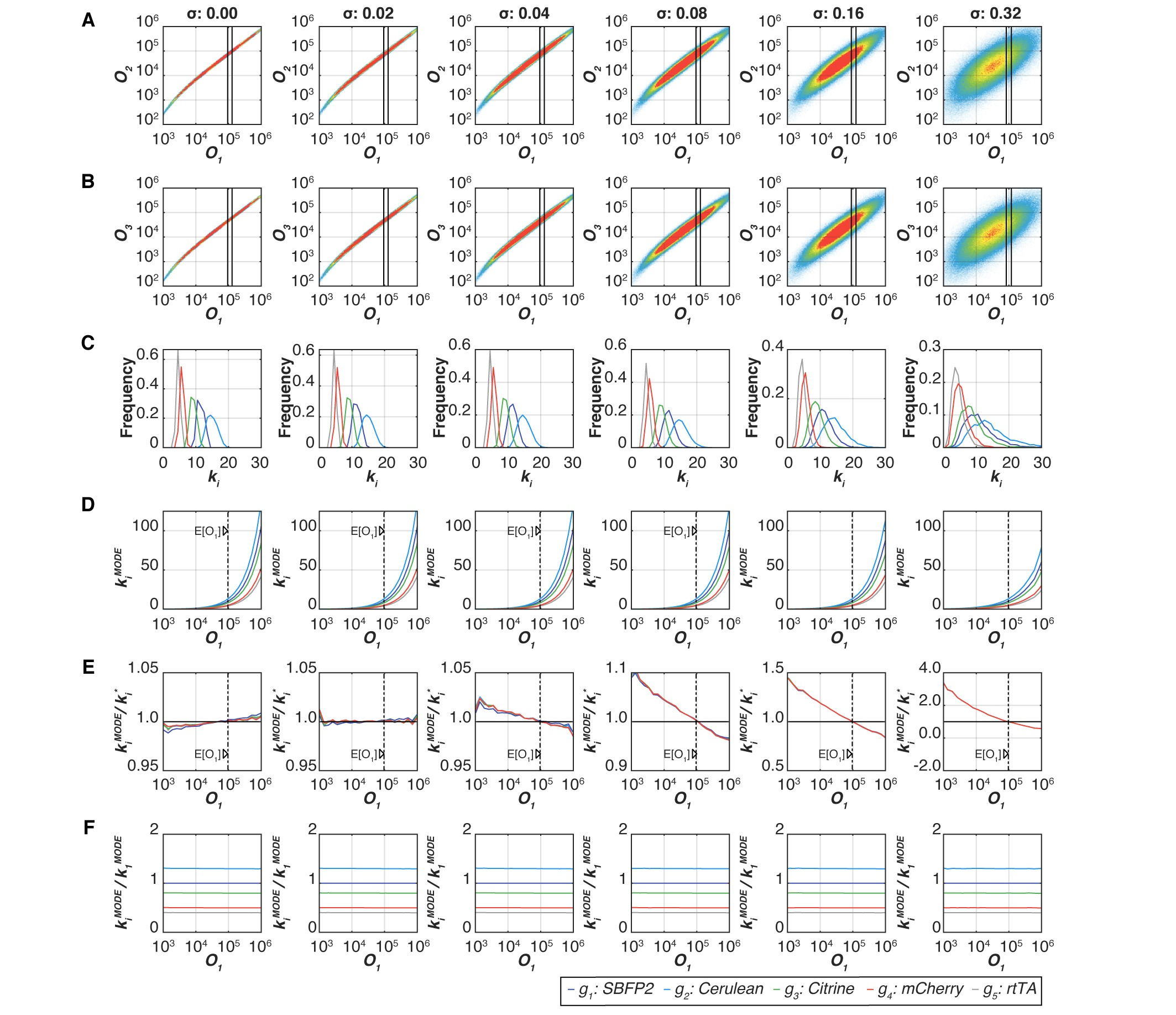

Supplement: S8 Fig — (TIF) [file pcbi.1008389.s013.tif]

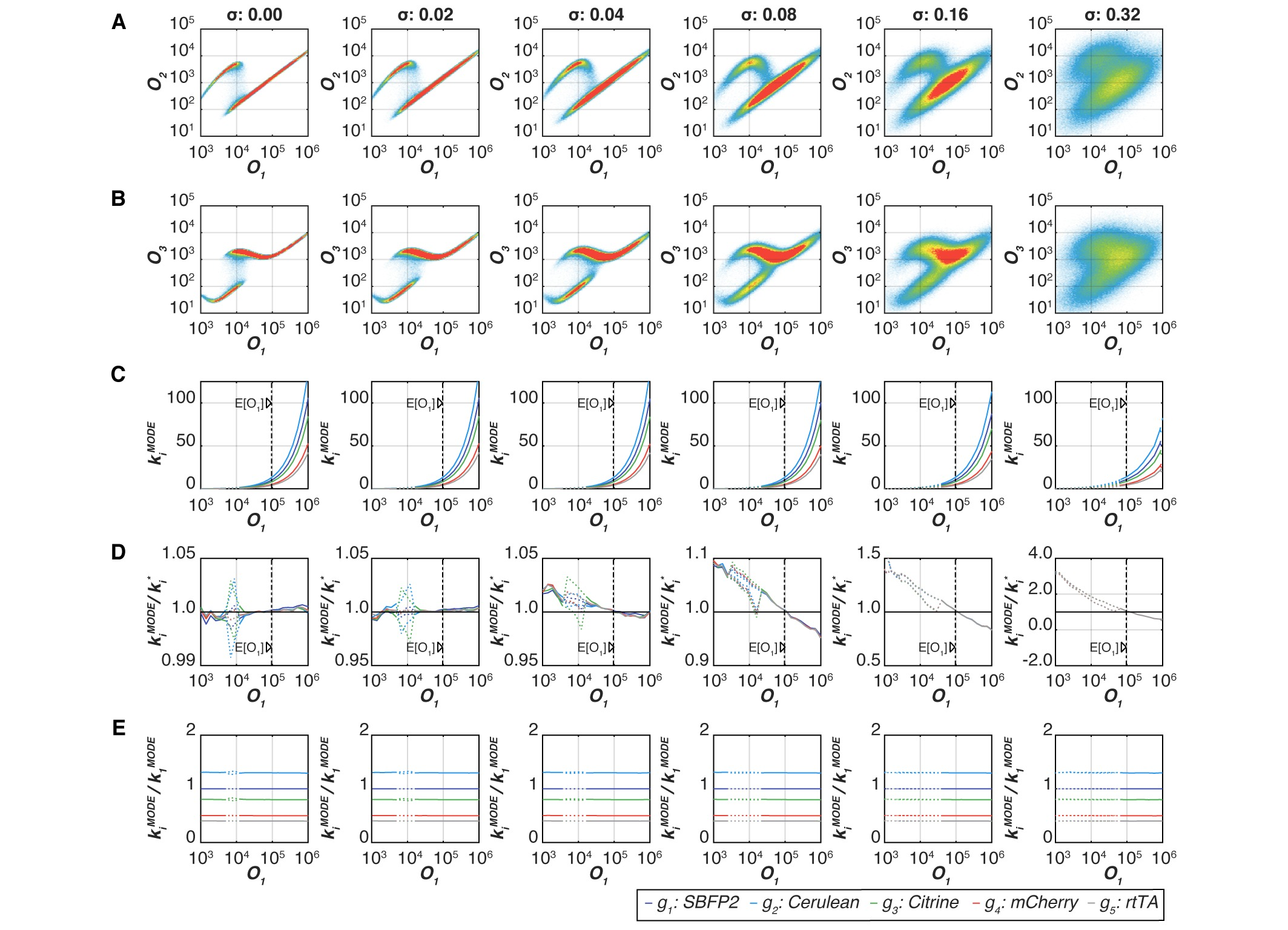

Supplement: S9 Fig — (TIF) [file pcbi.1008389.s014.tif]

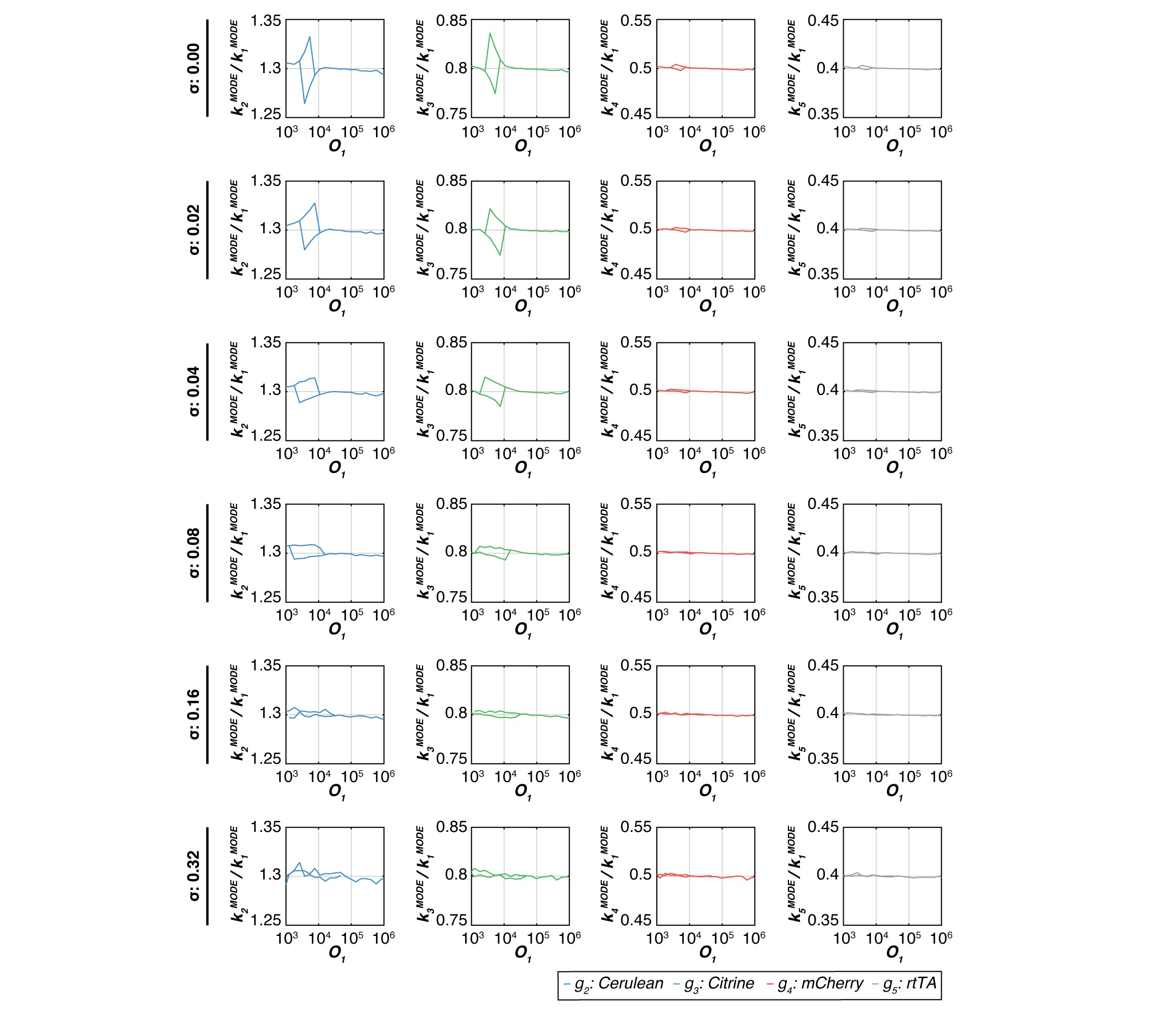

Supplement: S10 Fig — (TIF) [file pcbi.1008389.s015.tif]

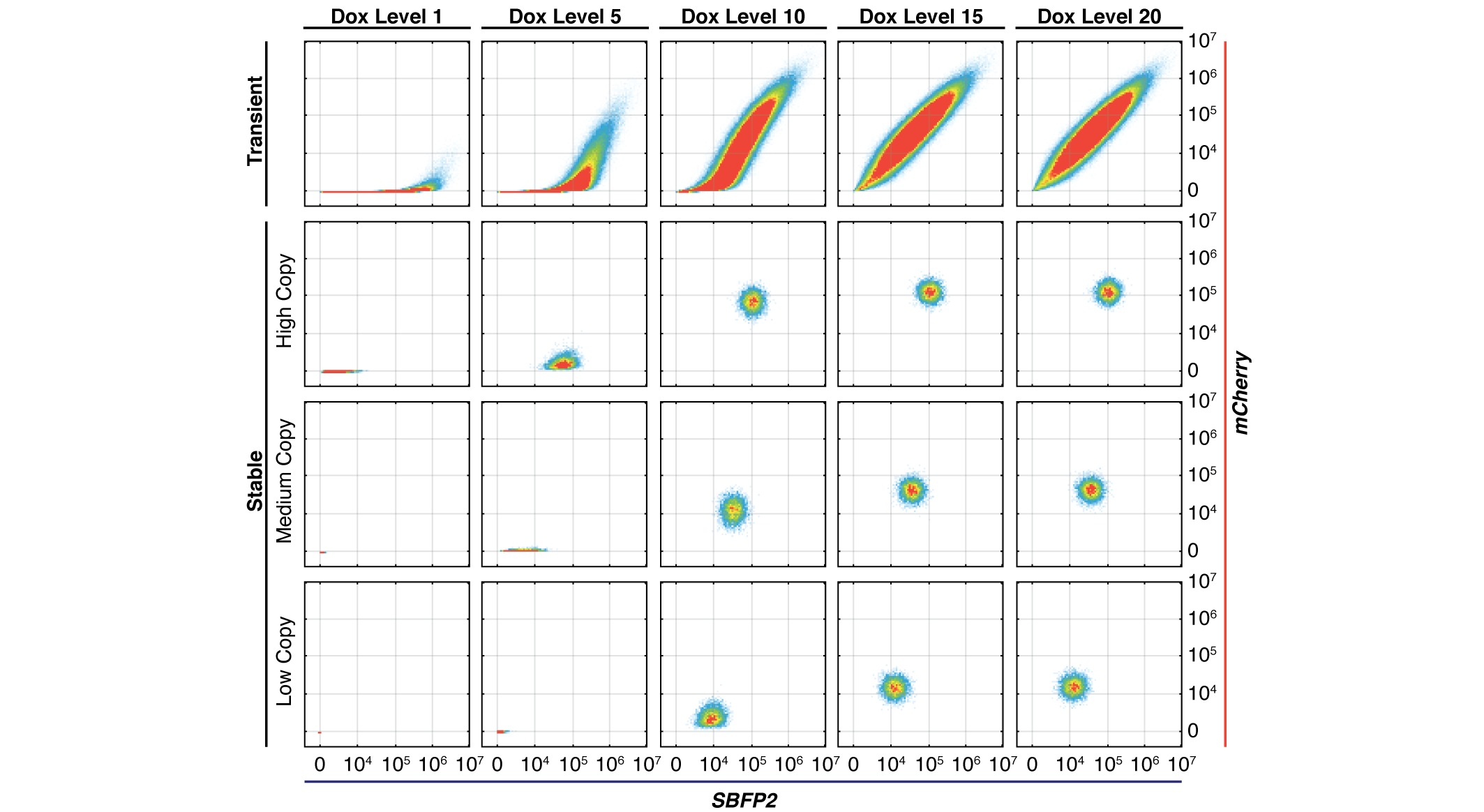

Supplement: S11 Fig — (TIF) [file pcbi.1008389.s016.tif]

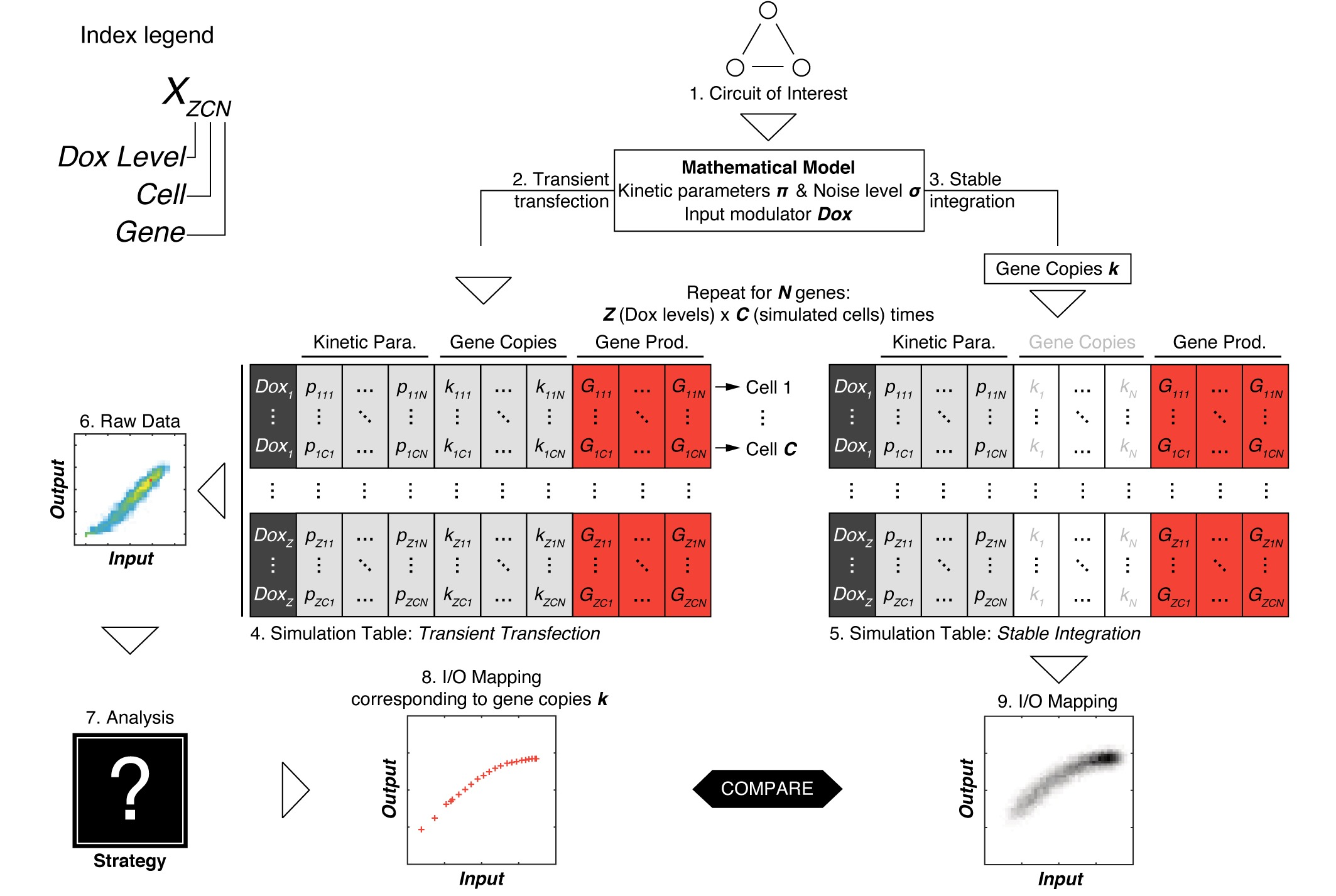

Supplement: S12 Fig — (TIF) [file pcbi.1008389.s017.tif]

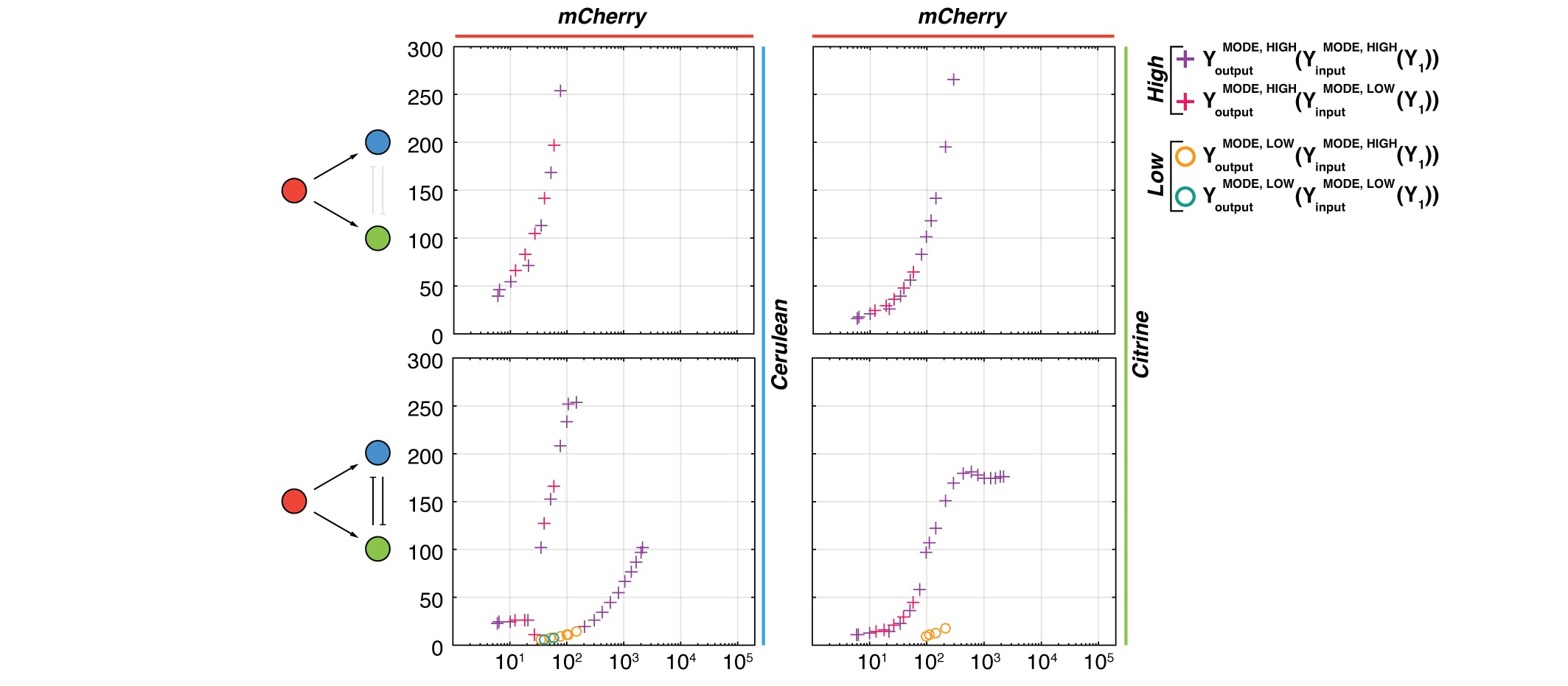

Supplement: S13 Fig — (TIF) [file pcbi.1008389.s018.tif]

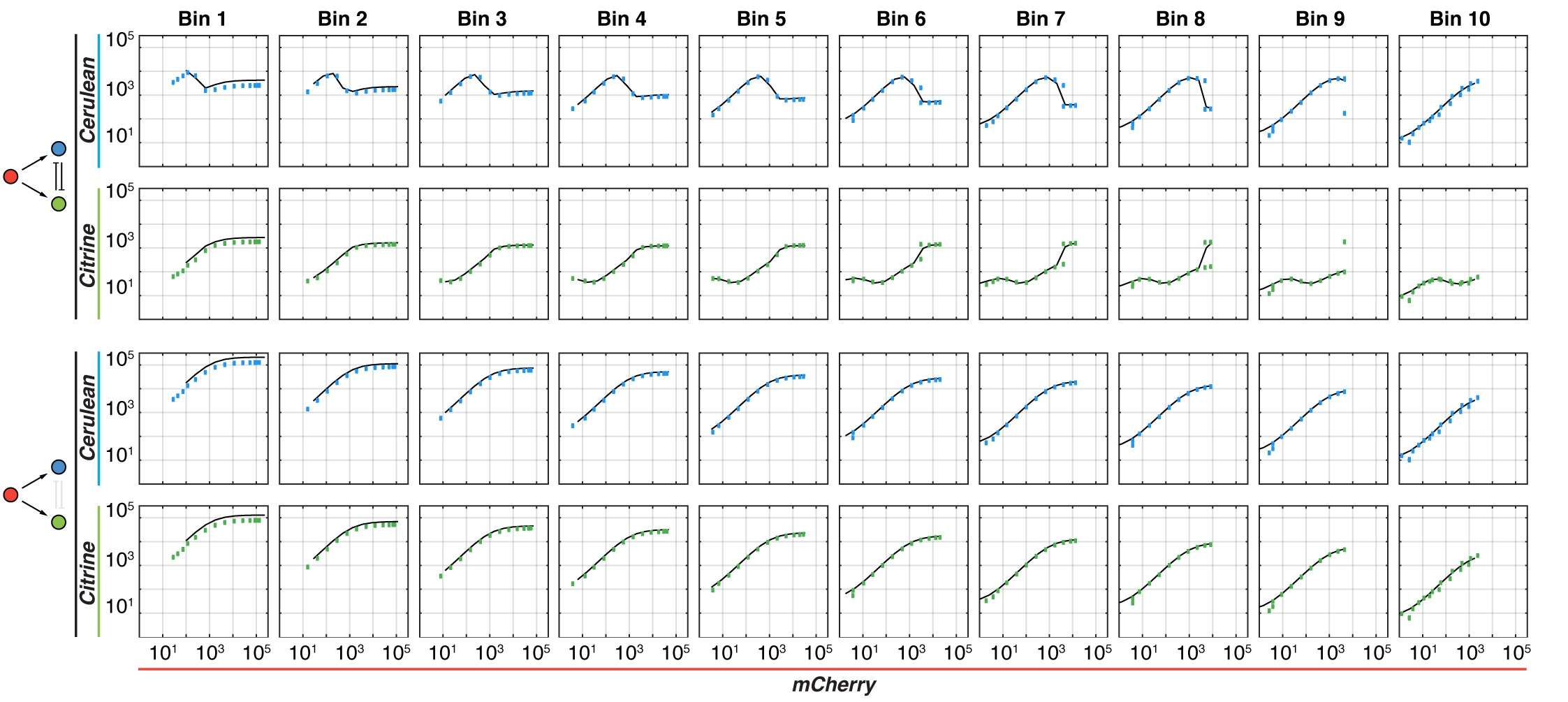

Supplement: S14 Fig — (TIF) [file pcbi.1008389.s019.tif]

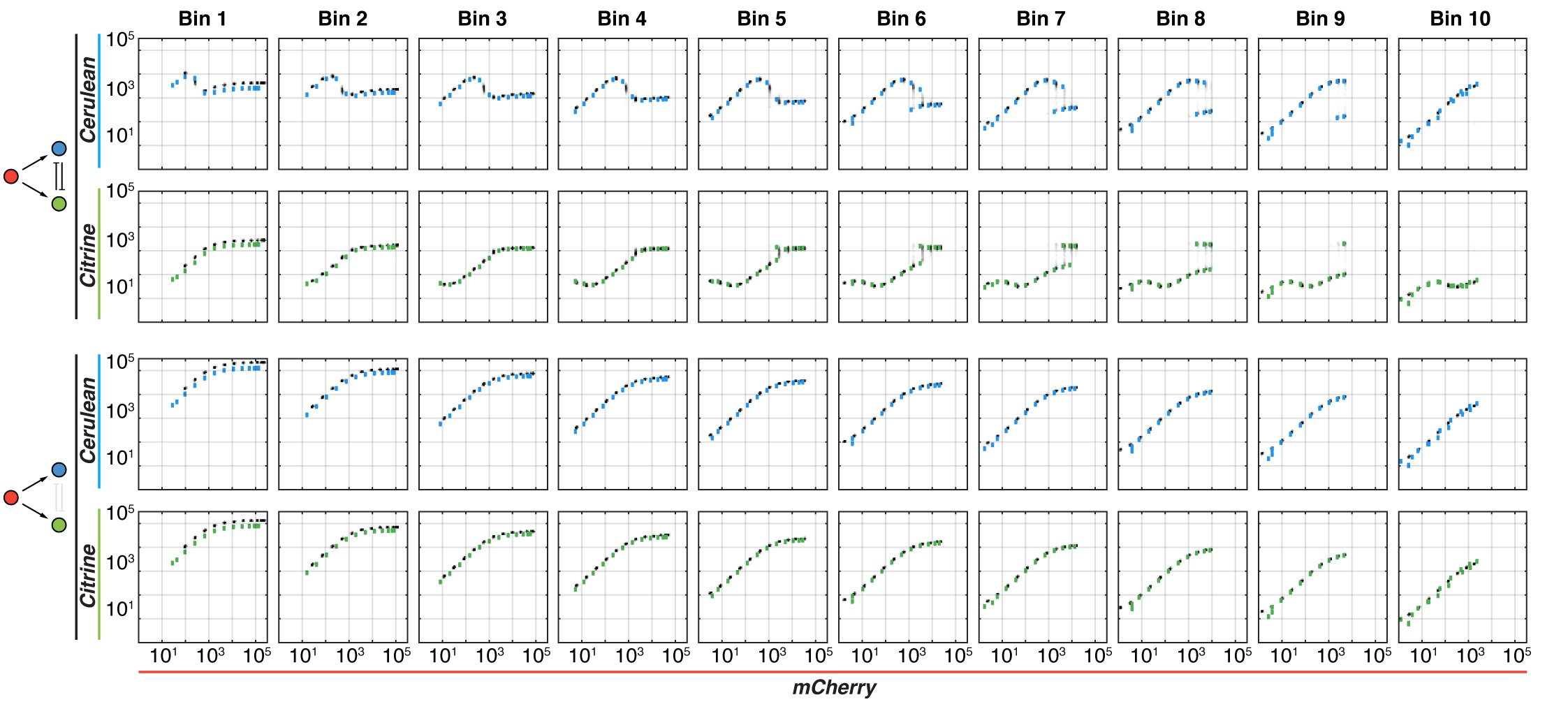

Supplement: S15 Fig — (TIF) [file pcbi.1008389.s020.tif]

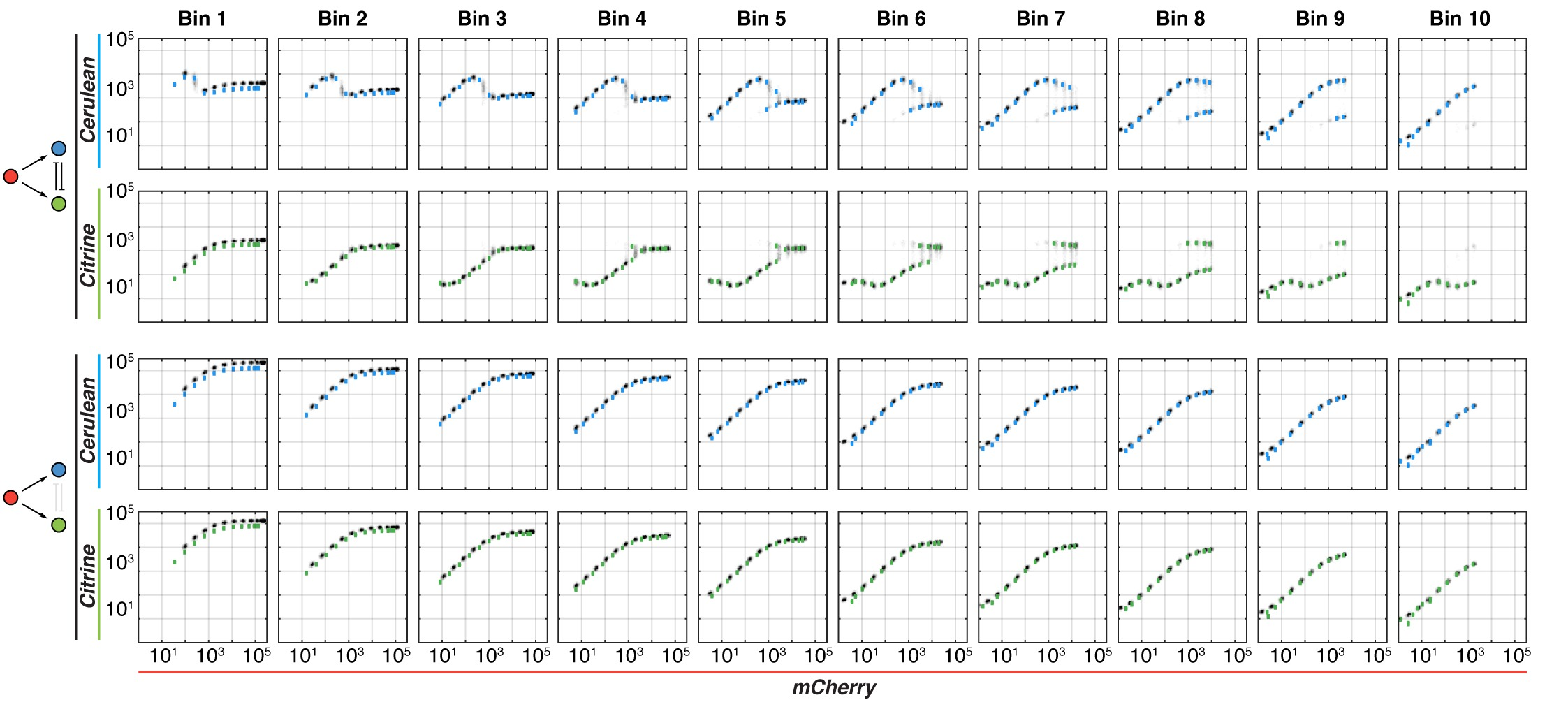

Supplement: S16 Fig — (TIF) [file pcbi.1008389.s021.tif]

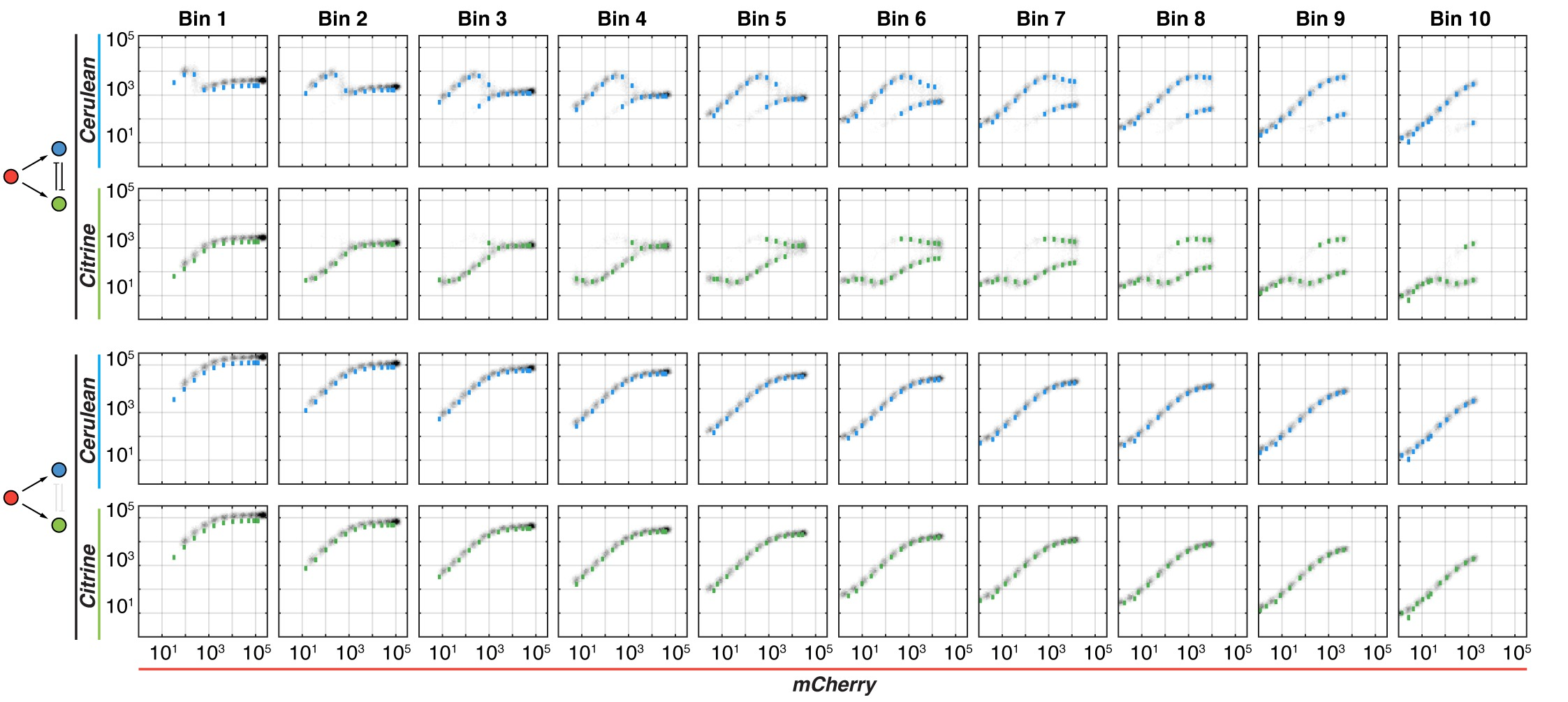

Supplement: S17 Fig — (TIF) [file pcbi.1008389.s022.tif]

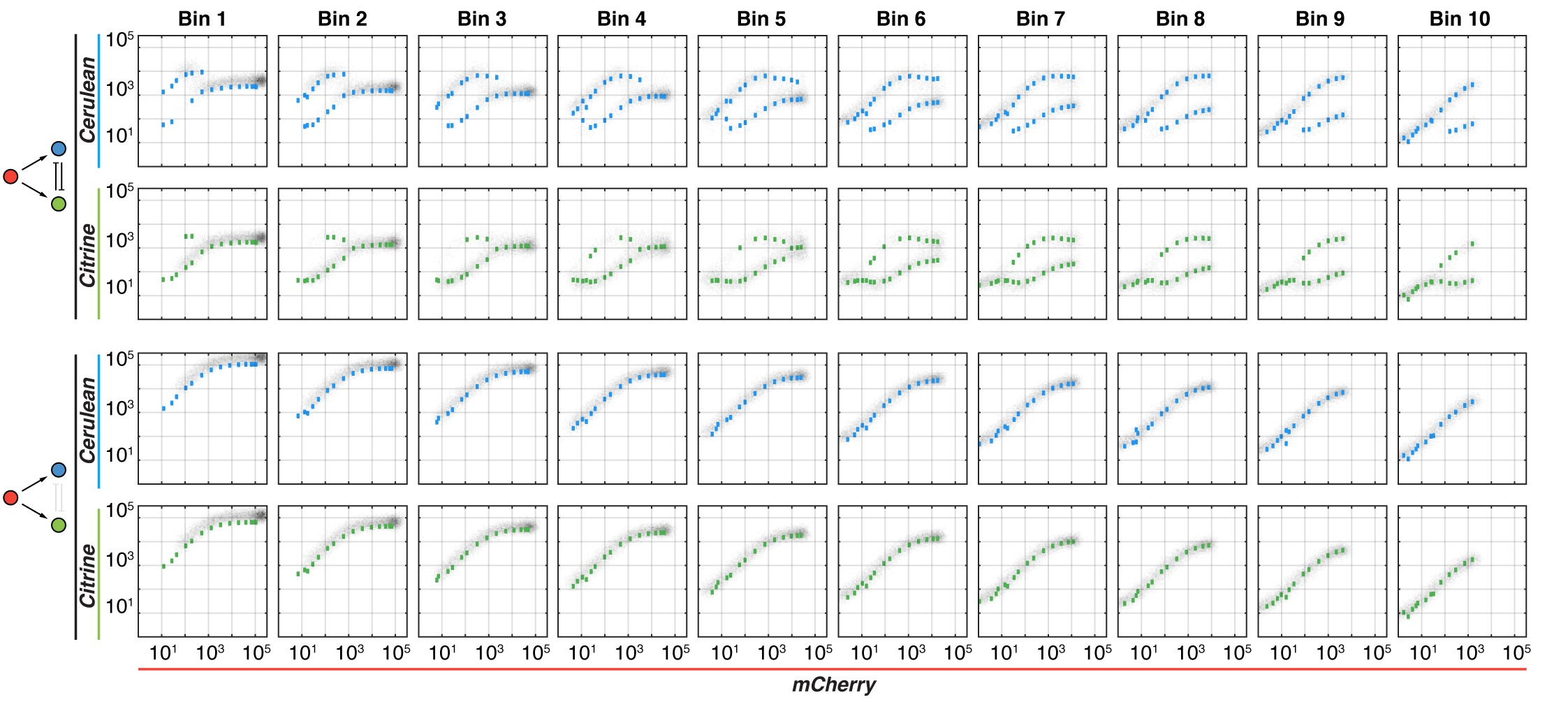

Supplement: S18 Fig — (TIF) [file pcbi.1008389.s023.tif]

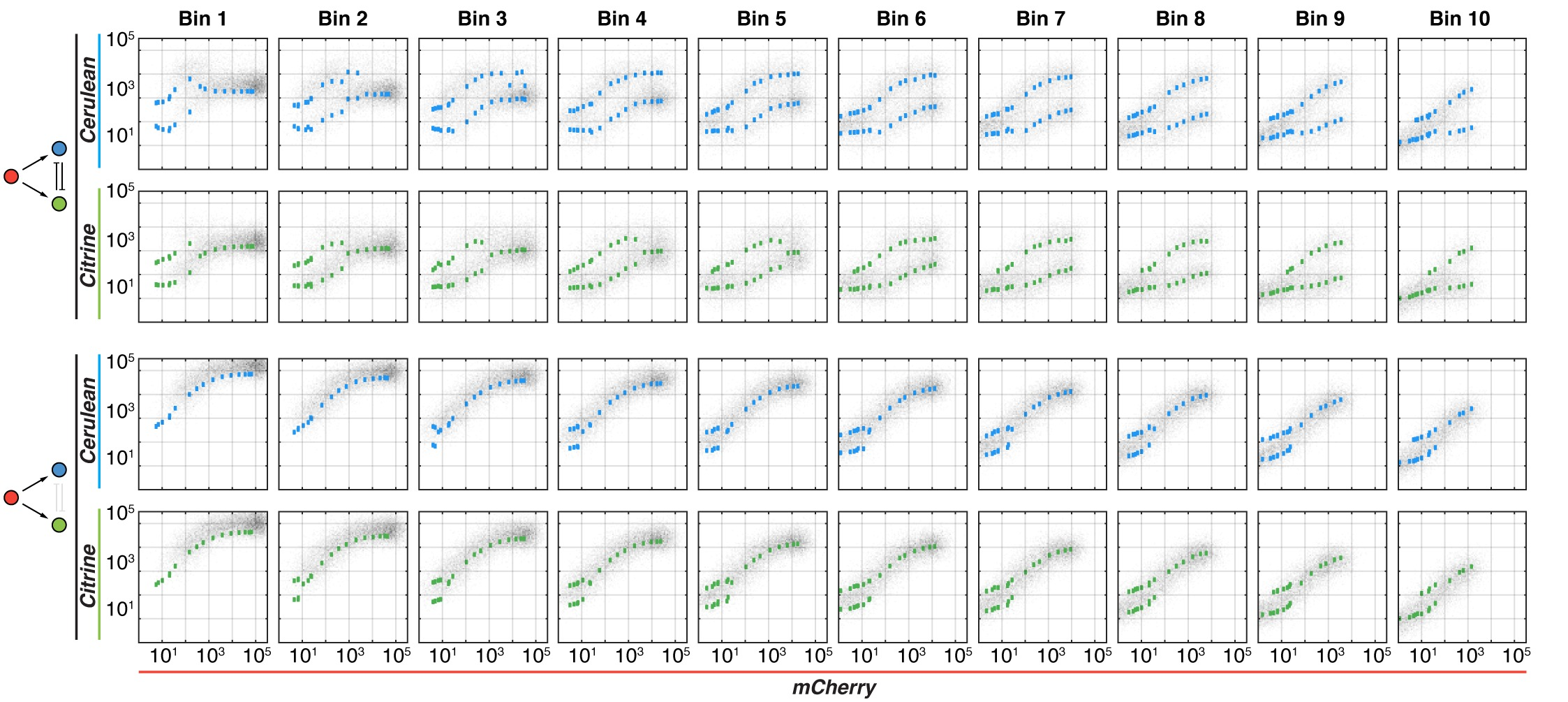

Supplement: S19 Fig — (TIF) [file pcbi.1008389.s024.tif]

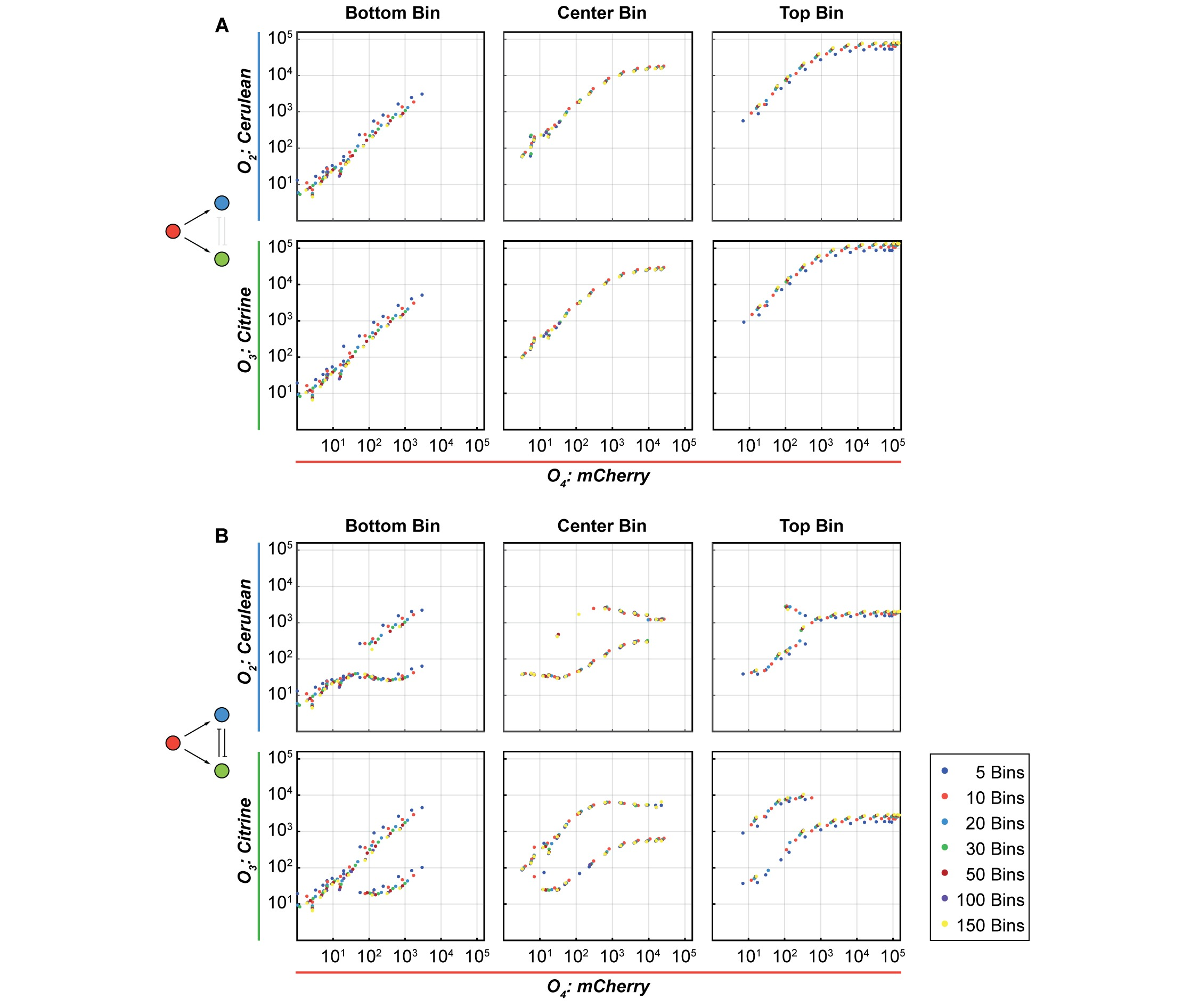

Supplement: S20 Fig — (TIF) [file pcbi.1008389.s025.tif]

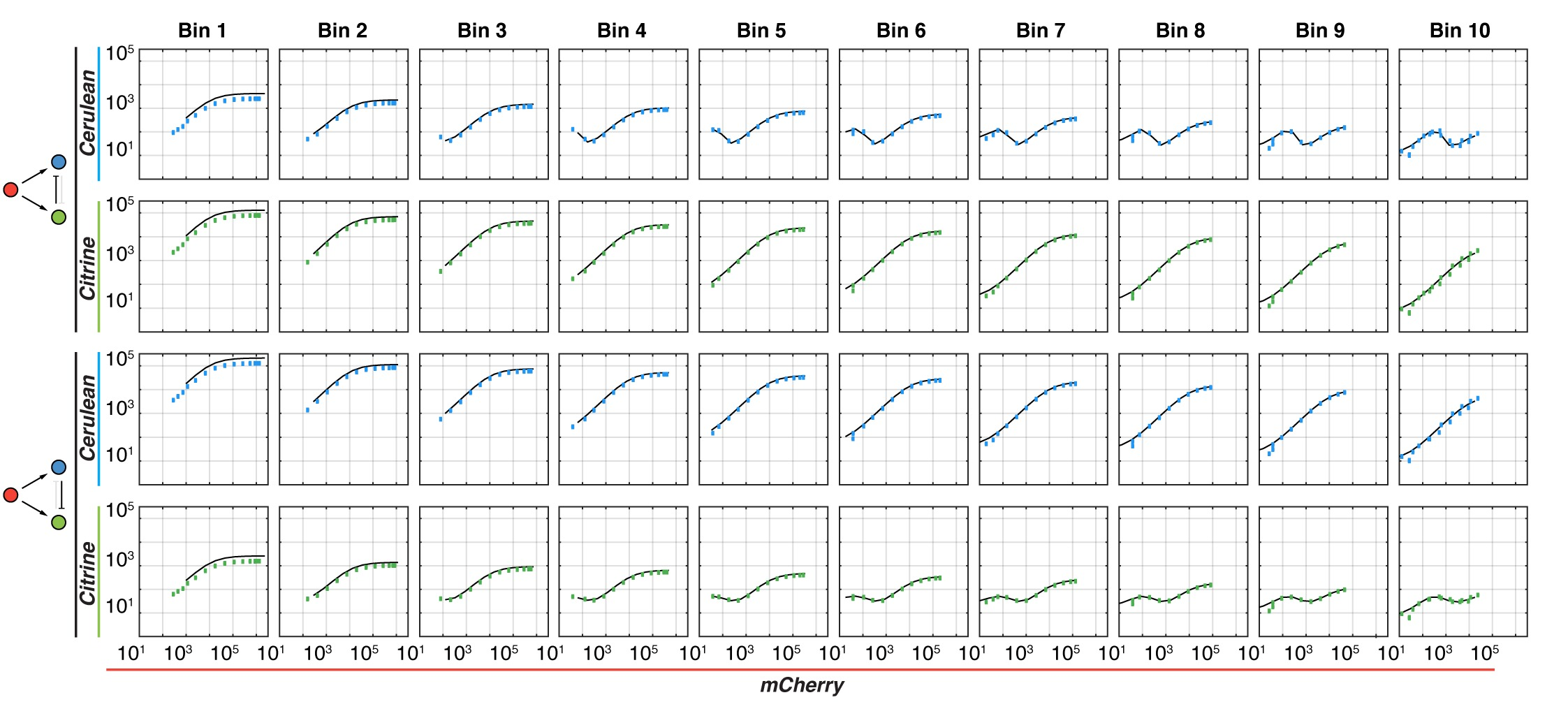

Supplement: S21 Fig — (TIF) [file pcbi.1008389.s026.tif]

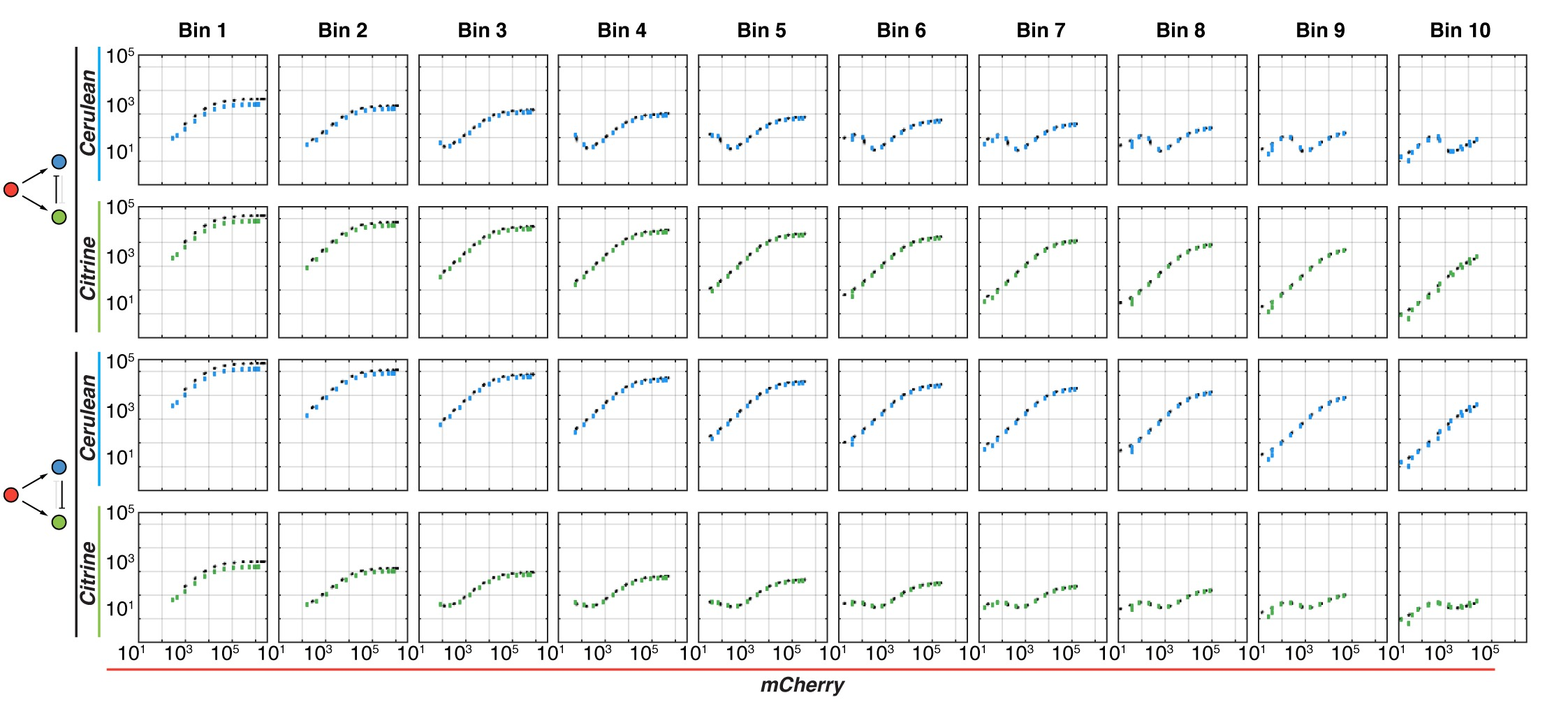

Supplement: S22 Fig — (TIF) [file pcbi.1008389.s027.tif]

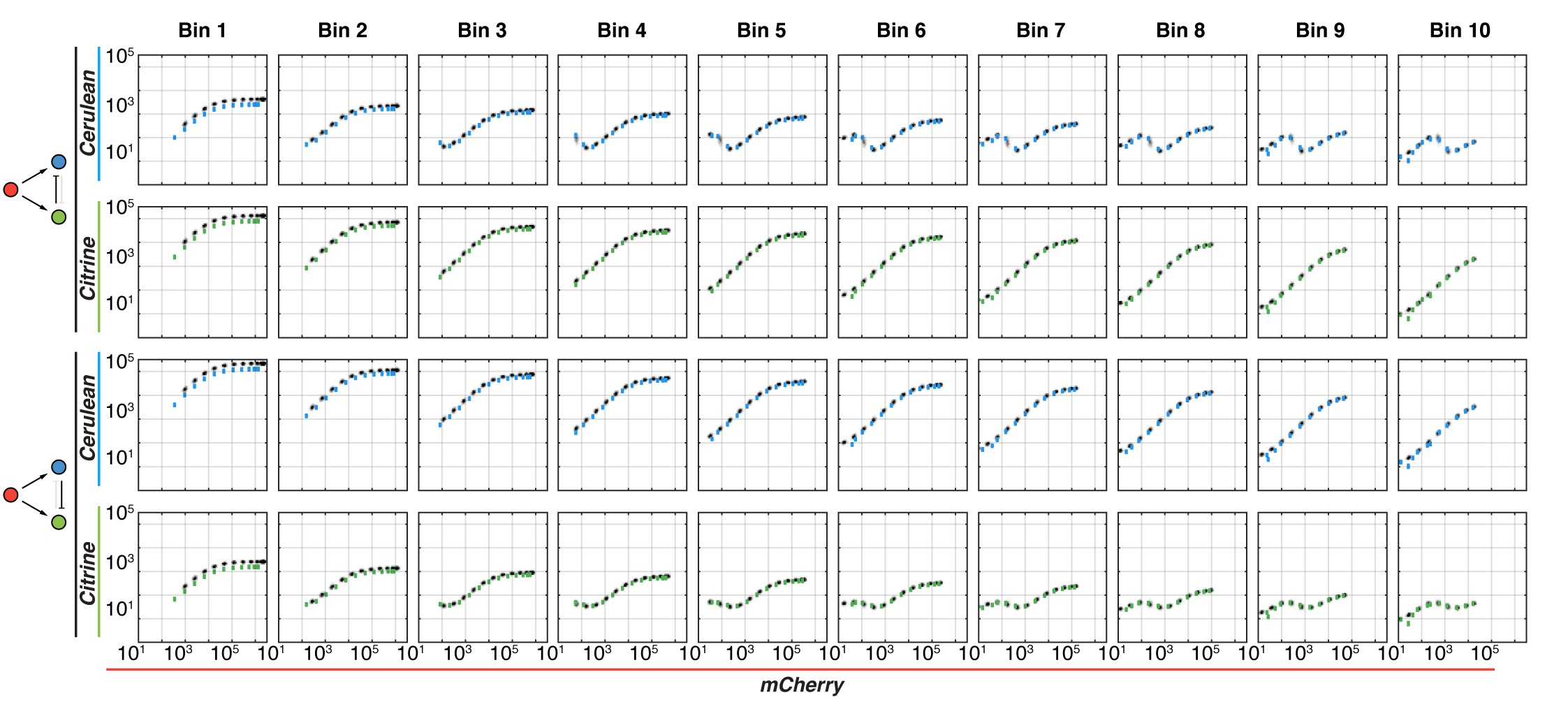

Supplement: S23 Fig — (TIF) [file pcbi.1008389.s028.tif]

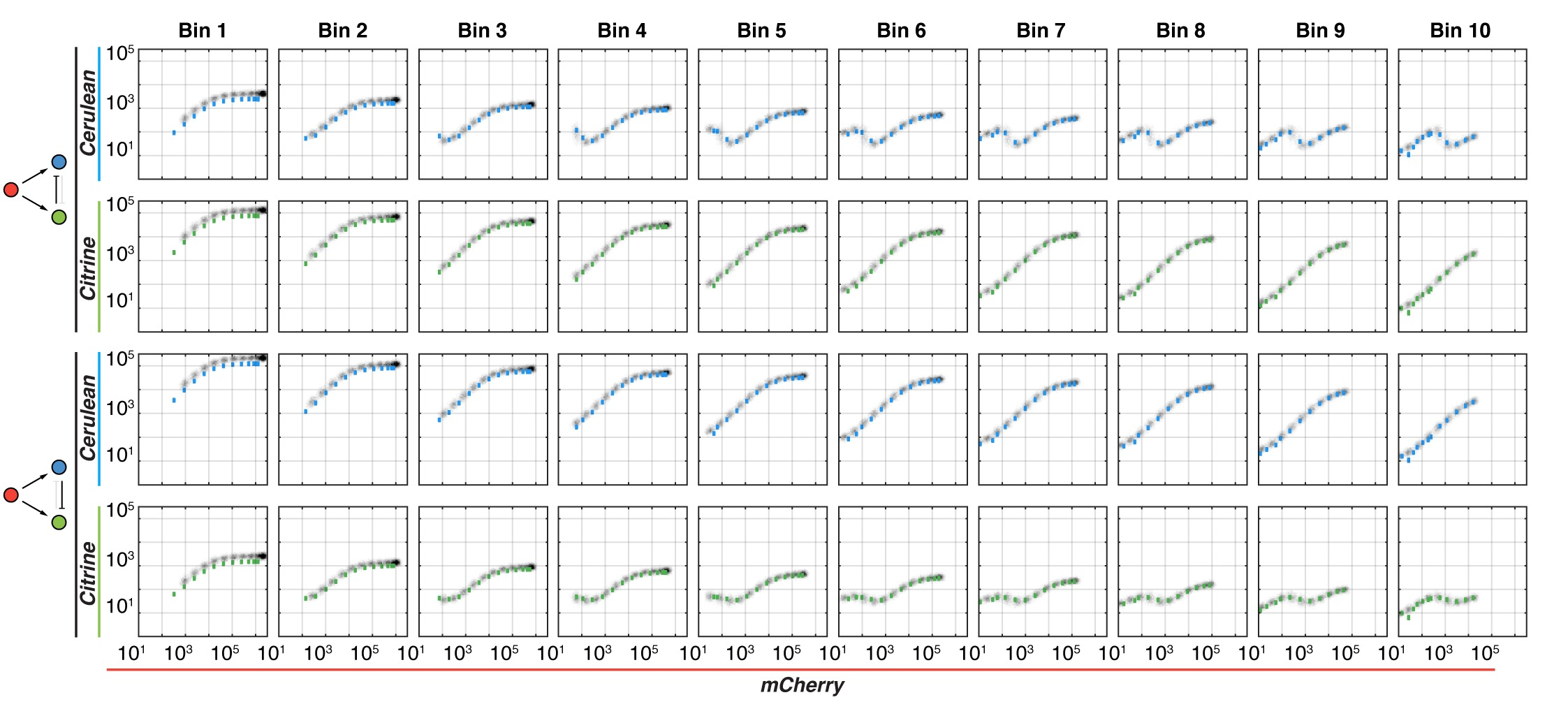

Supplement: S24 Fig — (TIF) [file pcbi.1008389.s029.tif]

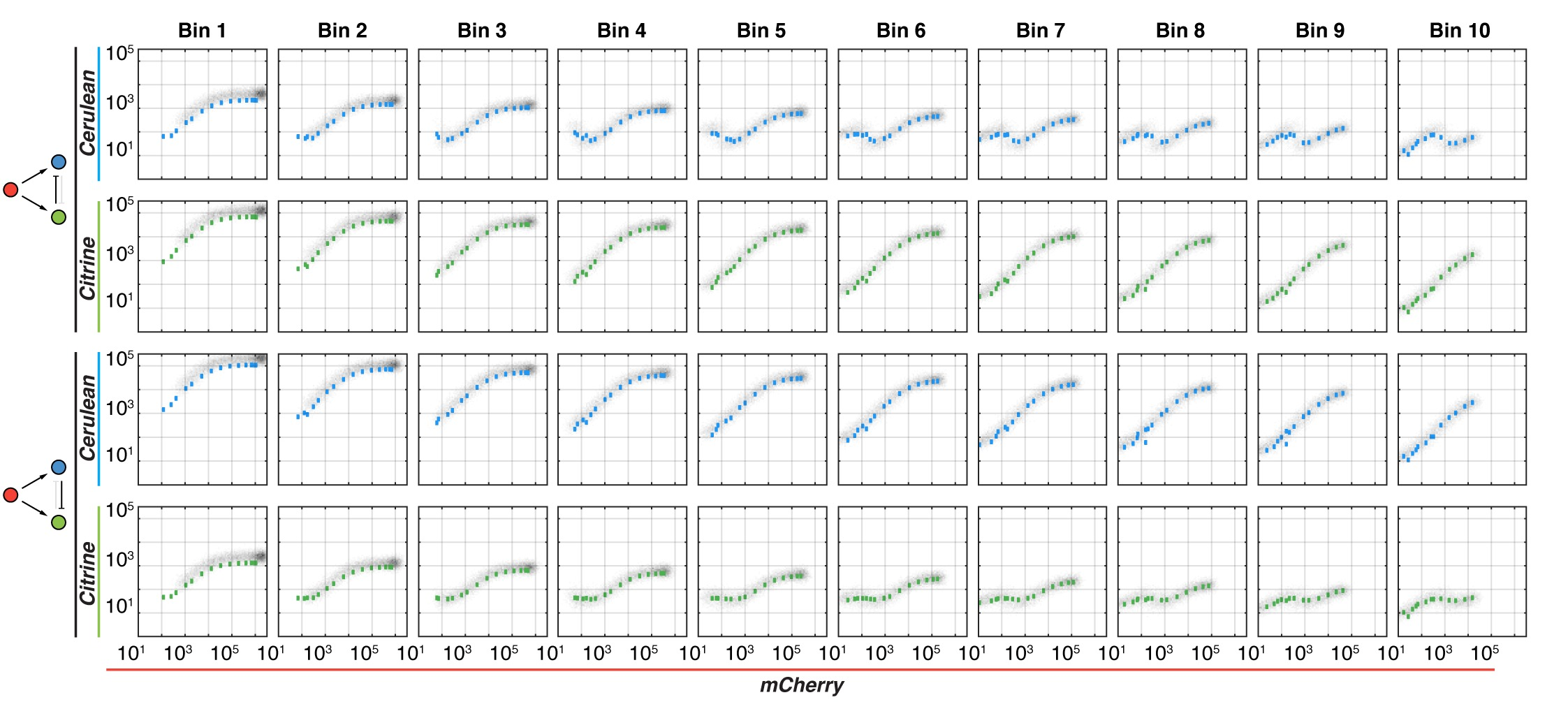

Supplement: S25 Fig — (TIF) [file pcbi.1008389.s030.tif]

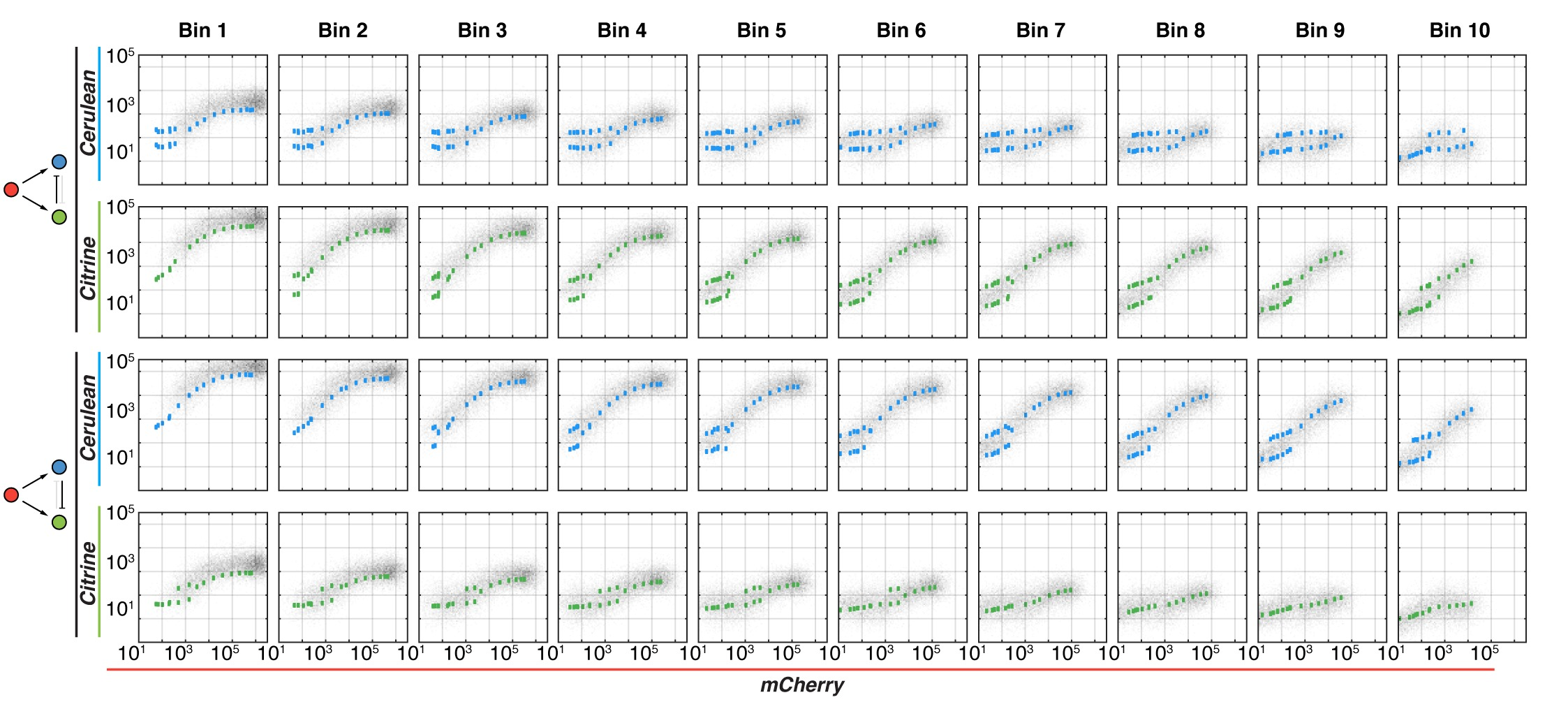

Supplement: S26 Fig — (TIF) [file pcbi.1008389.s031.tif]

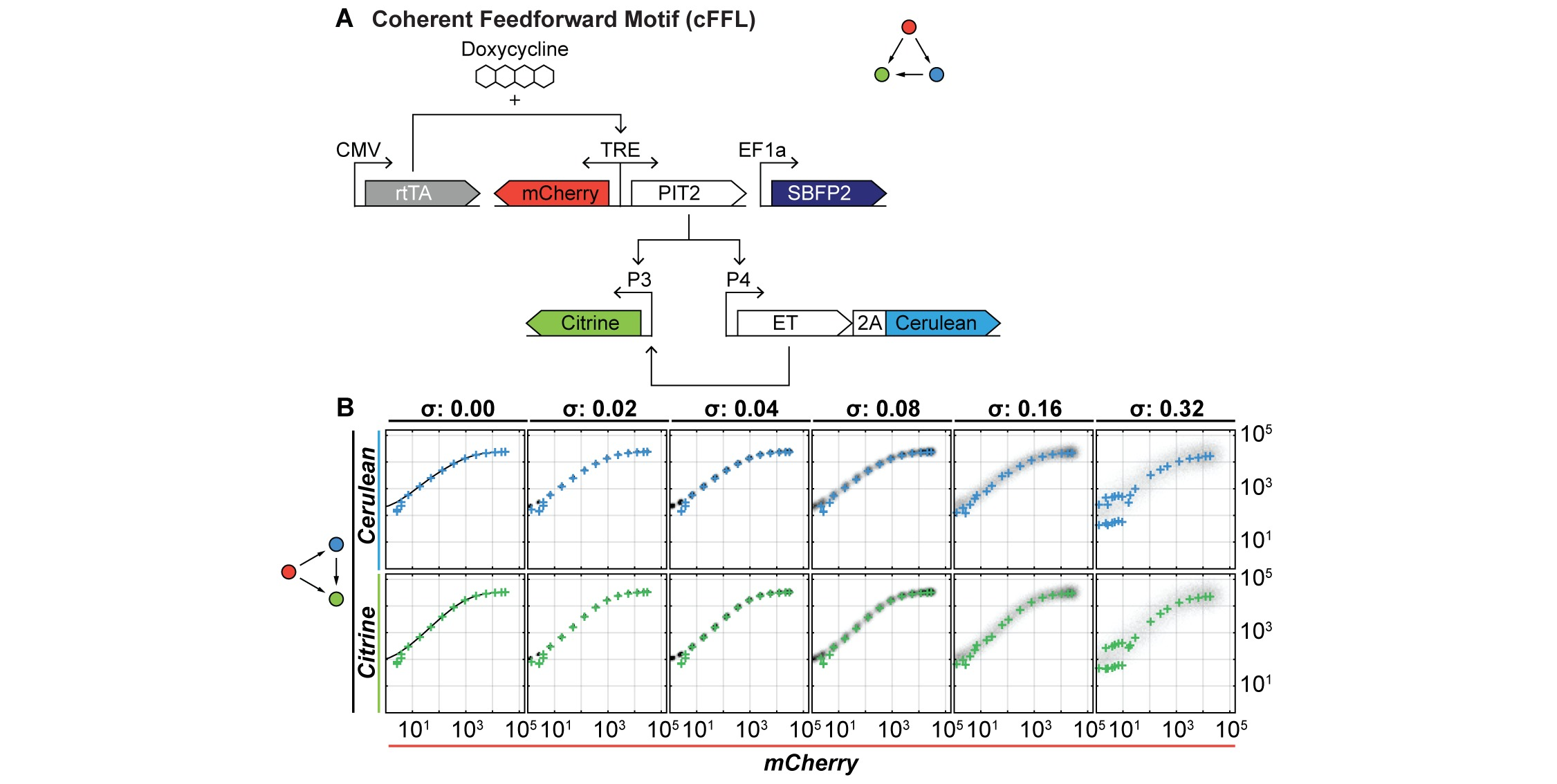

Supplement: S27 Fig — (TIF) [file pcbi.1008389.s032.tif]

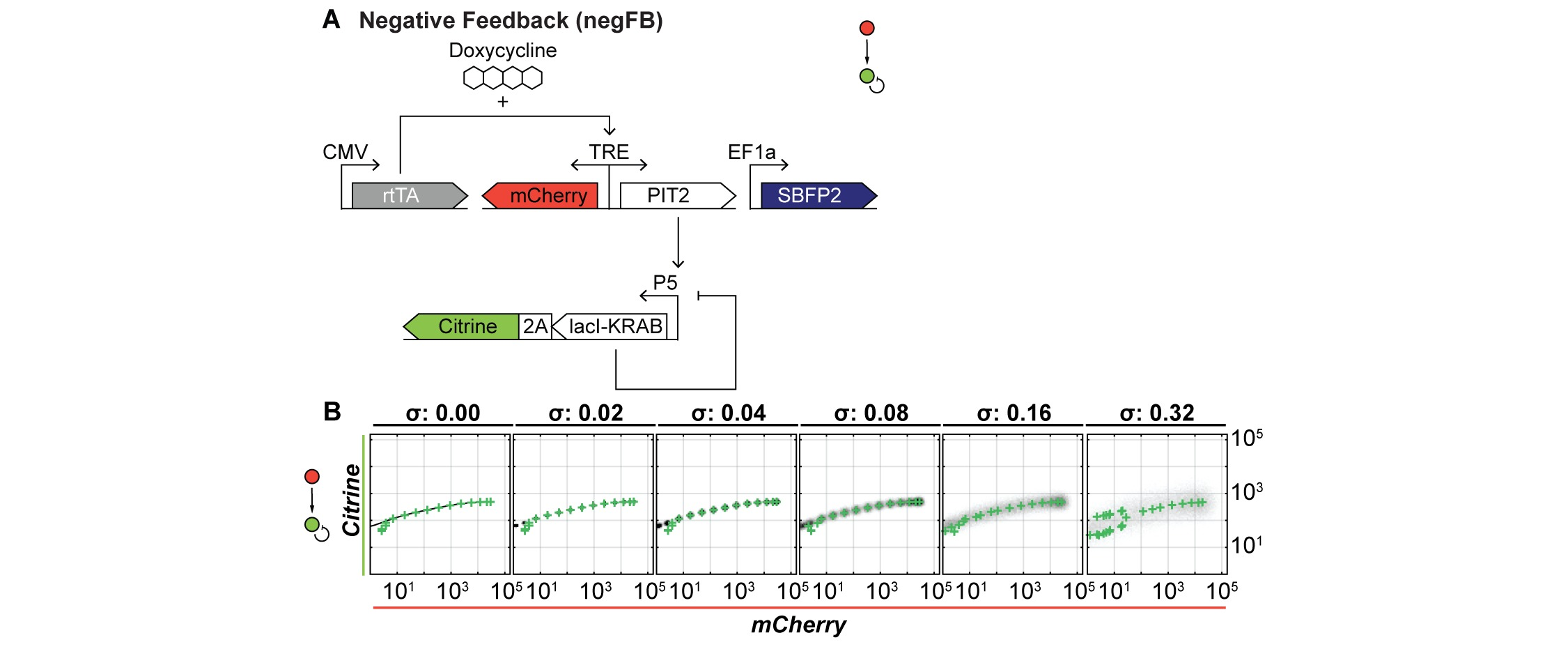

Supplement: S28 Fig — (TIF) [file pcbi.1008389.s033.tif]

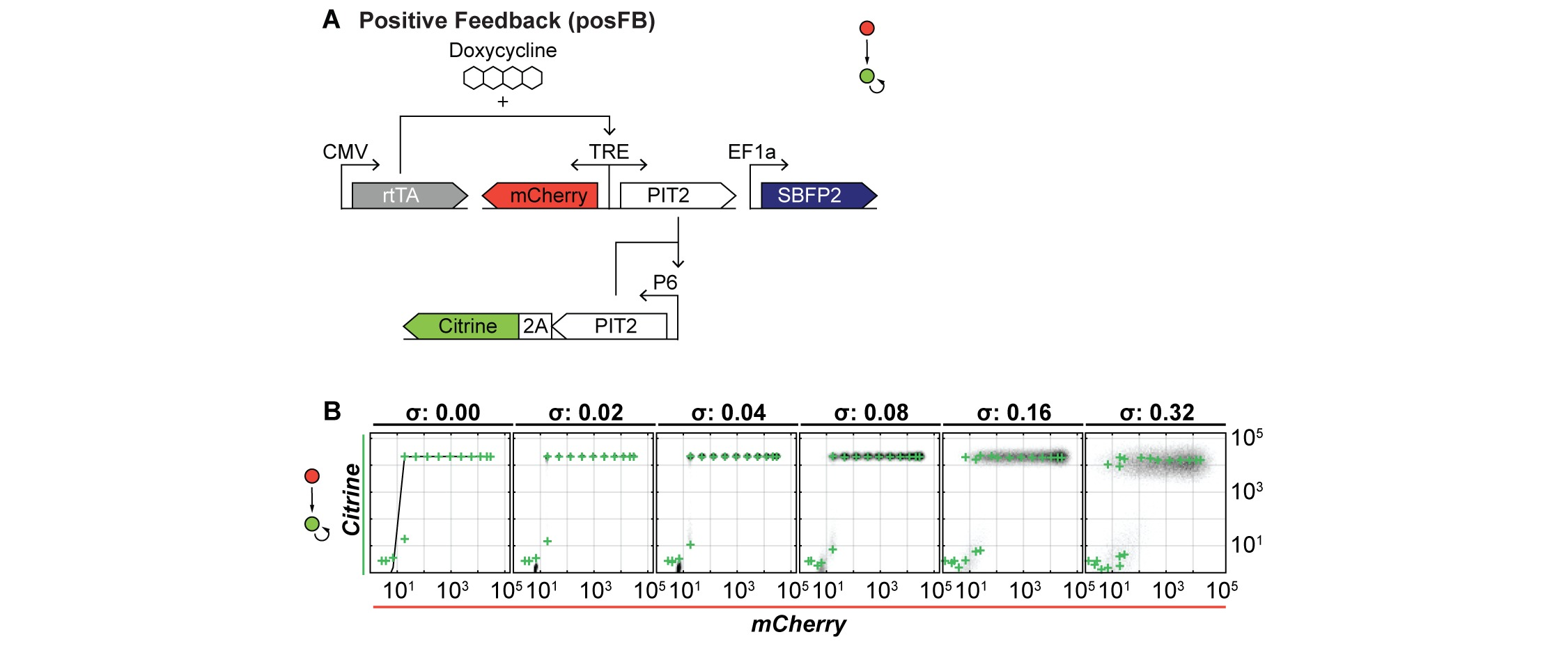

Supplement: S29 Fig — (TIF) [file pcbi.1008389.s034.tif]

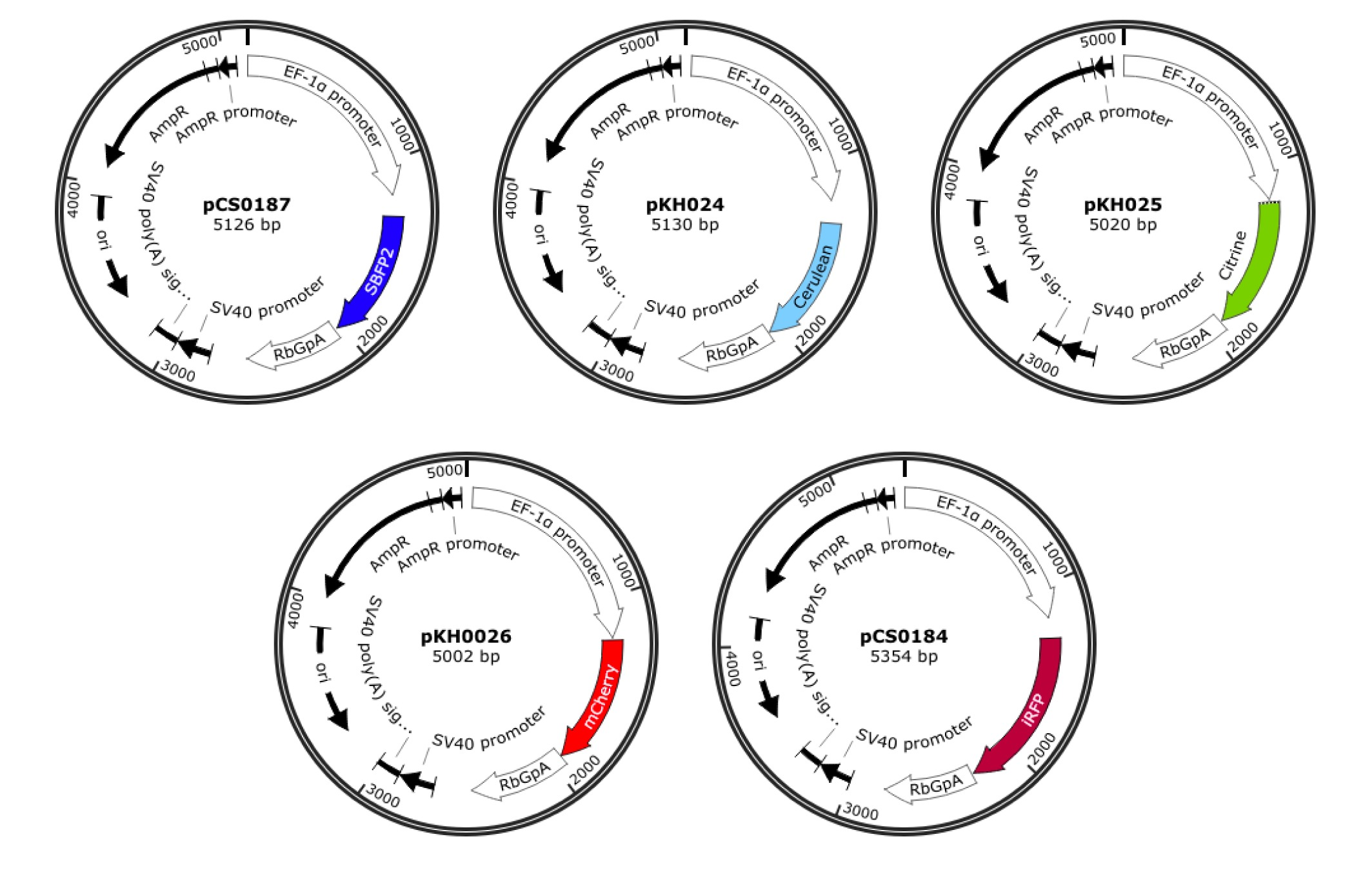

Supplement: S30 Fig — (TIF) [file pcbi.1008389.s035.tif]

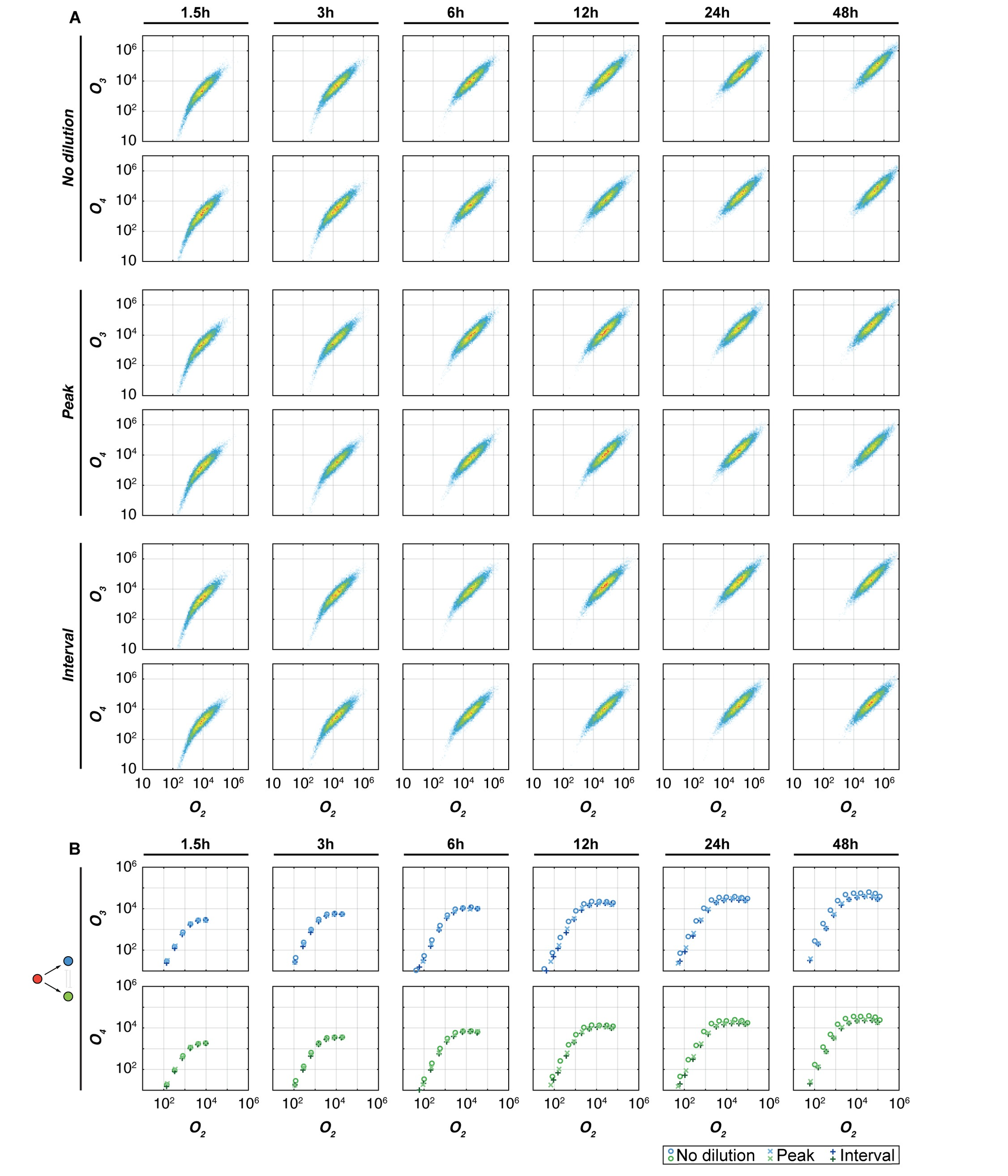

Supplement: S31 Fig — (TIF) [file pcbi.1008389.s036.tif]
