# Supplementary material for: Precise determination of input-output mapping for multimodal gene circuits using data from transient transfection
Source: PLoS Comput Biol. 2020 Nov 30;16(11):e1008389. doi: 10.1371/journal.pcbi.1008389 (PMC7728399; doi:10.1371/journal.pcbi.1008389)
Supplement: S1 Table — (DOCX) [file pcbi.1008389.s037.docx]

| **Parameter** | **Value** | **Unit** | **Reference** |
| --- | --- | --- | --- |
| $\beta_{1}$ | 9,572 | 1 | Set |
| $\beta_{2}$ | 5,403 | 1 | Set |
| $\beta_{3}$ | 5,403 | 1 | Set |
| $\beta_{4}$ | 16,733 | 1 | Set |
| $\beta_{5}$ | 9,115 | 1 | Set |
