# Supplementary material for: Precise determination of input-output mapping for multimodal gene circuits using data from transient transfection
Source: PLoS Comput Biol. 2020 Nov 30;16(11):e1008389. doi: 10.1371/journal.pcbi.1008389 (PMC7728399; doi:10.1371/journal.pcbi.1008389)
Supplement: S2 Table — (DOCX) [file pcbi.1008389.s038.docx]

| **Parameter** | **Value** | **Unit** | **Reference** |
| --- | --- | --- | --- |
| $\delta_{BFP}$ | 7.9e-6 | 1/s | Set |
| $\delta_{Cer}$ | 7.9e-6 | 1/s | Set |
| $\delta_{Chy}$ | 7.9e-6 | 1/s | Set |
| $\delta_{Cit}$ | 7.9e-6 | 1/s | Set |
| $\delta_{Kni}$ | 7.9e-6 | 1/s | Set |
| $\delta_{lac}$ | 7.9e-6 | 1/s | Set |
| $\delta_{ET}$ | 7.9e-6 | 1/s | Set |
| $\delta_{mRNA}$ | 0.000231 | 1/s | Set |
| $\delta_{PIT}$ | 7.9e-6 | 1/s | Set |
| $\delta_{rtTA}$ | 7.9e-6 | 1/s | Set |
| ${\delta_{rtTA}}_{DOX}$ | 7.9e-6 | 1/s | Set |
| $\delta_{DNA}$ | 0 | 1/s | Set |
| $k^{off}$ | 0.01 | 1/s | Set |
| $k_{DOX}^{off}$ | 0.01 | 1/s | Set |
| $k_{DOX}^{on}$ | 1e-9 | 1/(molecule*s) | Set |
| $k_{Kni}^{on}$ | 1.5e-6 | 1/(molecule*s)^2 | Set |
| $k_{lac}^{on}$ | 1e-9 | 1/(molecule*s)^4 | Set |
| $k_{P2_{PIT}}^{on}$ | 1e-5 | 1/(molecule*s) | Set |
| $k_{P1_{PIT}}^{on}$ | 1e-5 | 1/(molecule*s) | Set |
| $k_{P3}^{on}$ | 1e-5 | 1/(molecule*s) | Set |
| $k_{P4_{PIT}}^{on}$ | 1e-5 | 1/(molecule*s) | Set |
| $k_{P5_{PIT}}^{on}$ | 1e-5 | 1/(molecule*s) | Set |
| $k_{P6_{PIT}}^{on}$ | 1e-8 | 1/(molecule*s)^2 | Set |
| $k_{rtTA_{DOX}}^{on}$ | 4e-5 | 1/(molecule*s) | Set |
| $k_{P2}^{ts}$ | 1e-6 | 1/s | Set |
| $k_{P2_{Kni}}^{ts}$ | 1e-7 | 1/s | Set |
| $k_{P2_{PITKni}}^{ts}$ | 1e-5 | 1/s | Set |
| $k_{P2_{PIT}}^{ts}$ | 5e-4 | 1/s | Set |
| $k_{P4}^{ts}$ | 1e-6 | 1/s | Set |
| $k_{P4_{PIT}}^{ts}$ | 5e-4 | 1/s | Set |
| $k_{P1}^{ts}$ | 1e-6 | 1/s | Set |
| $k_{P1_{lac}}^{ts}$ | 1e-7 | 1/s | Set |
| $k_{P1_{PITlac}}^{ts}$ | 1e-5 | 1/s | Set |
| $k_{P1_{PIT}}^{ts}$ | 5e-4 | 1/s | Set |
| $k_{P4}^{ts}$ | 1e-6 | 1/s | Set |
| $k_{P4_{PIT}}^{ts}$ | 5e-4 | 1/s | Set |
| $k_{P4_{ET}}^{ts}$ | 1e-4 | 1/s | Set |
| $k_{P4_{PIT\_ET}}^{ts}$ | 6e-4 | 1/s | Set |
| $k_{P5}^{ts}$ | 1e-6 | 1/s | Set |
| $k_{P5_{PIT}}^{ts}$ | 5e-4 | 1/s | Set |
| $k_{P5_{lac}}^{ts}$ | 1e-7 | 1/s | Set |
| $k_{P5_{PIT\_lac}}^{ts}$ | 1e-5 | 1/s | Set |
| $k_{P6}^{ts}$ | 0 | 1/s | Set |
| $k_{P6_{PIT}}^{ts}$ | 5e-4 | 1/s | Set |
| $k_{{{200}_{rtTA}}_{DOX}}^{ts}$ | 1.5e-3 | 1/s | Set |
| $k_{rtTA}^{ts}$ | 8.3e-4 | 1/s | Set |
| $k_{BFP}^{ts}$ | 8.3e-4 | 1/s | Set |
| $\pi_{BFP}$ | 0.021 | 1/s | [1] |
| $\pi_{Cer}$ | 0.021 | 1/s | [1] |
| $\pi_{Chy}$ | 0.021 | 1/s | [1] |
| $\pi_{Cit}$ | 0.021 | 1/s | [1] |
| $\pi_{Kni}$ | 0.012 | 1/s | [1] |
| $\pi_{lac}$ | 0.01 | 1/s | [1] |
| $\pi_{PIT}$ | 0.021 | 1/s | [1] |
| $\pi_{rtTA}$ | 0.02 | 1/s | [1] |
| $\pi_{ET}$ | 0.01 | 1/s | [1] |
