# Supplementary material for: Precise determination of input-output mapping for multimodal gene circuits using data from transient transfection
Source: PLoS Comput Biol. 2020 Nov 30;16(11):e1008389. doi: 10.1371/journal.pcbi.1008389 (PMC7728399; doi:10.1371/journal.pcbi.1008389)
Supplement: S3 Table — (DOCX) [file pcbi.1008389.s039.docx]

| **Plasmid** | **Reference** |
| --- | --- |
| Ef1a-SBFP2 (pCS187) | SBFP2 was amplified from pKB002 with primers PR2263 & PR2272 and cloned between restriction sites BmtI/XbaI of pKH25 |
| Ef1a-Cerulean (pKH24) | see Prochazka et al [2] |
| Ef1a-Citrine (pKH25) | see Prochazka et al [2] |
| Ef1a-mCherry (pKH26) | see Prochazka et al [2] |
| Ef1a-iRFP (pCS184) | SBFP2 was amplified from Addgene plasmid 31857 with primers PR2258 & PR2259 and cloned between restriction sites BmtI/XbaI of pKH25 |
