# Supplementary material for: Precise determination of input-output mapping for multimodal gene circuits using data from transient transfection
Source: PLoS Comput Biol. 2020 Nov 30;16(11):e1008389. doi: 10.1371/journal.pcbi.1008389 (PMC7728399; doi:10.1371/journal.pcbi.1008389)
Supplement: S4 Table — (DOCX) [file pcbi.1008389.s040.docx]

| **Primer** | **Sequence** |
| --- | --- |
| PR2258 | aatgtgaaGCTAGCgccaccatggctgaaggatccgtcg |
| PR2259 | aatgtaaTCTAGAtcactcttccatcacgccgatc |
| PR2263 | aatgtgaaGCTAGCgccaccATGGTGAGCAAGGGCGAGGAG |
| PR2272 | aatgtaaTCTAGATTACTACTTGTACAACTCGTCCATACCC |
