# Supplementary material for: Precise determination of input-output mapping for multimodal gene circuits using data from transient transfection
Source: PLoS Comput Biol. 2020 Nov 30;16(11):e1008389. doi: 10.1371/journal.pcbi.1008389 (PMC7728399; doi:10.1371/journal.pcbi.1008389)
Supplement: S5 Table — (DOCX) [file pcbi.1008389.s041.docx]

| **Parameter** | **Value** | **Unit** | **Reference** |
| --- | --- | --- | --- |
| $k_{on}^{P_{2}}$ | 5.0e-9 | 1/(molecule*s)^2 | Set |
| $k_{off}$ | 0.01 | 1/s | Set |
| $\beta_{g_{1}}$ | 0.1 | 1/s | Set |
| $\beta_{g_{3}}$ | 0.1 | 1/s | Set |
| $\beta_{g_{4}}$ | 0.1 | 1/s | Set |
